# Functional properties of Candida albicans extracellular vesicles released in the presence of the antifungal drugs amphotericin B, fluconazole and caspofungin

**DOI:** 10.1099/mic.0.001565

**Published:** 2025-06-12

**Authors:** Kamila Kulig, Elzbieta Rudolphi-Szydlo, Anna Barbasz, Magdalena Surowiec, Ewelina Wronowska, Katarzyna Kowalik, Dorota Satala, Grazyna Bras, Olga Barczyk-Woznicka, Elzbieta Karnas, Elzbieta Pyza, Ewa Zuba-Surma, Maria Rapala-Kozik, Justyna Karkowska-Kuleta

**Affiliations:** 1 Department of Comparative Biochemistry and Bioanalytics, Faculty of Biochemistry, Biophysics and Biotechnology, Jagiellonian University, Gronostajowa 7, 30-387 Kraków, Poland; 2 Department of Biochemistry and Biophysics, Institute of Biology and Earth Sciences, University of the National Education Commission, Podchorazych 2, 30-084 Kraków, Poland; 3 Doctoral School of Exact and Natural Sciences, Faculty of Biochemistry, Biophysics and Biotechnology, Jagiellonian University, Gronostajowa 7, 30-387 Kraków, Poland; 4 Department of Cell Biology and Imaging, Institute of Zoology and Biomedical Research, Jagiellonian University, Gronostajowa 9, 30-387 Kraków, Poland; 5 Department of Cell Biology, Faculty of Biochemistry, Biophysics and Biotechnology, Jagiellonian University, Gronostajowa 7, 30-387 Kraków, Poland

**Keywords:** *Candida albicans*, candidiasis, amphotericin B, caspofungin, fluconazole, host response

## Abstract

The prevalence of diseases caused by pathogenic fungi of the *Candida* genus is currently a significant problem, particularly due to the emerging antifungal drug resistance and increasing number of immunocompromised individuals susceptible to opportunistic infections. Recently, it has been shown that fungal extracellular vesicles (EVs) – nanometre-sized structures of cellular origin, equipped with varied cargo enclosed by lipid bilayer – may play a vital role in the response of pathogen cells to antifungal treatment. In this work, we demonstrated that *Candida albicans* yeast cells grown at the subinhibitory concentrations of three commonly used antifungal drugs – amphotericin B, fluconazole and caspofungin – released a greater number of EVs than fungal cells grown under drug-free conditions. Moreover, these EVs exhibited some variability in size and protein composition, yet they consistently induced the production of the pro-inflammatory cytokine IL-8 by THP-1 macrophage-like cells at levels comparable to control EVs. In studies using the invertebrate model organism *Galleria mellonella*, EVs released by cells exposed to antifungals did not cause a significant increase in larval mortality, similar to control EVs, although they triggered a remarkably lower activation of phenol oxidase in larval haemolymph. In addition, EVs produced in the presence of caspofungin interacted more noticeably with the membrane of U-937 pro-monocytic cells as indicated by measurements of zeta potential changes. Furthermore, tested EVs contributed to increased tolerance of *C. albicans* cells to the antifungal drugs. These observations underscore the unmissable role of EVs in the response of pathogen cells to antifungal treatment and highlight the importance of understanding EV functionalities in antifungal resistance.

## Data availability

The datasets generated during the current study are available in the Cracow Open Research Data Repository, https://doi.org/10.57903/UJ/ZNAONT. The mass spectrometry proteomic data have been deposited to the ProteomeXchange Consortium via the PRIDE partner repository with the dataset identifier PXD054683.

## Introduction

*Candida albicans*, an ascomycetous yeast and an important component of the human microbiome, still remains also a critical and widely distributed fungal pathogen, which poses a significant threat to human health and life, particularly in immunocompromised individuals. The diverse types of infections caused by *C. albicans* range from harmless but troublesome superficial candidiases of the skin and mucous membranes to severe life-threatening systemic infections with a high mortality rate [1,2]. The currently available arsenal of antifungal drugs applied to treat infections caused by yeasts from the *Candida* genus still includes only a few major groups of substances with a restricted mechanism of action, primarily targeting the major components of the fungal cell membrane or cell wall (ergosterol and *β*-1,3-glucan, respectively), although new therapeutic approaches are still intensively researched [3,6].

One group of commonly used antifungal drugs are highly lipophilic macrolides – polyenes [i.e. nystatin, natamycin and amphotericin B (AMB)] – that interact with the ergosterol in the fungal plasma membrane causing the formation of transmembrane channels and resulting in the disruption of cellular ionic and oxidative balance and thus cellular stability and functionality [7]. The next group of drugs used for the treatment of candidiasis are heterocyclic compounds – azoles, including triazoles applied in invasive fungal infections [i.e. fluconazole (FLU), itraconazole, voriconazole, posaconazole and isavuconazole]. The mechanism of their action is based on the inhibition of a cytochrome P450 enzyme encoded by the *ERG11* gene (lanosterol 14*α*-demethylase) involved in the biosynthesis of ergosterol from lanosterol, resulting in the production of toxic 14-*α*-methylsterols and deterioration of the stability of the fungal cell membrane [8]. Another important group of antifungals are semisynthetic cyclic lipopeptides – echinocandins, with caspofungin (CASP), micafungin and anidulafungin as representatives [9]. They act through the inhibition of transmembrane heteromeric glycosyltransferase *β*-1,3-glucan synthase and particularly by their binding to the Fks1 subunit of this enzyme encoded by the *FKS1*, *FKS2* and *FKS3* genes. The result of the action of echinocandins is a disturbance of the proper formation of the fungal cell wall [10]. The occurrence of resistance and/or tolerance to antifungal agents, which is currently an increasing challenge in clinical practice, is driven by multiple underlying processes, including loss-of-function mutations, variations in the abundance or availability of drug targets, their replacement with alternate molecules, the increased expression and activity of drug efflux pumps, activation of stress response mechanisms and formation of biofilms [11,12].

In the last few years, the production of extracellular vesicles (EVs) – structures of cellular origin that contain a variety of molecules enclosed by a lipid bilayer and involved in several aspects of fungal biology – has drawn significant attention in the broad context of pathogenicity of fungal infections and challenges in antimicrobial therapy [13,15]. Recent studies indicate that fungal EVs might be implicated in the decreased susceptibility of *Candida* biofilms to antifungal treatment [16,20]. In general, the formation of biofilms as complex and structured communities, characterized by an extensive extracellular matrix and formed by microbial cells found in different morphological forms or physiological states, is considered as strongly associated with the development of tolerance to antifungal agents [21], and the production of EVs might play an important role in this phenomenon. Such effect has already been demonstrated in the case of FLU action on *C. albicans*, *Candida tropicalis*, *Candida parapsilosis*, *Candida glabrata* and *Candida auris* biofilms, as well as the action of CASP on the biofilms of the first two aforementioned *Candida* species [16,20]. The involvement of EVs in decreased biofilm susceptibility to FLU has been elucidated by the enzymatic activity of various proteins carried by vesicles and their contribution to the formation of biofilm matrix glucans and mannans and drug sequestration [16,18].

Beyond addressing biofilms, it has also been demonstrated for *C. auris* cells that the MICs for AMB were higher in the presence of EVs of the same species, compared with their absence or presence of *C. albicans* EVs. Particular *C. auris* enzymes found in vesicles, including alcohol dehydrogenase and proteins similar to *C. albicans* Mp65 and Xog1, have been indicated as involved in the increased resistance of *C. auris* to the investigated antifungal drug [22]. Additionally, for another important fungal pathogen – *Cryptococcus neoformans –* it has been shown that resistance to FLU was coregulated with the production of EVs, as fungal cells might have modified vesicles’ release capability as an initial strategy to combat membrane stress caused by the action of antifungal drug [23]. Hence, the amount of produced EVs was lower at subinhibitory concentrations of FLU, and the acquisition of resistance to the drug by diverse strains also resulted in a similar effect [23].

Thus, the findings presented to date suggest that the role of fungal EVs in cellular response to antifungal drugs is versatile, albeit complex. Furthermore, one could assume that fungal cells exposed to a subinhibitory concentration of the drug retained their ability to produce EVs, and these EVs may have different properties compared with those produced by cells that were not exposed to the drug. This underscores the need for further detailed investigation, not only with regard to the contribution of EVs to drug tolerance but also to their subsequent downstream interactions and fungal virulence. Therefore, the aim of this study was to verify whether and how *C. albicans* cells grown in the presence of subinhibitory concentrations of the three most commonly applied antifungal drugs – AMB, FLU and CASP – respond by modulating the vesicle production capacity and altering the properties of released EVs. A comprehensive understanding of the mechanisms of EV involvement in the response of fungal cells to drugs could be essential in guiding the implementation of effective antifungal therapies, as evidenced by the research presented herein, demonstrating that fungal cells exposed to drugs exhibit an increased production of EVs, which may reciprocally contribute to improved tolerance of fungi to the treatment, as well as influence host immune mechanisms by altering cytokine production and modulating immune responses.

## Methods

### Fungal strains, growth conditions and EV isolation

*C. albicans* strain 3147 (ATCC 10231) was purchased from the American Type Culture Collection (Manassas, VA, USA). Yeasts were routinely cultured in 20 ml of YPD (yeast extract peptone dextrose) liquid medium (pH 6.0; 1% yeast extract, 2% soybean peptone and 2% glucose; Sigma-Aldrich, St. Louis, MO, USA) for 18 h at 30 °C with rotary shaking (170 r.p.m.) in MaxQ 4000 orbital shaker (Thermo Fisher Scientific, Waltham, MA, USA). After this time, fungal cells were harvested by centrifugation at 3,000 ***g*** for 3 min and washed three times with Dulbecco’s PBS (DPBS), pH 7.5 (Biowest, Nuaillé, France). The number of fungal cells per millilitre was assessed by the measurement of the OD of the cell suspension at 600 nm using a Shimadzu UVmini-1240 spectrophotometer (Shimadzu, Kyoto, Japan).

For the isolation of EVs from solid cultures, the previously described procedure was used [24,25], with some modifications. Briefly, 1×10^8^ *C. albicans* cells were inoculated onto the YPD agar plate, which had previously been soaked with a solution (100 µl) of selected antifungal drug at a concentration of 0.5 µg ml^−1^ for AMB (Biowest), 0.1 µg ml^−1^ for FLU or 0.005 µg ml^−1^ for CASP (both from Sigma-Aldrich). In addition, the cultures of *C. albicans* on Petri dishes with YPD agar without any antimycotic drug added were prepared similarly, with 1×10^8^ yeast cells inoculated per one plate. Fungal cultures on solid media were incubated for 24 h at 37 °C and ambient air CO_2_ concentration in the incubator MaxQ 6000 (Thermo Fisher Scientific). Then, *C. albicans* cells were collected from the agar surface with the sterile loop, suspended in 1 ml of DPBS and gently mixed. After 5 min, the centrifugation was performed at 4,000 ***g*** for 15 min at 4 °C in two cycles, first to discard yeast cells and second to remove cell remnants from the supernatants, which were then additionally filtered with Ultrafree-CL Centrifugal Filters with a pore size of 0.65 µm (Merck, Darmstadt, Germany). Additionally, pellets containing centrifuged yeast cells were suspended in 1 ml of DPBS and aliquots were taken from the suspension and serially diluted tenfold in sterile DPBS, and 10 µl of the cell suspensions was plated onto YPD agar and incubated for 24 h at 30 °C to then count the c.f.u. in order to evaluate viable *C. albicans* cells. Two biological replicates with three technical replicates in each were performed, and the averaged results are presented.

To obtain EVs from the filtered supernatants, the ultracentrifugation in an Optima™ LE-80K Ultracentrifuge (Beckman Coulter, Brea, CA, USA) was performed at 144,000 ***g*** (*k* factor 112) for 1 h at 4 °C using polycarbonate thick-wall centrifuge tubes (13×64 mm) with 13 mm diameter Delrin tube adapters and a fixed-angle type 60 Ti Rotor, all sterilized with 70% ethanol and ultraviolet light before use. Pellets containing EVs were suspended in sterile DPBS and aliquoted. Five microlitres of the EV-containing sample after each isolation were plated on YPD agar plates for 48 h at 37 °C to check for microbial contamination. Then, EV aliquots have been stored at −80 °C for further use.

To test the impact of EVs produced in the presence of selected antifungal drug on the viability of *C. albicans*, to 1×10^4^ yeast cells in 100 µl of DPBS, pH 7.5, 1×10^9^ of each type of isolated fungal vesicles were added and incubated for 4 h at 37 °C in Eppendorf tubes. Then, cell suspensions were tenfold diluted in sterile DPBS and aliquots of 10 µl were applied to YPD agar plates and incubated for 24 h at 30 °C, and then, c.f.u. were counted. In addition, into the wells of sterile, clear, U-shaped-bottom 96-well microplate (Greiner Bio-One, Kremsmünster, Austria), 1×10^4^ yeast cells in 200 µl of YPD broth were inoculated with 1×10^9^ of EVs and cultured for 18 h at 30 °C in the Synergy H1 microplate reader (BioTek Instruments, Winooski, VT, USA) with continuous linear shaking at frequency 567 c.p.m. (3 mm). The measurements of OD at 600 nm have been taken every 30 min to monitor the fungal growth. The OD measurement value obtained for the medium alone was subtracted from the other values. In each case, fungal cells without added vesicles served as a control for undisturbed *C. albicans* growth. Two biological replicates with three technical replicates in each were performed, and the representative result is presented.

### Characterization of fungal EVs

The characterization of *C. albicans* EVs released by cells cultured in the presence of antimycotic drugs was performed by transmission electron microscopy (TEM), nanoparticle tracking analysis (NTA) and measurement of lipid and protein concentrations similar to previously described [19,26].

Visualization of EVs collected from different growth conditions was performed with a TEM JEOL JEM-2100 HT (JEOL, Tokyo, Japan), after negative staining using 2% uranyl acetate of EV preparations collected on formvar coated, 300 mesh copper grids (Chemapol, Prague, Czech Republic).

The analysis of the size and concentration of obtained EVs was performed using the NTA with a NanoSight NS300 system with camera type sCOS, laser Blue488 and NTA software v.3.4 (Malvern Instruments, Malvern, UK). The measurements were conducted at 25 °C in PBS filtered through a 0.22 µm filter (Lonza, Basel, Switzerland). For each sample from two EV isolations, the recording was repeated three times for 60 s with a camera level of 13 and a detection threshold parameter of 2.

The measurements of the concentration of phospholipids and proteins in EV-containing samples were performed with microplate assays in two or three biological replicates for every type of sample, respectively, using a Synergy H1 microplate reader (BioTek Instruments). The *ο*-phthalaldehyde assay was applied to determine the protein concentration by the measurement of the fluorescence intensity with excitation and emission at 340 and 455 nm, respectively. The Phospholipid Assay Kit (MAK122, Sigma-Aldrich) was used to measure the phospholipid concentration strictly in accordance with the manufacturer’s instructions.

### Proteomic identification of EV proteins

The proteins from *C. albicans* vesicles were identified with liquid chromatography-coupled MS-MS using a similar procedure as previously described [19,25] with some minor modifications. The total amount of EVs corresponding to 11 µg of protein was prepared in 100 µl of 100 mM Tris-HCl buffer, pH 7.6, with 1% sodium dodecyl sulphate and sonicated in four cycles of 30 s with UP50H Compact Lab Homogenizer, and the parameters are as follows: 30 kHz, amplitude 80%, cycle 0.5, and 50 W (Hielscher Ultrasonics, Teltow, Germany). Further procedures were implemented strictly according to the protocols described by Surman *et al*. [27] with the use of paramagnetic bead technology based on single-pot solid phase enhanced sample preparation [28]. The obtained peptides were identified by MS-MS analysis with the use of the UltiMate 3000 RSLCnano System coupled with Q Exactive mass spectrometer (Thermo Fisher Scientific) and a DPV-550 Digital PicoView nanospray source (New Objective, Woburn, MA, USA). Peptides were first separated on a trap column (Acclaim PepMap 100 C18, 75 µm×20 mm, 3 µm particle, Thermo Fisher Scientific) and then on an analytical column (Acclaim PepMap RSLC C18, 75 µm×500 mm, 2 µm particle, Thermo Fisher Scientific). The obtained RAW files were processed with the Proteome Discoverer platform (v.1.4, Thermo Fisher Scientific) and continued to further search with an in-house MASCOT search engine (v.2.5.1, Matrix Science, London, UK). The Swiss-Prot protein sequence database was used with the following taxonomy restrictions: fungi (number of sequences: 568,002; number of sequences after taxonomy: 35,922). The following parameters were applied: fixed modification – cysteine carbamidomethylation; variable modifications – methionine oxidation; precursor mass tolerance – 10 p.p.m.; and fragment mass tolerance – 20 mmu. Target Decoy PSM Validator was used with the maximum false discovery rate of 0.01. After peptide identification, the normalized spectral abundance factors (NSAFs) have been calculated for each EV-containing sample separately [29,31] by dividing the spectral number (SpC) of each identified protein by its length (L, number of aa), and this value was then normalized by dividing by the sum of all SpC/L for all proteins identified in the sample and listed in Table S1 (available in the online Supplementary Material). The MS proteomic data have been deposited to the ProteomeXchange Consortium via the PRIDE partner repository [32] with the dataset identifier PXD054683.

### Effect on tolerance to antifungal drugs

The influence of fungal EVs on *C. albicans* tolerance to antifungal drugs was tested analogously as described previously [20] and performed in two independent biological replicates with three technical replicates each, while the representative results are presented. *C. albicans* cells (1×10^5^) in 100 µl of RPMI 1640 medium were placed into the wells of a Corning 96-well black/clear flat bottom polystyrene high bind microplate (Corning Inc., Corning, NY, USA) and incubated for 90 min at 37 °C in an atmosphere of 5% CO_2_ and 95% humidity. Then, unattached cells were removed by washing with 200 µl of DPBS, and to the wells, 200 µl of RPMI 1640 medium with or without 1×10^9^ of each type of tested fungal EVs was added for a further 2 h under the same conditions. After this time, the antifungals were added to the wells to a final concentration of 0.5 µg ml^−1^ for AMB, 0.1 µg ml^−1^ for FLU or 0.005 µg ml^−1^ for CASP and incubated for 24 h. Then, formed biofilms were washed three times with 200 µl of DPBS, and the OD at 600 nm was measured in each well with the nine-point area scan reading method using a Synergy H1 microplate reader. Cells cultured only in RPMI medium constituted a control biofilm, not subjected to any additional factors.

### Human cell lines

The human pro-monocytic cell line U-937 (human histiocytic lymphoma cell line purchased from ATCC) was cultured in suspension in RPMI 1640 medium (Biowest) containing 5% heat-inactivated FBS (Thermo Fisher Scientific) and 0.01% penicillin-streptomycin (Biowest) in the atmosphere of 5% CO_2_ at 37 °C and 90% humidity.

The human monocytic cell line THP-1 (Sigma-Aldrich) was cultured in RPMI 1640 medium supplemented with 10% FBS, 100 U ml^−1^ penicillin and 100 μg ml^−1^ streptomycin at 37 °C in the atmosphere of 5% CO_2_ and 95% humidity. To differentiate monocytes to macrophage-like cells, 5×10^5^ cells per well in a 24-well plate were seeded in RPMI 1640 medium supplemented with 5% FBS, 100 U ml^−1^ penicillin and 100 μg ml^−1^ streptomycin with the addition of 10 ng ml^−1^ of phorbol 12-myristate 13-acetate (PMA) (Sigma-Aldrich) for 48 h, with medium exchange after 24 h. After differentiation to adherent cells, the medium was replaced with medium without PMA, but with 5% FBS, 100 U ml^−1^ penicillin and 100 μg ml^−1^ streptomycin for 3 h under the same conditions.

### The measurement of zeta potential

The measurements of the zeta potential of EVs, liposomes and cells were performed with a dynamic light scattering technique and Malvern Zetasizer ZS apparatus (Malvern) with disposable folded capillary cells (Malvern). The mobility values were converted to zeta potentials using the Smoluchowski equation. The analysis of interactions of EVs with liposomes modelling the composition of the human cell membrane and with human cells U-937 as a whole was performed. Measurements with liposomes were performed in one biological replicate and with cells in three biological replicates. The zeta potentials of the liposomes or human cells in suspension (5×10^5^ cells ml^−1^) after interaction with fungal vesicles were measured using the method outlined by Bondar *et al*. [33]. The procedure was completed as described in detail previously [24], with the following composition of the membrane of liposomes: 30% 1,2-dipalmitoyl-sn-glycero-3-phosphocholine, 33% 1,2-dioleoyl-sn-glycero-3-phosphoethanolamine, 24% 1,2-dioleoyl-sn-glycero-3-phosphocholine, 13% 1,2-dipalmitoyl-sn-glycero-3-phospho-l-serine (Avanti Polar Lipids Inc.) and 29% cholesterol (Sigma-Aldrich). Prior to the experiments, U-937 cells were combined with a 0.9% NaCl solution, centrifuged at 1,000 ***g*** for 5 min, and the supernatant was removed. Each of the four types of EVs with a final protein concentration of 12 µg ml^−1^ in PBS at pH 7.4 was directly added to the liposomes or cells. After incubation for 2 min, the zeta potential was recorded. Each measurement was conducted a minimum of five times.

### Production of cytokines by THP-1 macrophage-like cells in response to EVs

THP-1 macrophage-like cells were incubated with *C. albicans* EVs in 300 µl of RPMI 1640 medium in a 24-well microplate in the final EV-to-cell ratio of 10^5^ to 1 for 24 h at 37 °C in the atmosphere of 5% CO_2_ and 95% humidity. Then, supernatants were collected from above the cell monolayer, and cell debris was removed from supernatants by centrifugation for 5 min at 1,000 r.p.m. The levels of IL-8 and IL-10 in supernatants were determined using BD OptEIA™ kits, Human IL-8 ELISA Set and Human IL-10 ELISA Set (BD Biosciences, Franklin Lakes, NJ, USA), respectively, strictly following the manufacturer’s instructions. Unstimulated cells served as a control. Cells incubated with 100 ng ml^−1^ LPS (Sigma-Aldrich) served as a positive control. Three independent biological replicates were performed, and the representative results are presented.

The cells that remained after the collection of the supernatants were tested with the XTT (sodium 3′-[1-(phenylaminocarbonyl)-3,4-tetrazolium]-bis (4-methoxy6-nitro) benzene sulfonic acid hydrate) reduction assay (Thermo Fisher Scientific) and Cytotoxicity Detection Kit^PLUS^ (Roche, Basel, Switzerland) to assess their metabolic activity and the cellular damage and lactate dehydrogenase (LDH) release, respectively, as described previously in detail [24]. Briefly, the XTT (Thermo Fisher Scientific) test was performed, after the adhered cells were washed twice with 300 µl DPBS (pH 7.5), and then, 200 µl of RPMI 1640 medium without phenol red (Biowest) and 100 µl of XTT reagent (containing XTT at a final concentration of 1 mg ml^−1^) and *N*-methyl dibenzopyrazine methyl sulphate at a final concentration of 5 µg ml^−1^ (Sigma) were added. Cells were incubated for 1 h at 37 °C in the atmosphere of 5% CO_2_.

To determine the LDH release after incubation of THP-1 cells with EVs, 50 µl of the RPMI 1640 medium without phenol red (Biowest) and 50 µl of a mixed reagent (250 µl of reconstituted catalyst with 11.25 ml dye solution) were added. For positive control, 5 µl of lysis solution was added to the cells. After incubation in the dark for 15 min at 37 °C in the atmosphere of 5% CO_2_, the reaction was stopped by adding 25 µl of stop solution. After transferring the supernatants to new 96-well microplates (Sarstedt, Nümbrecht, Germany), the absorbance measurement was performed at 450 nm (XTT assay) or 490 nm (LDH assay) using a Synergy H1 microplate reader.

### The survival of *Galleria mellonella* larvae and determination of phenol oxidase activity in haemolymph

The *in vivo* effects of *C. albicans* EVs produced by fungi in the presence of antimycotic drugs were examined using the *G. mellonella* larvae model. For each EV sample, a separate group consisting of ten randomly selected larvae was inoculated in the last left proleg with 10 µl of the solution containing 10^8^ of fungal EVs in sterile DPBS using a 10 µl Hamilton syringe (Merck). An additional group of ten larvae was inoculated with 10 µl of DPBS as a control. Then, *G. mellonella* larvae were incubated at 37 °C for 7 days in Petri dishes, and every 24 h, the number of dead individuals was documented. Death was assessed by the dark colouration of the larva and the absence of movement after physical stimulation. Three independent biological replicates were performed, and the averaged results are presented.

For the measurement of phenol oxidase activity, the haemolymph was collected from larvae by puncturing the head with a sterile needle 24 h after EV injection executed as described above. The experiment was performed in at least three biological replicates with three larvae in each group. The enzyme activity was assayed as described by Sulek *et al*. [34,35]. Briefly, 2 µl of haemolymph from each larva was transferred immediately after collection to 20 µl of 50 mM Tris-HCl buffer with 150 mM NaCl and 5 mM CaCl_2_, pH 7.4, and incubated for 20 min at room temperature in a 96-well microplate (Sarstedt). Then, to each well, 180 µl of 2 mM l-DOPA (Sigma) in 50 mM Na_2_HPO_4_ and 50 mM NaH_2_PO_4_, pH 6.5, was added and incubated further for 180 min at 37 °C. Measurements of absorbance at 490 nm were recorded during this time every 5 min with linear shaking for 1 s with frequency 567 c.p.m. (3 mm) using a Synergy H1 microplate reader. A sample with water instead of haemolymph served as a control, and the values obtained for control were subtracted from values measured for samples containing haemolymph.

### Statistical analysis

The data presented in the bar charts and scatter plots are mean values±sd or mean values±sem, as indicated in the legend provided beneath the respective figure. The analysis of the statistical significance was performed using an unpaired t-test, and the comparison of survival curves was performed with the log-rank Mantel–Cox test with GraphPad Prism software v.10.0.3 (GraphPad Software, La Jolla, CA, USA).

## Results

**Fig. 1.**
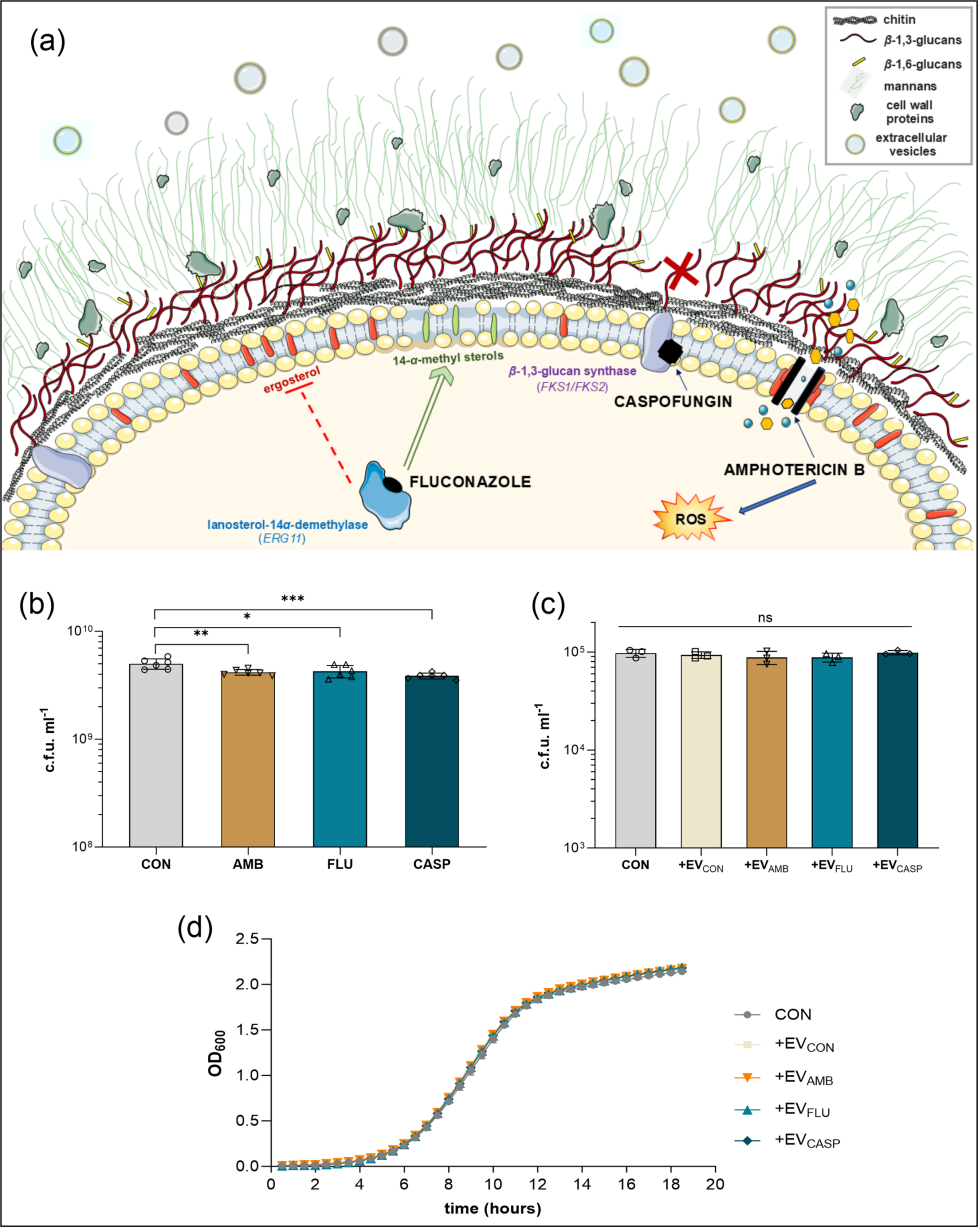
Release of EVs by *C. albicans* cells cultured in the presence of antifungal drugs, AMB, FLU or CASP. (**a**) Mechanism of action of selected antifungal drugs on cellular targets in *C. albicans* cells. The figure was partly generated using Servier Medical Art, provided by Servier, licensed under a Creative Commons Attribution 3.0 unported license. ROS, reactive oxygen species. (**b**) Viability of *C. albicans* cells collected from solid media for the subsequent isolation of specific types of EVs, presented as c.f.u. per 1 ml (CON, control conditions with *C. albicans* without antimycotics added). Two biological replicates with three technical replicates in each were performed, and the averaged results are presented. (**c**) The impact of EVs produced in the presence of antifungal drugs, on the viability of *C. albicans*, presented as c.f.u. per 1 ml (CON, control conditions with *C. albicans* without EVs added). (**d**) The impact of EVs produced in the presence of antifungal drugs, on the growth rate of *C. albicans* (CON, control conditions with *C. albicans* without EVs added). Two biological replicates with three technical replicates in each were performed, and the representative results are presented. The data presented on the bar charts are mean values±sd. The analysis of the statistical significance was performed using an unpaired t-test. The levels of statistical significance when compared with control (CON) are indicated as * for *P*<0.05, ** for *P*<0.01, *** for *P*<0.001 and ns for not significant.

Fungal EVs were isolated from YPD agar cultures of *C. albicans* ATCC 10231 yeasts grown in the presence of antimycotic drugs – AMB, FLU and CASP – or under control conditions (CON); hence, the obtained EVs are further designated as follows: EV_AMB_, EV_FLU_, EV_CASP_ or EV_CON_, respectively (Fig. 1a).

The drug concentrations used were significantly below the MICs and were selected based on studies previously performed in our group [36]. In addition, in the current study, the viability of *C. albicans* cells collected from a solid medium for EV isolation has been estimated by c.f.u. counting to verify the effect of selected antimycotics on the growth of fungal cells (Fig. 1b). The drug concentrations used did not significantly inhibit cell viability, but some slight reduction was still observed, confirming that the antimycotics used had an effect on the fungal cells. Importantly, as it has been additionally verified, EVs produced by *C. albicans* in the presence of these drugs did not inhibit the growth of fungal cells that have not previously been in contact with antifungals, as shown in Fig. 1(c, d).

**Fig. 2.**
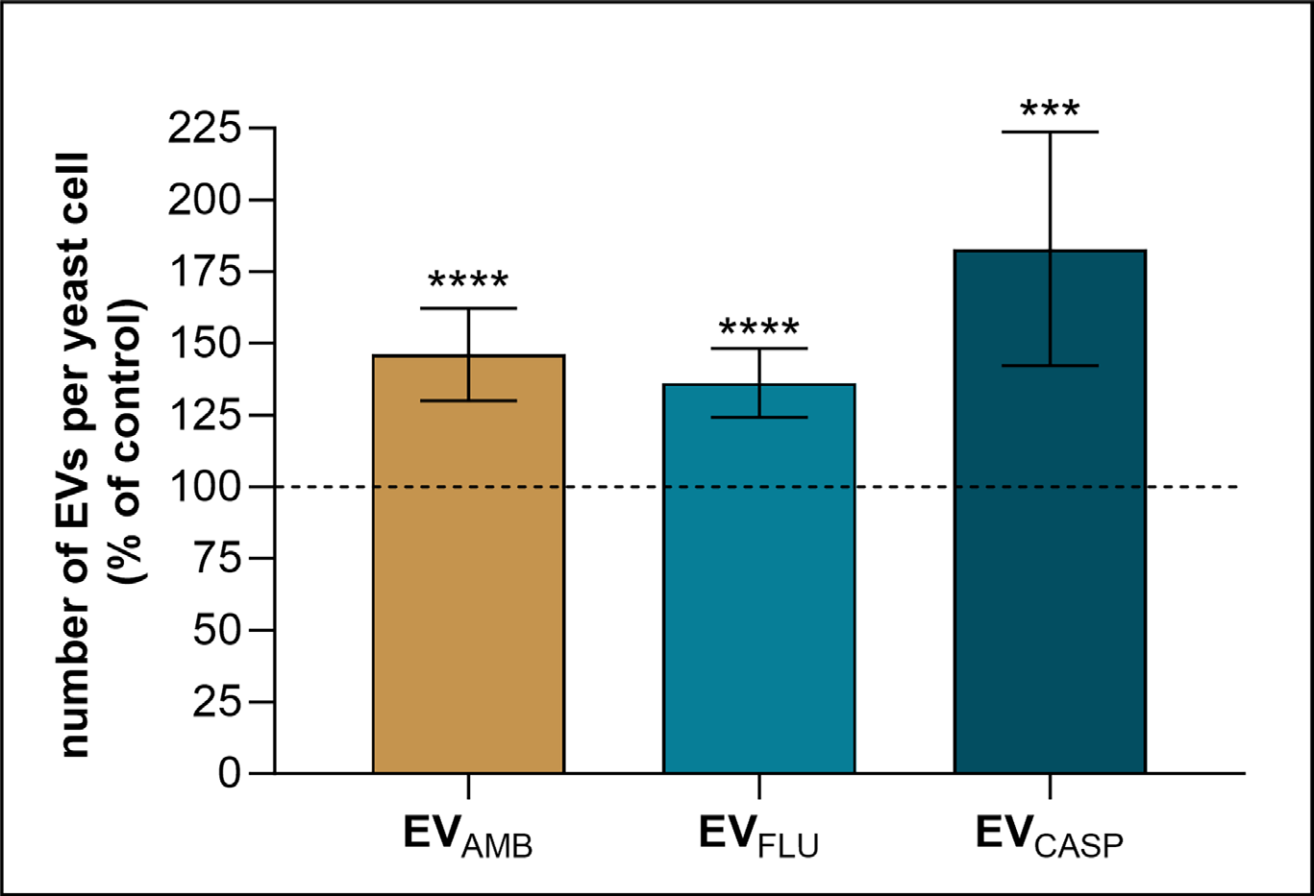
The change in EV production by fungal cells grown in the presence of antimycotic drugs, AMB, FLU or CASP. The number of EVs in obtained samples was measured with NTA, the number of viable yeast cells producing EVs was assessed by c.f.u. counting and the calculated ratios are presented as the per cent of the amount of control EVs. Two biological replicates with three technical replicates in each were performed, and the averaged results are presented. The data presented on the bar charts are mean values±sd. The analysis of the statistical significance was performed using an unpaired t-test. The levels of statistical significance when compared with EV_CON_ are indicated as *** for *P*<0.001 and **** for *P*<0.0001.

Furthermore, based on the measurement of EV concentration performed with NTA, the amounts of EVs produced by living cells from each culture condition were calculated (Fig. 2). Assuming the number of vesicles released by one viable yeast cell grown under control conditions without any antimycotic drug as 100%, it has been shown that the presence of each of the three tested antimycotics led to the increased production of EVs by yeasts. The greatest change was observed for cells cultured in the presence of CASP, with an increase of ~80%, while for AMB and FLU, the increase was ~40–45%.

**Fig. 3.**
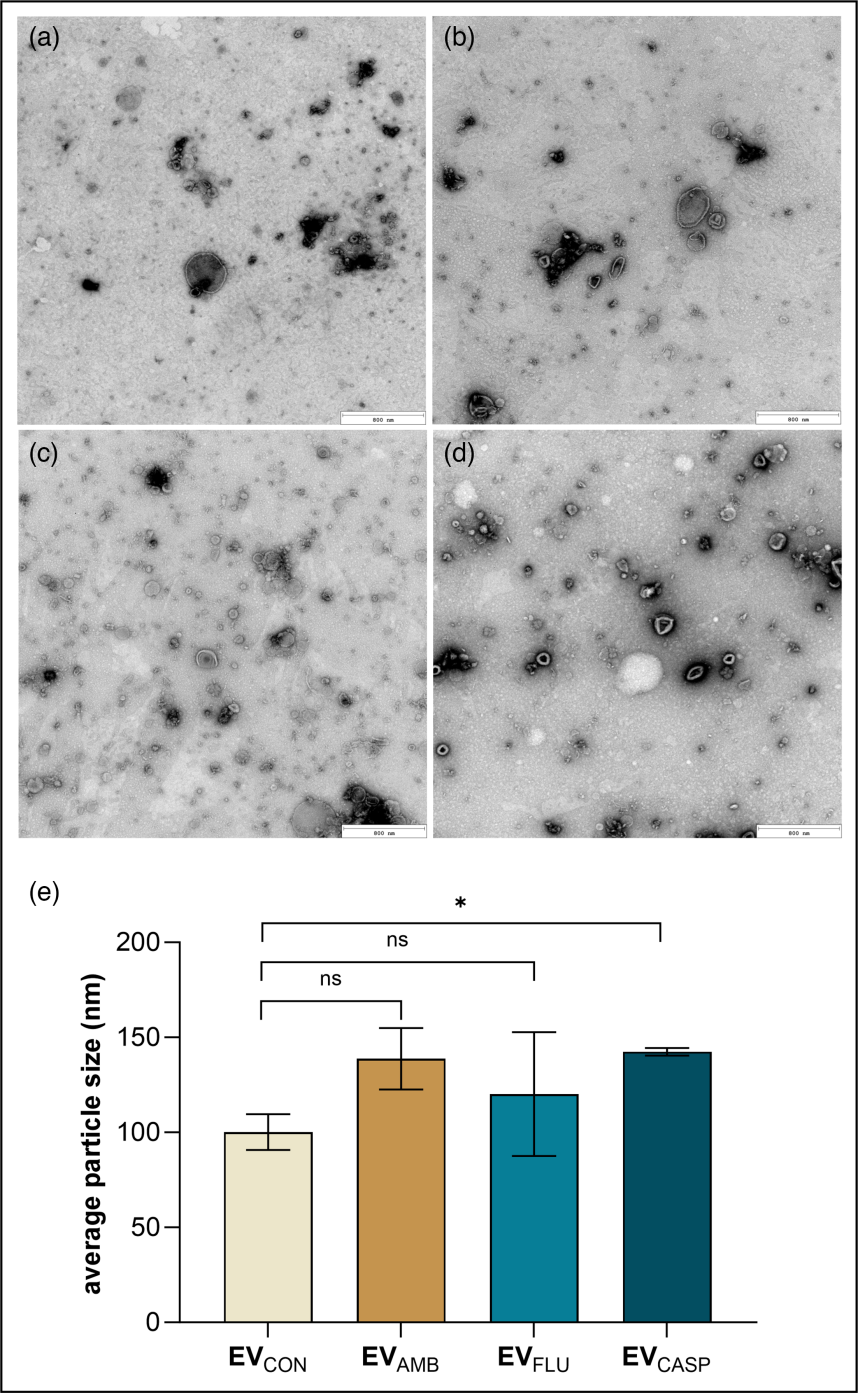
Characteristics of EVs produced by *C. albicans*. TEM photographs of EV_CON_ (**a**), EV_AMB_ (**b**), EV_FLU_ (**c**) and EV_CASP_ (**d**). Scale bar: 800 nm. (**e**) Average particle size (mode) determined by NTA from two histograms with the particle size distribution obtained after three measurements of each sample. The bar chart presents the mean values±sem. The level of statistical significance when compared with EV_CON_ is indicated as * for *P*<0.05 and ns for not significant.

The presence of lipid bilayered structures and their morphology were investigated using TEM visualization, and the EV size was measured using NTA (Fig. 3). The obtained results confirmed spherical, cup-shaped morphology of vesicles with the average size ranging from about 80 to 260 nm (detailed NTA parameters with histograms are presented in Fig. S1). The comparative analysis of the averaged mean values did not reveal statistically significant differences among the various EV types. However, considering the mode values for particle size, it was revealed that EV_CASP_ were statistically significantly larger compared with EV_CON_ (Fig. 3e). The average values were also higher for the other two types of EVs, but without statistical significance.

**Fig. 4.**
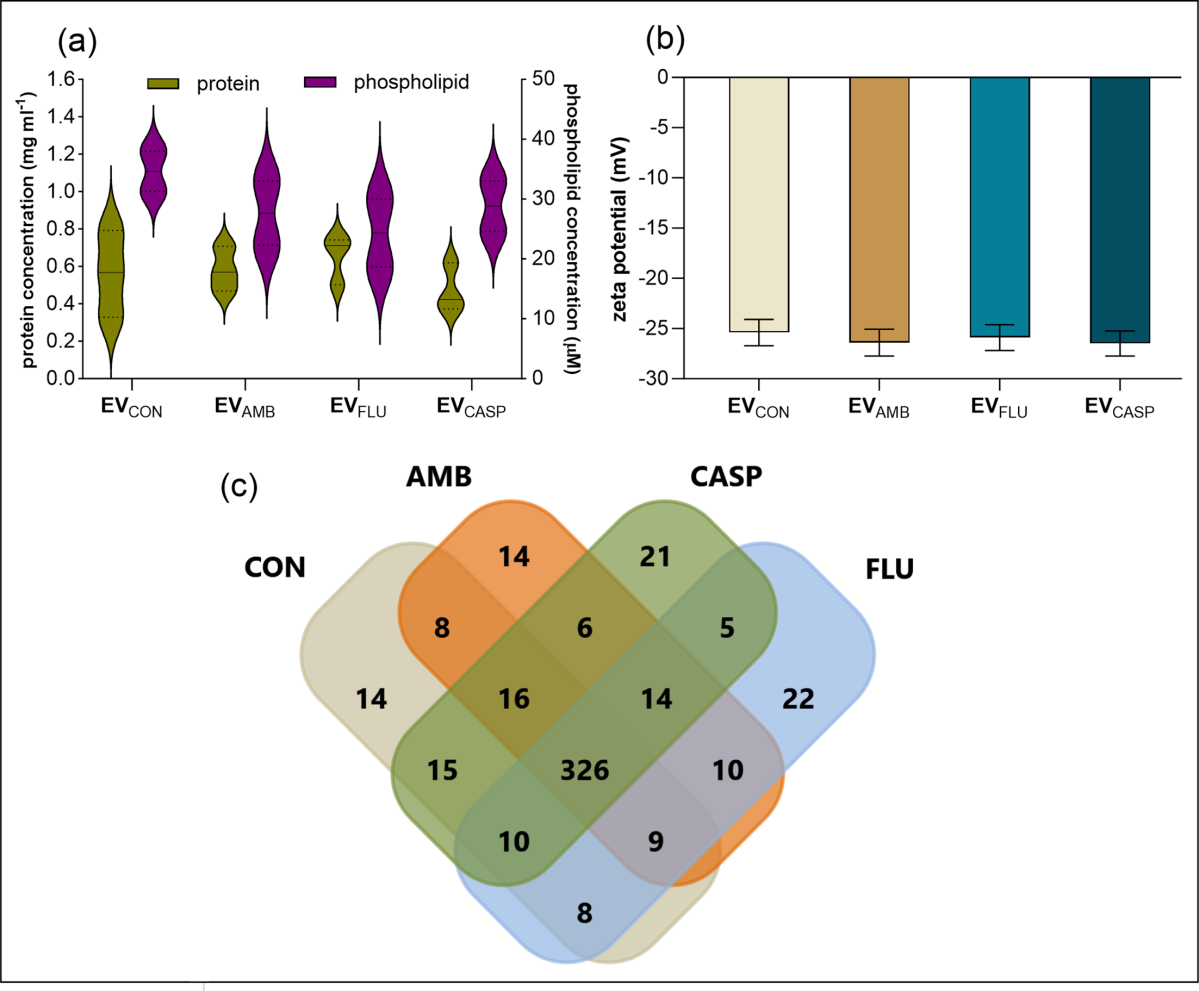
Composition of *C. albicans* EVs. (**a**) Protein and phospholipid concentration in isolated EVs. (**b**) The measurement of zeta potential of isolated EVs. The data presented in the violin plot are shown with the median and quartiles, while the data presented in the bar charts represent mean values±sd. The analysis of the statistical significance was performed using an unpaired t-test and demonstrated no significant differences. (**c**) Venn diagram of proteins identified in EVs produced by *C. albicans* cells cultured in the presence of antifungal drugs (CON, control conditions without antifungal drugs added). Created using the enrichment analysis tool FunRich v.3.1.3 (http://www.funrich.org) [37].

The measured protein and phospholipid concentrations for four investigated EV-containing samples confirmed the presence of these two major vesicle-building components, but there were no statistically significant differences in concentrations when comparing distinct types of EVs (Fig. 4a). In addition, only very minor and statistically insignificant differences were noted also for the zeta potential measurements of EVs (Fig. 4b). This demonstrates comparable properties of studied fungal EVs, regardless of their production in the presence of pharmacological agents.

The proteomic analysis of the content of the isolated EVs indicated the comparable number of proteins, which were identified for each type of *C. albicans* EVs produced in the presence of antimycotics and for EVs released in the control conditions (Table S1). The analysis of the cellular location of identified vesicular proteins using the FunRich tool [37] indicated a quite similar content of proteins originating from the cytoplasm, intracellular membranes, plasma membrane, cell wall, biofilm matrix or proteins directly assigned to EVs, for each analysed type of *C. albicans* EVs (Fig. S2). Additionally, the functional categorization of proteins was also comparable (Fig. S3). Most identified proteins (326) were common to all applied growth conditions, but EVs from each culture also had their own set of unique proteins (Fig. 4c and Tables S2 and S3). Interestingly, among the proteins identified in the tested sample in EV_FLU_ and EV_CASP_, but not in EV_CON_ and EV_AMB_, was the major facilitator superfamily multidrug transporter Flu1 responsible for fungal resistance to FLU [38]. However, it is important to note that further proteomic studies with increased sensitivity will enable the detection of more detailed differential profiles between particular types of EVs.

Several proteins involved in the maintenance and rearrangement of the cell membrane and cell wall were identified in EV-containing samples. Among others, in the cargo of each type of tested EVs, the presence of protein Sur7, chitin synthases 2 and 3 (Chs2 and Chs3), lanosterol 14*α*-demethylase (Erg11) and cell surface mannoprotein Mp65 was confirmed. Protein Sur7 was previously proposed as a proteinaceous marker for *C. albicans* EVs [39]; thus, its identification additionally confirms the presence of these structures in obtained samples. Moreover, there were some slight differences in the relative abundance of particular proteins in distinct types of EVs, assessed on the basis of calculated NSAFs. For lanosterol 14*α*-demethylase (Erg11), the increase of NSAF was shown for EV_FLU_, while for chitin synthase 2 (Chs2) and chitin synthase 3 (Chs3), NSAFs were higher in EV_CASP_ than in EV_CON_. Several proteins involved in glucan metabolism and synthesis were also present in all tested types of EVs, including 1,3-beta-glucanosyltransferase Pga4, glucan 1,3-beta-glucosidases (Exg1 and Bgl2), beta-glucan synthesis-associated proteins Kre6 and Skn1 and endo-1,3(4)-beta-glucanase 1 (Eng1). Similar to the previous studies, a number of cytoplasmic proteins were identified in all types of tested EVs including glyceraldehyde-3-phosphate dehydrogenase Tdh3, phosphoglycerate kinase Pgk1, enolase Eno1 and alcohol dehydrogenase Adh1 [20,26]. Importantly, the Hsp90 homologue has been identified in all types of EVs examined in this work, with slightly increased relative abundance in EV_CASP_, and this molecular chaperone is considered to be a central regulator of the network governing cellular stress responses in fungal cells [11].

**Fig. 5.**
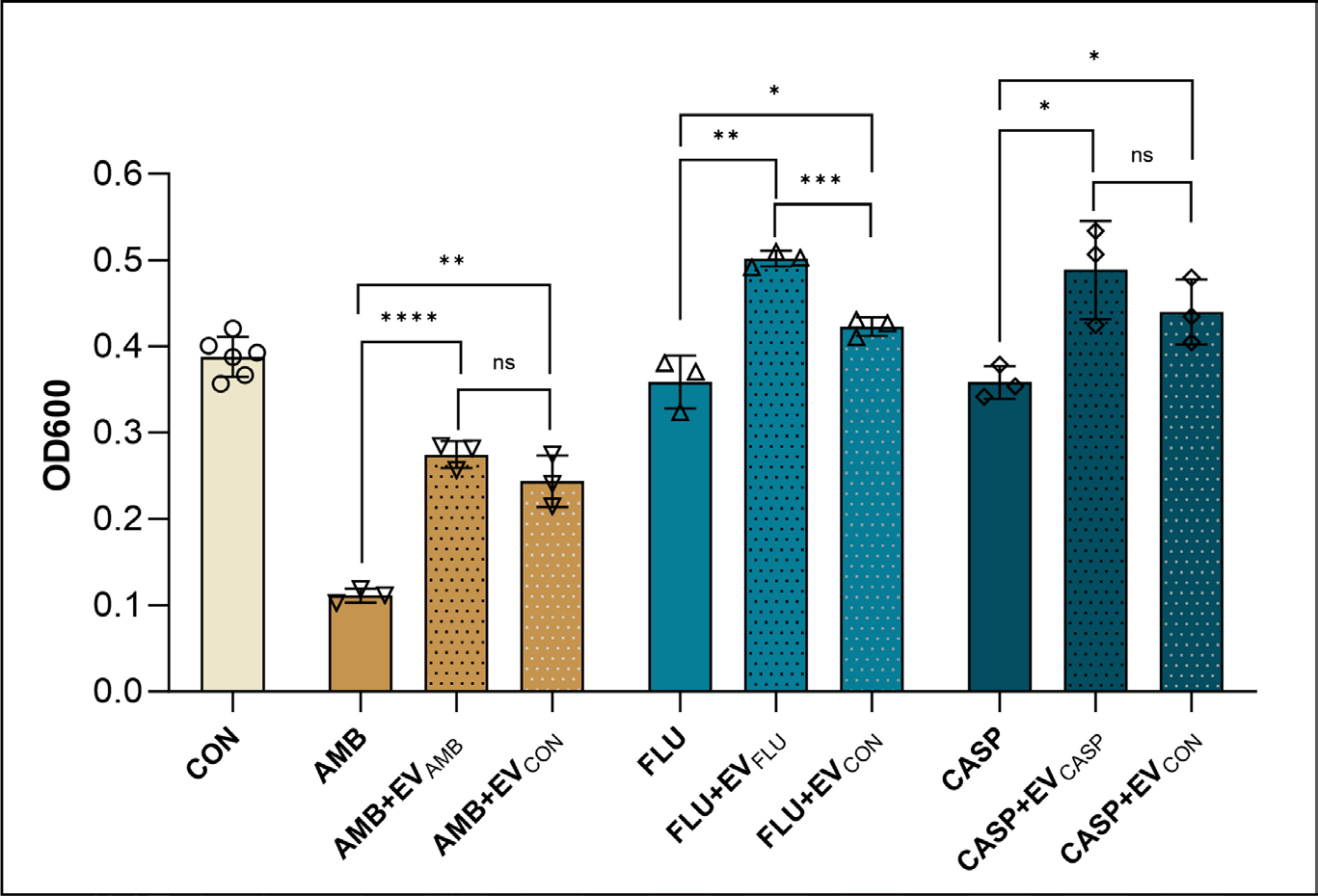
The impact of *C. albicans* EVs on tolerance to antifungals. Formation of *C. albicans* biofilm in the presence of the antifungal drugs, AMB, FLU and CASP and fungal EVs. The biofilm-forming capacity was examined by measuring the OD at 600 nm in the area scan mode. A representative result from two biological replicates is presented. The data presented on the bar charts are mean values±sd. The statistical analysis was performed using an unpaired t-test. The level of statistical significance is indicated as *P<0.05, **P<0.01, ***P<0.001, ***P<0.0001 and ns when not significant.

Referring to previous reports on the effect of vesicles on the increased tolerance of *C. albicans* cells in the biofilm to antifungal drugs [17,20], we tested whether such a protective effect is maintained in the case of vesicles produced in the presence of drugs. EVs derived from a control culture, as well as those obtained from a culture cultivated in the presence of a specific drug, were analysed for the cell response to the respective drug with regard to the ability to grow and form a biofilm (Fig. 5). In the case of all tested antifungals, the presence of EVs, both produced by the naïve *C. albicans* cells and those cultured in the presence of a particular drug, increased considerably the tolerance of biofilm-forming cells to the antifungal treatment, measured as the relative thickness of the biofilm and cell growth within the biofilm.

**Fig. 6.**
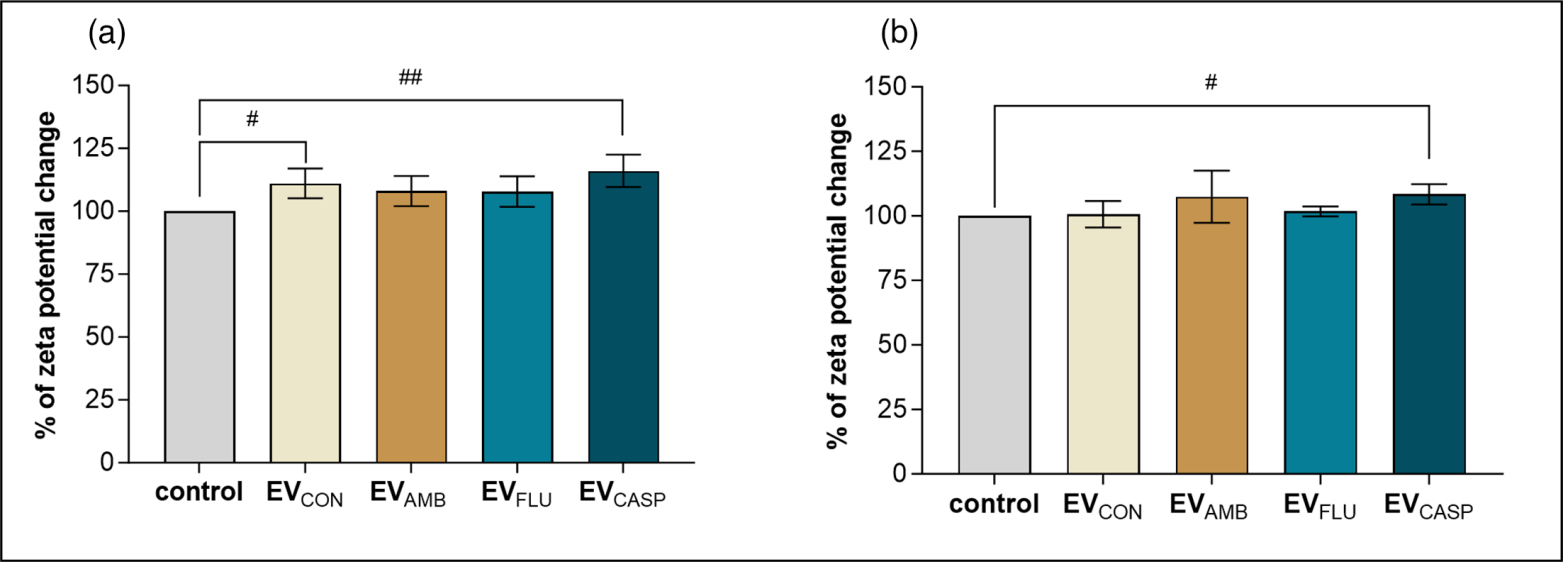
The changes in zeta potential of liposomes or U-937 cells in response to *C. albicans* EVs. Measurement of changes in zeta potential of (**a**) liposomes with a lipid bilayer modelled after the lipid composition of the cell membrane of U-937 cells and (**b**) U-937 cells as a whole during interactions with *C. albicans* EVs. Measurements with liposomes were performed in one biological replicate and with cells in three biological replicates. The data presented on the bar charts are mean values±sd. The statistical analysis was performed using an unpaired t-test. Only comparisons with statistical significance are indicated, and the level of statistical significance when compared with cells or liposomes without added EVs (control) is marked as # for *P*<0.05 and ## for *P*<0.01.

Subsequently, the impact of culturing fungal cells in the presence of antifungal drugs on the functional capacity of EVs produced by them to affect human cells was investigated. For this purpose, cell lines of pro-monocytic human cells and those differentiated into macrophage-like cells were selected, as well as their simplified model, namely, liposomes with a membrane modelling the membrane of human defence cells in terms of lipid composition, but without the human membrane proteins. Therefore, the initial stage involved measuring zeta potential changes using two models of interaction with EVs: first, liposomes with a lipid bilayer that mimics the lipid composition of the U-937 cell membrane, and second, whole U-937 cells (Fig. 6a, b).

Using the liposome model, some increase in their zeta potential was observed during contact with vesicles, with the greatest change after contact with EV_CASP_, comparable with the change for the sample treated with EV_CON_, whereas the values for liposomes treated with EV_AMB_ and EV_FLU_ were similar, both lower than for EV_CON_ and statistically not different from the control (Fig. 6a). Using U-937 cells as a model, even smaller changes in their zeta potential were noticed, and only for cells incubated with EV_CASP_ the increase was statistically significant compared with control, while the observed increase for cells treated with EV_AMB_ was not (Fig. 6b).

**Fig. 7.**
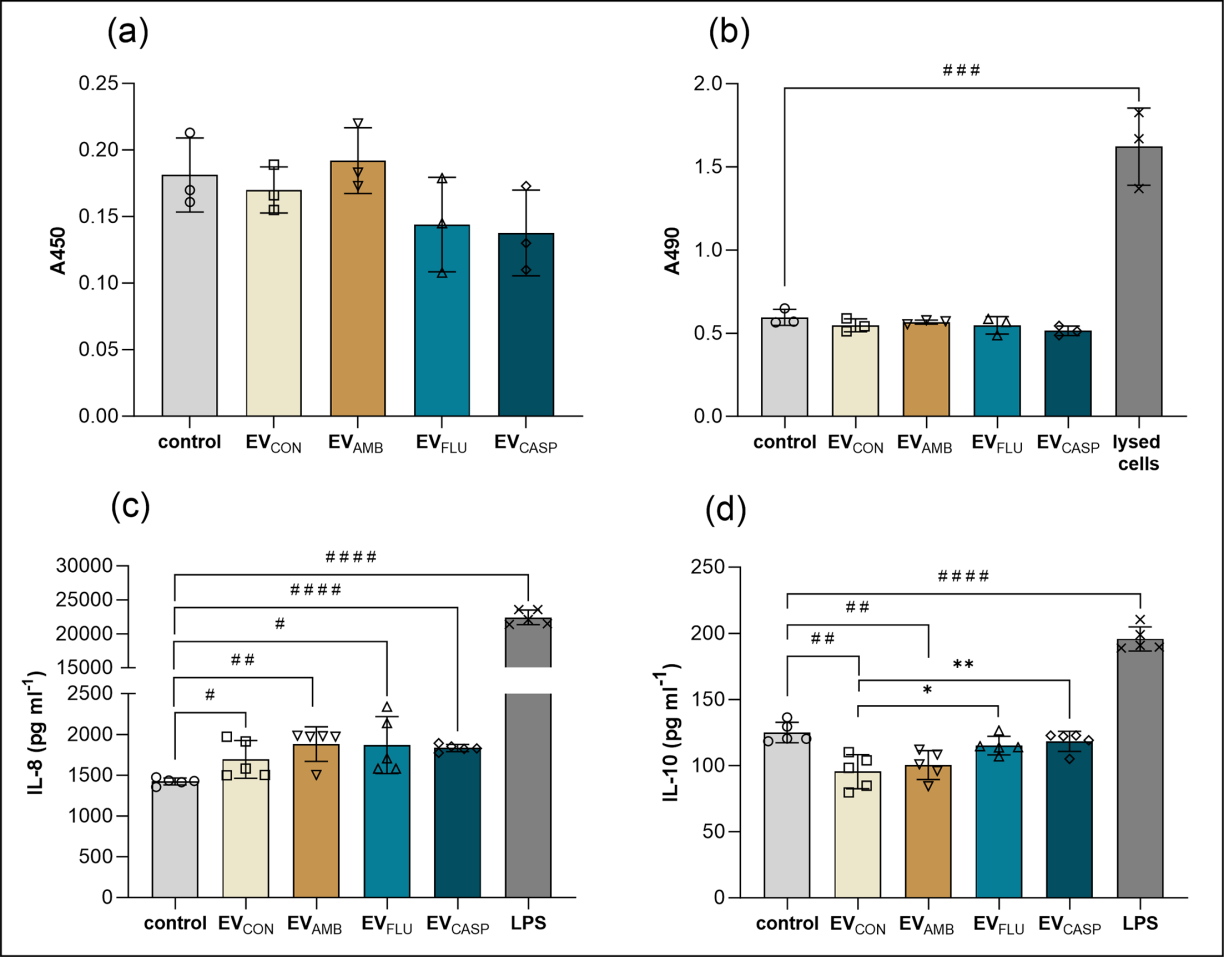
The THP-1 host cell response to *C. albicans* EVs. Analysis of (**a**) metabolic activity and (**b**) LDH release by THP-1 cells after 24 h of contact with fungal EVs. The release of IL-8 (**c**) and IL-10 (**d**) by THP-1 macrophage-like cells after 24 h of contact with fungal EVs. THP-1 cells without contact with EVs served as a control. The final concentration of LPS was 100 ng ml^−1^. Three independent biological replicates were performed, and representative results are presented. The data presented on the bar charts are mean values±sd. The statistical analysis was performed using an unpaired t-test, and the level of statistical significance when compared with cells without added EVs (control) is indicated as # for *P*<0.05, ## for *P*<0.01 and #### for *P*<0.0001 and when compared with EV_CON_ as * for *P*<0.05 and ** for *P*<0.01.

The response of THP-1 macrophage-like cells to the stimulation with EVs released by *C. albicans* in the presence or lack of antimycotic drugs was analysed by metabolic activity measurement using XTT assay, cytotoxicity measurement using LDH release assay and determination of levels of secreted cytokines IL-8 and IL-10. No statistically significant changes in the metabolic activity of THP-1 macrophage-like cells were noticed; however, a slight decrease after stimulation with EV_FLU_ and EV_CASP_ could be observed (Fig. 7a). Additionally, no changes in the release of LDH, informing about the cytotoxic effect of all tested types of EVs, were revealed (Fig. 7b). The level of IL-8 secretion, being a chemoattractant for other types of immune cells, not only monocytes and macrophages but also for neutrophils and T cells, increased after the stimulation of THP-1 cells with all four types of EVs, but without significant differences between them (Fig. 7c). Contrary to this, the decrease in the level of IL-10 was the greatest for EV_CON_ and EV_AMB_, while for EV_FLU_ and EV_CASP_, the result did not differ from the control, which consisted of cells not stimulated with EVs, though in relation to EV_CON_ and EV_AMB_ was statistically significantly higher (Fig. 7d).

**Fig. 8.**
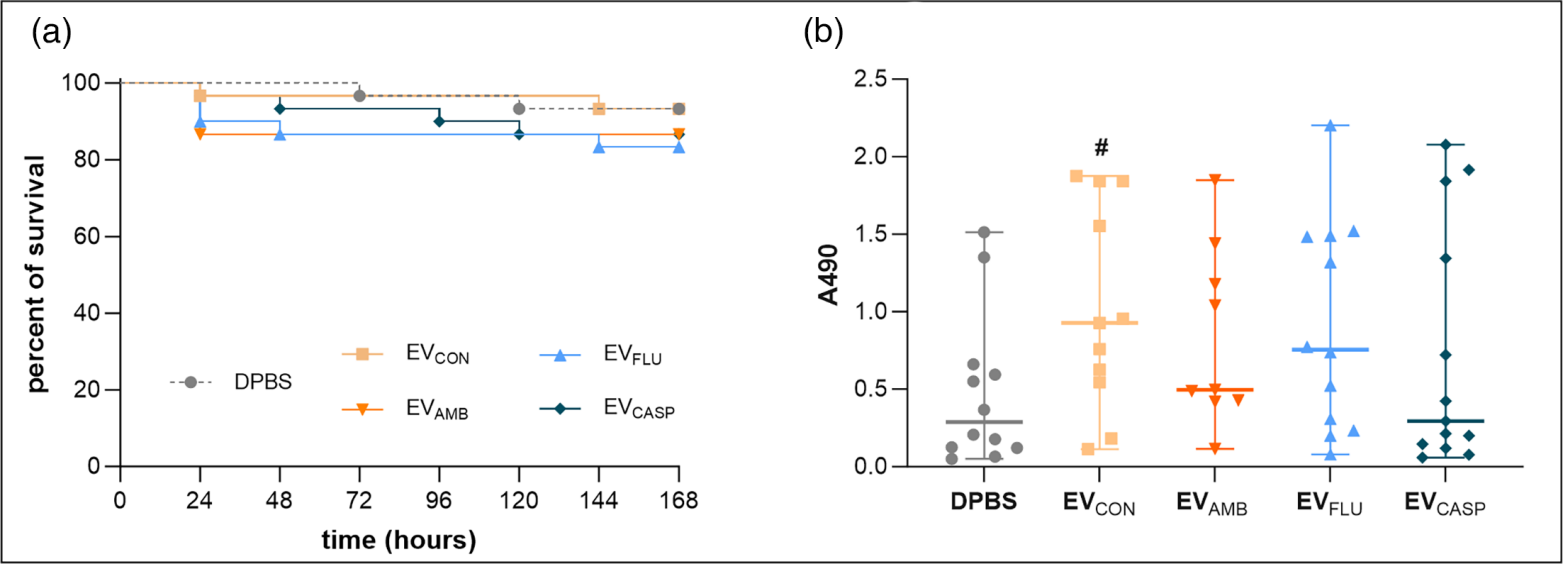
The impact of EVs on the survival of *G. mellonella* larvae (**a**) and activity of phenol oxidase in haemolymph (**b**). Larvae were injected with 10^8^ of *C. albicans* EVs in 10 µl of DPBS. Injection with DPBS served as a control. The average result of three independent biological replicates performed with ten individuals in each group is presented for survival analysis (**a**) and with three individuals for phenol oxidase activity measurement at 130 min of the enzymatic reaction (**b**). The data presented on the scatter plots are median values with ranges. The statistical analysis was performed using an unpaired t-test, and the statistical significance for comparison versus DPBS is marked with # for *P*<0.05.

To determine the *in vivo* effect of EVs on the host, the survival rate of *G. mellonella* larvae was analysed after injection of 10^8^ vesicles per individual (Fig. 8a). There was a minor, albeit statistically not significant, difference between the overall effect caused by three types of EVs released in the presence of antifungals and that of EV_CON_. For the latter, the survival rate was similar to that of the group after DPBS injection, causing negligible larvae mortality.

The activity of the phenol oxidase, which is an important enzyme for the invertebrate immune defence, was measured after collecting haemolymph from the larvae 24 h after EV injection. Interestingly, the activity of phenol oxidase in the haemolymph after injection of EV_CON_ was increased significantly when compared with DPBS-injected larvae, indicating the activation of larval immune response and enhanced capacity to address the challenge posed by the injection of EVs. At the same time, for EV_AMB_, EV_FLU_ and EV_CASP_, such considerable activation was not observed, and the median value for the group injected with the latter vesicles closely approximated that of the DPBS group, suggesting compromised immune function after exposure to these particular fungal structures (Fig. 8b).

## Discussion

In the era of the increasing resistance of fungal pathogens to drugs used in the treatment of *Candida* infections, it is critical to understand how the fungal cells react upon exposure to antifungal agents. Changes in gene expression, metabolic adaptation, production of effector proteins and a variety of enzymes and rearrangement of subcellular and cellular structures, including the cell wall and plasma membrane, are effects triggered by pathogens to reduce the efficacy of antifungals. Recent studies have indicated that the production of EVs may also contribute to fungal tolerance and resistance to treatment [17]. Therefore, in this work, we investigated how the presence of selected, commonly used antifungal drugs might affect the release of vesicles by fungi and modulate their subsequent action on the host, as the introduction of antifungal drugs to the culture during the growth of *C. albicans* cells may influence not only their viability but also the properties of released EVs.

In the current study, the concentration of added antifungal drugs was standardized for the culture of *C. albicans* ATCC 10231 to affect the viability of fungal cells, but to still observe fungal growth [36], similarly as reported for *C. auris* by Zamith-Miranda *et al*. [40]. At higher concentrations of antifungal agents, partial inhibition of fungal growth occurs, leading to alterations in the metabolic activity of *C. albicans* cells [36]. Such metabolic disruptions may further influence the rate of EV production, as vesicle release is dependent on adaptive cellular mechanisms in response to stimuli or stress associated with the host microenvironment or induced by exogenous agents introduced into the milieu [24,41, 42]. On the one hand, at high drug concentration, the number of viable cells capable of releasing EVs may decrease; however, remaining stressed but still alive cells could respond by generating a more diverse and abundant population of EVs, potentially as a result of drug-induced disruptions of the cell membrane or cell wall integrity and/or the activation of stress response signalling pathways [3,10]. Furthermore, the stress induced by high drug concentration may lead to the modification in EV composition as such vesicles might carry stress-responsive proteins, altered lipids along with their peroxidation products and specific nucleic acids targeted for adaptive or signalling purposes, which reflect the unique adaptive responses to stress and contribute to effective intercellular communication [24,41, 42].

Among the effects of antifungals presented herein, the most prominent was the fungal increased capacity to produce EVs, resulting in higher EV concentrations after *C. albicans* 10231 growth in the presence of three tested drugs, compared with control conditions without added antifungals. As we demonstrated, the most significant increase was achieved for culture carried out in the presence of CASP. Nevertheless, also, fungal cells grown in the presence of AMB and FLU released a comparable number of EVs, higher than cells grown under control conditions. The same tendency in the presence of CASP was observed for the production of vesicles by three strains of *C. auris* – B8441, MMC1 and B11244 – and by *Saccharomyces cerevisiae* [43,45]. The treatment of *S. cerevisiae* with CASP applied in two concentrations (0.01 and 0.025 µg ml^−1^) showed that the number of vesicles, related to the protein amount, increased in a CASP dose-dependent manner [45]. As demonstrated by Amatuzzi *et al*. [44], the number of isolated EVs after growth of *C. auris* in the presence of CASP at a concentration of 12.5 ng ml^−1^ (strain MMC1), 10 ng ml^−1^ (strain B11244) or 100 ng ml^−1^ (strain B8441) was noticeably greater, while their morphology was found to remain a typical cup shape and unchanged despite CASP treatment. Remarkably, the average sizes of isolated particles increased for vesicles obtained from cells cultured with CASP [44], which also corroborated the findings presented by Munhoz da Rocha *et al*. [43]. In the case of EVs from *C. albicans* 10231, we observed that the mode value of vesicle diameter for EV_CASP_ was significantly increased relative to the mode value measured for EV_CON_.

Since antifungal agents exert their effects by targeting critical components of fungal cell structure and function, thereby inducing stress responses, they might strongly impact EV production efficiency [3,10]. FLU inhibits lanosterol 14*α*-demethylase Erg11, disrupting ergosterol biosynthesis and compromising membrane integrity and fluidity, which may alter vesicle trafficking and stimulate EV release. AMB binds directly to membrane ergosterol, forming pores and causing oxidative stress and membrane damage, which may also enhance EV production as a damage response. CASP targets *β*-1,3-glucan synthase, weakening the fungal cell wall and increasing cell wall rearrangement, thus also facilitating the release of EVs from the fungal cell. In addition, the subsequent activation of the cell wall integrity pathway may influence EV release as part of the fungal response to echinocandin-induced stress. This drug-related stress response, mediated also by molecular chaperone Hsp90, effectively supports cellular communication and adaptation [11]. Thereby, released EVs may improve fungal survival under antifungal pressure.

The greater number of vesicles released in the presence of CASP may result in the increase of CASP resistance, as the vesicular proteinaceous cargo may have the protective function for yeast cells, rescuing them from the cell wall disturbance caused by antifungal drug [45,46]. Zhao *et al*. investigated in detail the mechanism by which *S. cerevisiae* EVs from the WT and the *vps23*Δ strain lacking the ESCRT component protected yeast cells from CASP [45]. Their findings highlighted the critical role of the vesicular protein content and suggested that the protective mechanism involves the transfer of EV cargo proteins into cells subjected to drug-induced stress. Direct binding and trapping of CASP by the EV membrane as a protective mechanism were excluded. Prominently, the level of Chs3 protein was significantly elevated in the vesicles of the *vps23*Δ mutant strain, which conferred notable protection to yeast cells against CASP [45]. Since our study also demonstrated an increased Chs3 level in EV_CASP_, it can be speculated that the protective mechanism might be similar, involving enzymatic support for the rearrangement of the cell wall compromised by the drug, as such EVs may play a compensatory role for impaired fungal cells. Moreover, as the presence of a subinhibitory concentration of CASP increased EV production in response to the antifungal action and disruption of cell wall structure, such a targeted yeast response to the drug may contribute further to the protection of naïve fungal cells from CASP, as demonstrated previously for *S. cerevisiae* [45] and in the current study for *C. albicans*. In the case of increased resistance of fungal cells to AMB, EVs have been implicated as important contributors, serving as suppliers of membrane components that sustain the structural and functional integrity of the cell membrane during drug exposure. Additionally, EVs deliver enzymatic proteins whose activity mitigates the membrane disorders induced by the drug action [22]. In the case of FLU, the enzymatic proteins within EVs have likewise been hypothesized to play a compensatory role, facilitating cellular recovery mechanisms under antifungal stress [17]. It can be hypothesized that analogous mechanisms may play a significant role in the context presented in this work. Considering the involvement of EVs in the tolerance of biofilm-forming cells to antifungal drugs, which was demonstrated previously for *C. albicans* with CASP and FLU treatment [17,20], our current studies have further confirmed this phenomenon. Specifically, we observed that EVs produced in the presence of antifungal drugs confer protective effects against these applied agents. Notably, this protection was even greater than that conferred by EVs derived under control conditions. These findings underscore the critical role of EVs as a stress response mechanism in facilitating intercellular communication and promoting cellular adaptation.

Furthermore, modifications in culture conditions, beyond the presence or absence of antifungal drugs, significantly influence the characteristics of EVs produced by fungal cells [24,47]. Key environmental factors include changes in nutrient availability, host-like conditions and stress conditions, affecting also fungal cell morphology. Our previous studies demonstrated that under oxidative stress or increased concentration of CO_2_, the number of vesicles released by *C. albicans* ATCC 10231 was higher compared with control conditions [24].

The functional categorization of proteins identified in EVs indicated that the protein abundance in respective groups was quite comparable for all types of EVs studied in this work and encompassed proteins related to genetic information processing, transcription and translation, protein folding and sorting, transport and catabolism, stress response and virulence, signalling and cellular processes, but primarily in the metabolism of various groups of molecules, both in the cytoplasm and within the fungal cell wall. In several other previous studies, various enzymes involved in cell wall maintenance and remodelling have also been identified in *C. albicans* EVs [48]. The proteins involved in cell wall glucan metabolism and synthesis, including 1,3-beta-glucanosyltransferase Pga4, Mp65 and glucan 1,3-beta-glucosidases Bgl2 and Exg1, were identified in the cargo of EVs released by *C. albicans* ATCC 90028 and clinical isolate under the conditions of limited availability of nutrients [49], and the presence of Mp65, Bgl2 and Eng1 was also demonstrated in both yeast and hyphal EVs produced by *C. albicans* SC5314 [47]. Furthermore, in EVs released by *C. albicans* 90028 investigated in the study by Zamith-Miranda *et al*. [50], enrichment in proteins such as enolase, yeast-form wall protein Ywp1, Bgl2 or Mp65 was observed when compared with the set of proteins from *C. albicans* whole cells. In the EVs studied in this work, these proteins were also present abundantly in each type of tested vesicles, while for the first two abovementioned proteins, and additionally for Eng1, the NSAFs were slightly higher for EV_CASP_ in comparison with other EVs. Proteins Eng1 and Ywp1 play a vital role in the regulation of *β*-glucan exposure at the fungal cell surface and Dectin-1-dependent activation of macrophages by *C. albicans* cells, thus being involved in balancing immune protection with immunopathogenic mechanisms [51]. The process of controlling glucan exposure by these enzymes might also be regulated by the presence of antifungal drugs, FLU and CASP [51]. Furthermore, it was also shown previously that CASP treatment increased chitin synthase 3 (Chs3) levels in *C. albicans* cells [52,53], and in this work also in EV_CASP_, an increased relative abundance of this protein was observed compared with EVs produced by *C. albicans* under other conditions. In *S. cerevisiae* EVs, Chs3 transported as a vesicular cargo protein rescued yeast cells from the action of CASP up to the concentration of 0.1 µg ml^−1^ of antifungal drug, thus confirming the role of fungal vesicles in the modelling of the yeast cell wall and in antifungal resistance [45]. In conclusion, considering additionally the findings on the mechanism of cell wall rearrangement in *C. albicans* as a result of CASP action – where the induction of *β*-1,3-glucan exposure and an increase in chitin synthesis occur due to the intercellular regulation via the calcineurin and Mkc1 mitogen-activated protein kinase pathways [53,54] – it can be assumed that this phenomenon might influence the increased release of EVs due to the restructuring of the polysaccharide cell wall structure, and furthermore, the enzymatic proteins encapsulated within EVs might be engaged in multifaceted interactions with the fungal cell wall and cells, conferring an advantage to the pathogen in response to antifungal drug action and in modulating the host immune response. Additionally, protein Sur7, an important component of the plasma membrane of *C. albicans* cells, a key regulator of the membrane organization process and proposed proteinaceous marker for *C. albicans* EVs, was identified in EVs in this work, as well as in other studies [16,20, 39, 47, 55].

As previously shown, treatment of *C. albicans* cells with azole drugs resulted in the upregulation of *ERG11* expression [56], while in these studies, the protein encoded by this gene (Erg11, lanosterol 14*α*-demethylase; cytochrome P450 family) also had a slightly increased relative abundance in EVs produced by cells grown in the presence of FLU, which confirms this profile of cell response to the presence of the drug [57]. The presence of protein Erg7 (2,3-epoxysqualene-lanosterol cyclase, lanosterol synthase) only in EV_AMB_ and EV_FLU_ and higher NSAFs for secreted aspartic proteinase Sap9 in EV_CASP_ and EV_FLU_ might also be related to the mechanism of fungal cell response to the action of the antifungal drugs [58,59].

The analysis of zeta potentials of yeast EVs confirmed that they possess negative values similar to EVs produced by other micro-organisms [60]. In our previous work, we demonstrated that these values were in the range of −23 and −28 mV for vesicles released by *C. albicans* 10231 cells grown at YPD agar plates for 24 h at 30 °C under various growth conditions and were significantly different for EVs produced by yeast cells grown in the presence of oxidative stress compared with control conditions [24]. Then, in the work of Honorato *et al*. [61], a zeta potential value of –24.53 mV was also reported for EVs produced by *C. albicans* 90028 cells cultured in Sabouraud broth for 48 h at 30 °C. In this study, the measured zeta potential values of vesicles were in a similar range of −25.4 to −26.5 mV, with no statistically significant differences observed between EVs released by cells grown in the presence or absence of the antifungal drugs. Therefore, it could be assumed that the surface charges of tested EVs were quite comparable; however, other properties of fungal EVs might also influence the interactions between vesicles and host cells or liposomes modelling the host cell membrane. Initially, such interactions were also studied by measuring changes in zeta potential upon contact, but in regard to human cells or prepared liposomes. The most significant changes in the zeta potential of cells or liposomes were observed after the addition of EV_CASP_ in both analysed models. Notably, these changes were well demonstrated in the interactions of EVs with whole U-937 cells, indicating the role of proteins present in the lipid bilayer of the cell membrane. A similar effect was observed when measuring the interaction of U-937 cells with fungal vesicles released by *C. albicans* under oxidative stress conditions [24].

The differences in the modulation of host immune responses may vary due to the factors influencing the release of EVs by candidal cells. In the studies by Martínez-López *et al*., for yeast- and hyphal-derived EVs of *C. albicans* SC5314, the stimulation of THP-1 macrophages resulted in high levels of TNF-*α* cytokine when using vesicles released by hyphal cells, while no TNF-*α* production was observed after stimulation with yeast-derived EVs, and the levels of other cytokines – IL-10 and IL-12 – were reported as not changed [47]. As described in our previous study for THP-1 cells, the levels of secreted IL-8 and TNF-*α* were greater for EVs produced under stress conditions, with the most significant changes observed for EVs released under oxidative stress. The most pronounced changes in IL-10 production were previously detected for EVs released under conditions of host CO_2_ concentration, whereas no significant changes were noted for IL-1*β* [24]. In the current study, a higher level of IL-10 released by THP-1 macrophage-like cells in comparison with EV_CON_ was reported for vesicles derived from cells cultured in the presence of FLU and CASP, while for EV_CON_ and EV_AMB_, these levels were reduced compared with control cells not stimulated with vesicles. Furthermore, the level of IL-8 produced by THP-1 cells was increased after stimulation with all tested types of EVs, which may suggest their pro-inflammatory properties. However, considering the variability in IL-10 production, the potential differences in immunomodulation among the vesicles must be considered. The diversity in host responses reported thus far in several studies is associated with the varying types of host cells and the origins of fungal EVs [19,24, 47, 50, 61,63]. However, these reports refer to EV-producing fungal cells that did not grow in the presence of antifungal drugs. In the present study, we have demonstrated that EVs produced during the treatment of fungal infection and the administration of antifungal therapeutics can elicit a similar pro-inflammatory effect in human cells as vesicles produced by *C. albicans* cells untreated with antifungal drugs. However, subtle differences may be observed, particularly for EV_CASP_, potentially attributable to polysaccharide rearrangement during drug treatment, the presence of different cell wall-related enzymes as vesicle cargo and the altered properties of EVs under these conditions.

For CASP-treated fungal cells, the increased exposure of cell wall mannoproteins was reported for *C. auris* [40], and for *C. albicans*, a higher mannan content in the cell wall was demonstrated [54], accompanied by the induction of *β*-1,3-glucan exposure and an increase in chitin synthesis [52,53]. As all major polysaccharide components of the yeast cell wall were also identified in *C. albicans* EVs, including mannans, chitin and *β*-1,3-glucan [61], together with numerous enzymes integral to their rearrangement, drug effects that have consequences in their altered content and presentation may have a substantial impact on the interactions with monocytic cells or macrophages, as highlighted in the work by Yadav *et al*. where it has been demonstrated that monocytes recognize fungal cells mainly through mannan and macrophages through glucan [63], and depending on the extent of exposure to these molecules, particular immune cells initiate defence mechanisms; conversely, chitin modulates the immune response by attenuating inflammation through the induction of IL-10 production [64]. Thus, the differences demonstrated herein related to the vesicular enzymatic cargo after antifungal treatment might correspond to differences in the abundance of these molecules in vesicles, and such variations could potentially account for the differential effects of drug-modulated EVs on monocytic and macrophage-like cells, as well as on the model organism in *in vivo* studies presented in our studies.

In the *in vivo* studies carried out in this work, the decrease in the survival of *G. mellonella* larvae after injection of distinct types of EVs was rather negligible and comparable to individuals injected with suspension buffer. Only a very minor difference was observed for EVs produced in the presence of antifungal agents; however, this did not reach statistical significance. This may indicate that these investigated vesicular structures were not directly cytotoxic to the host, a conclusion also corroborated by prior cellular assays. Importantly, the ability of EVs to elicit an immune response in the host suggests their potential as candidates for immunization and prevention strategies against *C. albicans* infections [61,62]. In the case of *G. mellonella* larvae, we assessed this activation of the host immune response by measuring phenol oxidase activity in the haemolymph of larvae following EV injection. This enzyme plays a crucial role in the immune defence by contributing to melanin synthesis and antimicrobial activity, as phenol oxidase catalyses the conversion of phenols to quinones, which subsequently polymerize to form melanin around pathogens, and this melanization process supports to encapsulate and eradicate invading micro-organisms [34,35, 65]. A significant increase in the enzymatic activity of phenol oxidase was observed only for EV_CON_ when compared with larvae injected with buffer, and not for vesicles produced by *C. albicans* cells treated with antifungal drugs. This may suggest their differential capability, especially noticeable for EV_CASP_, to modulate the mechanisms of host defence response during exposure to pathogenic factors.

Therefore, the observations presented in this work regarding the *in vitro* and *in vivo* functionality tests of EVs highlight the complex interplay between fungal EVs, antifungal treatment and the host immune response. This indicated the need for a more detailed investigation of these multifaceted interactions due to the potential of EVs produced in the presence of subinhibitory concentrations of antifungal drugs to influence the course of infection caused by *C. albicans*.

## Supplementary material

10.1099/mic.0.001565 Fig. S1.

## Abbreviations

AMB: amphotericin B
CASP: caspofungin
DPBS: Dulbecco’s PBS
EV: extracellular vesicle
FLU: fluconazole
LDH: lactate dehydrogenase
NSAFs: normalized spectral abundance factors
NTA: nanoparticle tracking analysis
PMA: phorbol 12-myristate 13-acetate
TEM: transmission electron microscopy
XTT: sodium 3′-[1-(phenylaminocarbonyl)-3,4-tetrazolium]-bis (4-methoxy6-nitro) benzene sulfonic acid hydrate
YPD: yeast extract peptone dextrose
