# Supplementary material for: Functional properties of Candida albicans extracellular vesicles released in the presence of the antifungal drugs amphotericin B, fluconazole and caspofungin
Source: Microbiology (Reading). 2025 Jun 12;171(6):001565. doi: 10.1099/mic.0.001565 (PMC12282285; doi:10.1099/mic.0.001565)
Supplement: Fig. S1. [file mic-171-01565-s001.pdf]

Functional properties of *Candida albicans* extracellular vesicles released in the presence of the antifungal drugs amphotericin B, fluconazole and caspofungin

Kamila Kulig[1], Elzbieta Rudolphi-Szydło[2], Anna Barbasz[2], Magdalena Surowiec[1], Ewelina Wronowska[1], Katarzyna Kowalik[1,3], Dorota Satala[1], Grazyna Bras[1], Olga Barczyk-Woznicka[4], Elzbieta Karnas[5], Elzbieta Pyza[4], Ewa Zuba-Surma[5], Maria Rapala-Kozik[1], Justyna Karkowska-Kuleta[1,*]

[1] Department of Comparative Biochemistry and Bioanalytics, Faculty of Biochemistry, Biophysics and Biotechnology, Jagiellonian University, Gronostajowa 7, 30-387 Kraków, Poland
[2] Department of Biochemistry and Biophysics, Institute of Biology and Earth Sciences, University of the National Education Commission, Podchorazych 2, 30-084 Kraków, Poland
[3] Doctoral School of Exact and Natural Sciences, Faculty of Biochemistry, Biophysics and Biotechnology, Jagiellonian University, Gronostajowa 7, 30-387 Kraków, Poland
[4] Department of Cell Biology and Imaging, Institute of Zoology and Biomedical Research, Jagiellonian University, Gronostajowa 9, 30-387 Kraków, Poland
[5] Department of Cell Biology, Faculty of Biochemistry, Biophysics and Biotechnology, Jagiellonian University, Gronostajowa 7, 30-387 Kraków, Poland
[*] correspondence: justyna.karkowska@uj.edu.pl

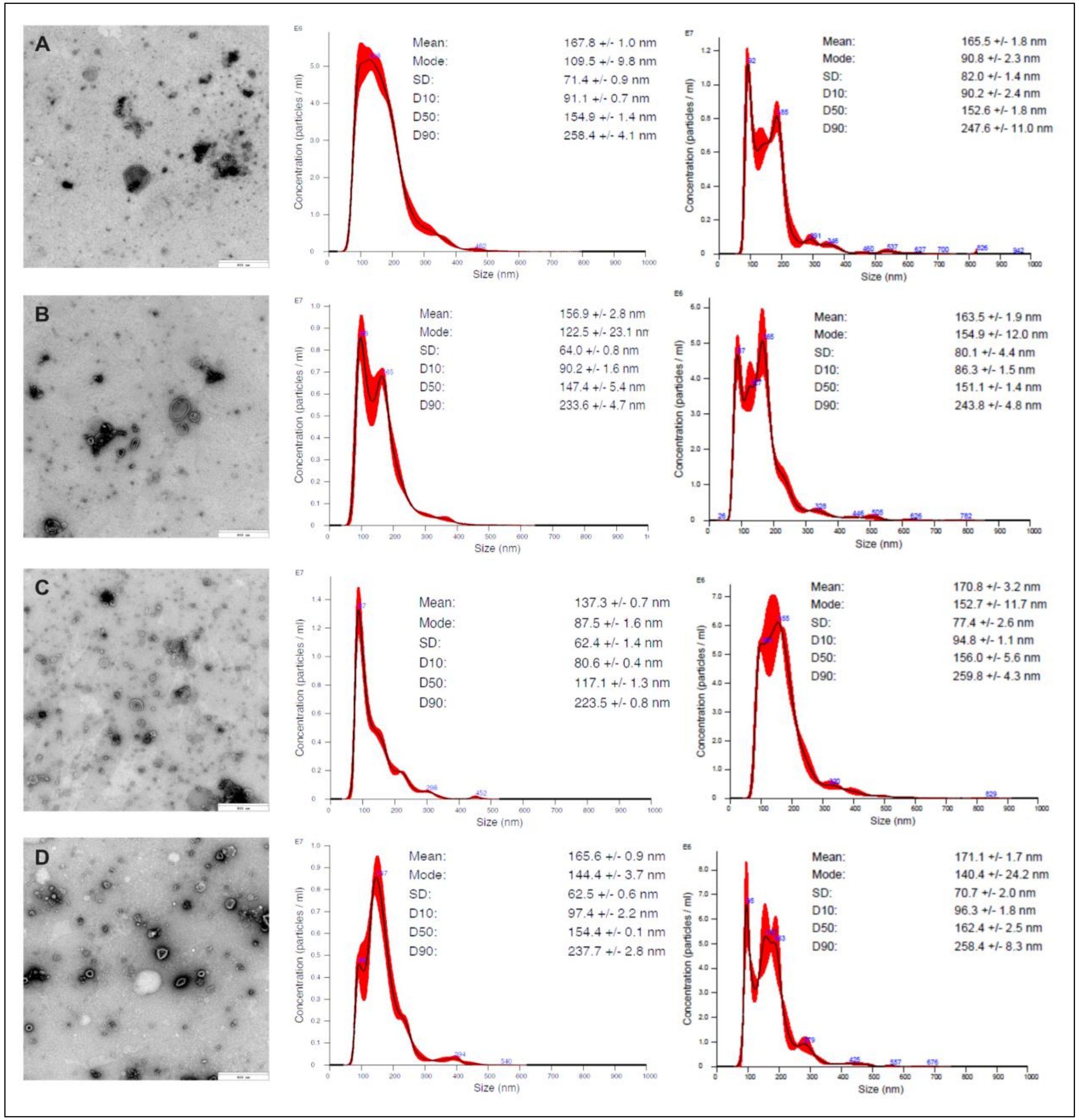

Fig. S1. Characteristics of EVs produced by *C. albicans*. TEM photographs and NTA particle size distribution analysis of EV_{CON} (A), EV_{AMB} (B), EV_{FLU} (C), and EV_{CASP} (D). Scale bar: 800 nm. Representative histograms of the average size distribution from three measurements of a single sample (black line) are presented. The indicated blue numbers indicate the maxima of peaks, and the standard deviations (SD) between measurements are marked with the red areas. The size parameters of the EVs are presented for each histogram. The values D10, D50, and D90 represent the diameters at which 10%, 50%, and 90% of the EV population are equal to or smaller than the given measurements.

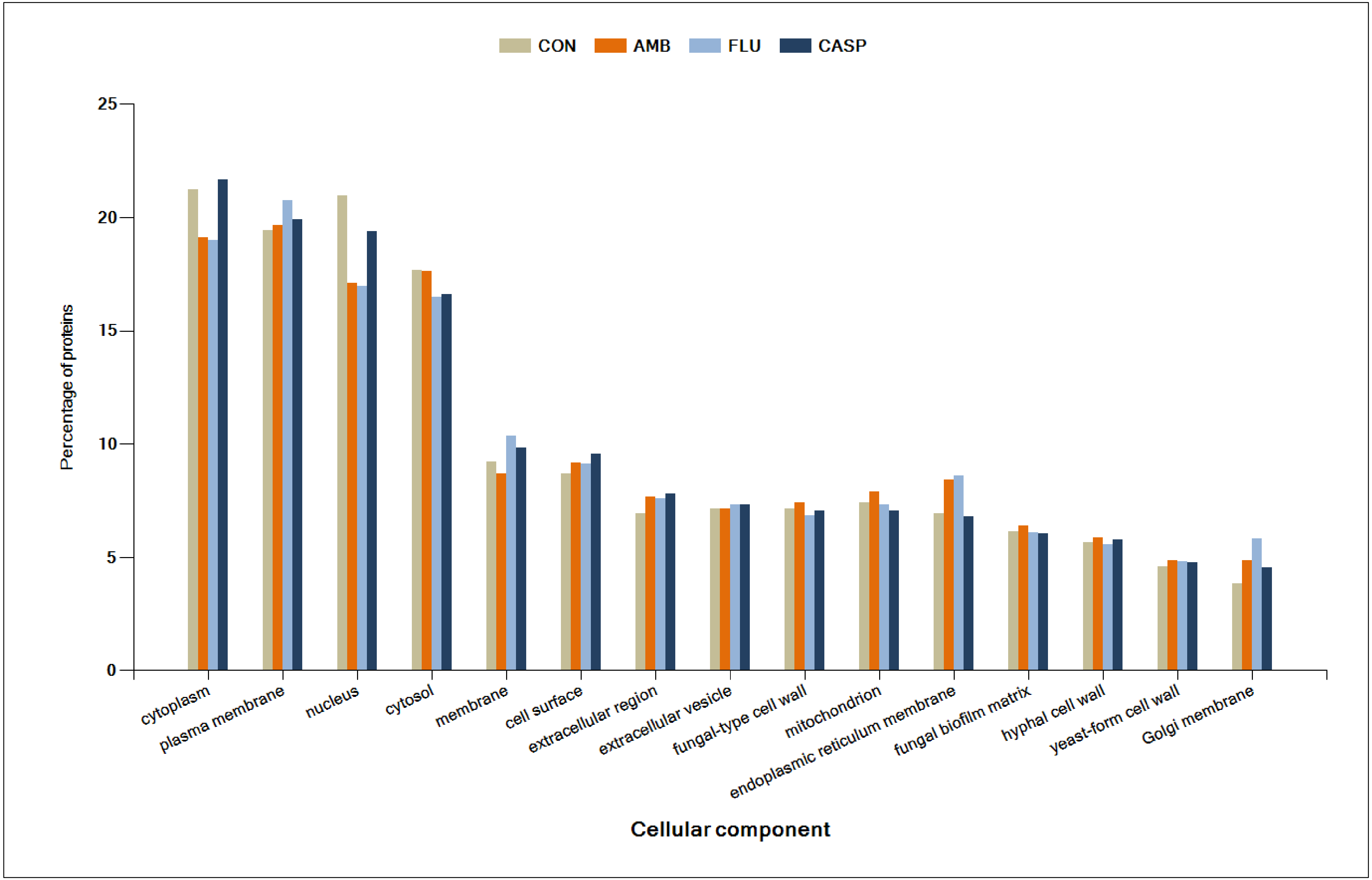

Fig. S2. The classification by cellular location of proteins identified in EVs produced by *C. albicans* cells cultured in the presence of antifungal drugs (AMB – amphotericin B; CASP – caspofungin, FLU – fluconazole; CON – control conditions without antifungal drugs added). Created using the enrichment analysis tool FunRich v. 3.1.3.

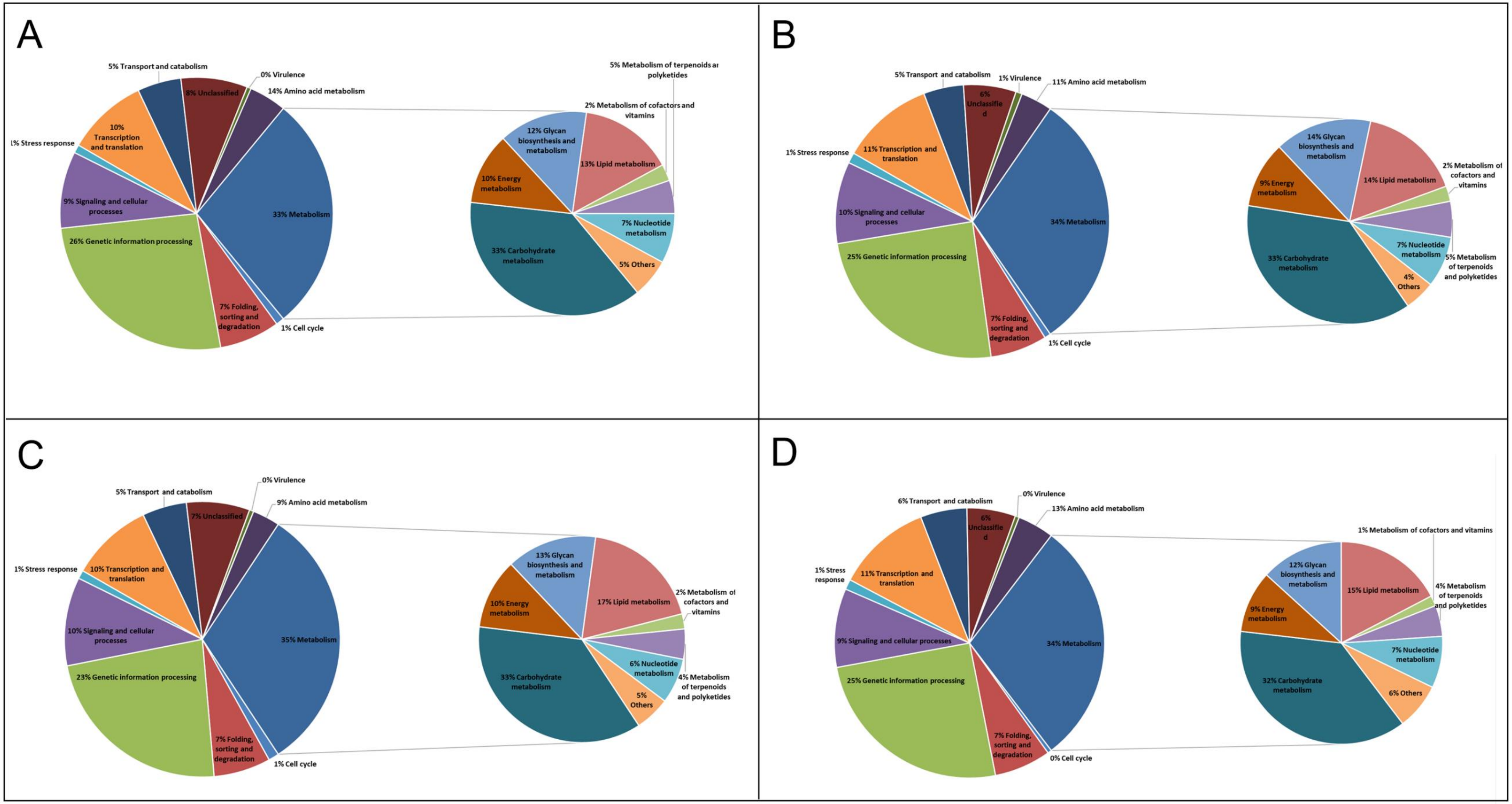

Fig. S3. The classification by function assigned to proteins identified in EVs produced by *C. albicans* cells cultured under control conditions without antifungal drugs added (A) and in the presence of antifungal drugs (B) amphotericin B, (C) fluconazole, (D) caspofungin. Functions assigned on the basis of the Uniprot protein database.

Table S1. Mass spectrometry identification of *Candida albicans* proteins in EVs. The proteomic analysis of extracellular vesicles was performed with samples prepared with a total amount of proteins equal to 11 μg. After digestion with Trypsin/Lys-C Mix the resulting peptides were analyzed using UltiMate 3000 RSLCnano System coupled with Q-Exactive mass spectrometer with DPV-550 Digital PicoView nanospray source. The obtained lists of peaks were searched against the Swiss-Prot protein database with the locally installed MASCOT search engine using the taxonomy restrictions: Fungi. The Target Decoy PSM Validator was applied with the maximum false discovery rate (FDR) for peptides set to 0.01. Proteins identified with a score > 14 and a number of identified peptides ≥ 1 are only listed. PSM – peptide sequence matches; AAs – amino acids

Accession number	Description	Molecular mass [kDa]	Score	Coverage	# Proteins	# Unique Peptides	# Peptides	# PSMs	# AAs	calc. pI	NSAF
**EV_{CON}**											
Q92211	Glyceraldehyde-3-phosphate dehydrogenase	35.8	8338.49	84.18	44	25	25	281	335	7.12	0.089772
P83779	Pyruvate decarboxylase	62.4	5594.22	60.67	4	24	24	137	567	5.58	0.025859
P25997	Elongation factor 3	116.9	4560.49	46.76	7	41	41	119	1050	5.73	0.012129
P41797	Heat shock protein SSA1	70.3	3735.56	54.57	9	10	28	115	656	5.17	0.018762
P46587	Heat shock protein SSA2	70.0	3532.72	64.65	2	15	32	106	645	5.06	0.017588
P43067	Alcohol dehydrogenase 1	36.9	3257.79	57.14	3	14	16	118	350	6.44	0.036082
P34731	Fatty acid synthase subunit beta	227.8	3054.30	38.68	5	59	59	80	2037	6.02	0.004203
P0CY35	Elongation factor 1-alpha 1	50.0	2820.16	43.89	16	4	19	121	458	9.03	0.028275
P87222	Heat shock protein SSB1	66.4	2779.30	54.81	9	23	23	82	613	5.38	0.014316
P43098	Fatty acid synthase subunit alpha	207.5	2303.95	34.48	7	46	46	56	1885	5.62	0.003179
P28877	Plasma membrane ATPase 1	97.4	2216.02	29.16	8	14	22	58	895	4.96	0.006936
Q9URB4	Fructose-bisphosphate aldolase	39.2	2007.44	72.14	3	19	19	56	359	6.06	0.016695
C4YJQ8	Elongation factor 2	93.3	1870.82	39.19	13	28	28	50	842	6.47	0.006355
P14235	Actin	41.7	1854.84	48.67	30	14	14	43	376	5.69	0.012239
Q96VB9	Heat shock protein homolog SSE1	78.5	1694.88	55.92	6	32	32	43	701	5.30	0.006565
P83776	Hexokinase-2	53.4	1586.40	55.99	3	21	23	47	484	5.55	0.010393
P43076	pH-responsive protein 1	59.4	1266.02	43.25	2	20	21	25	548	5.07	0.004882
Q9Y872	Sulfate adenylyltransferase	58.8	1259.90	55.79	20	23	23	34	527	6.40	0.006905
P83784	Heat shock protein SSC1, mitochondrial	69.7	1227.18	39.81	1	20	20	29	648	5.60	0.004790
O13434	Phosphoenolpyruvate carboxykinase [ATP]	60.8	1141.26	51.90	2	19	19	26	553	6.60	0.005032
O94039	Transketolase 1	73.7	1060.42	38.70	1	20	20	27	677	5.82	0.004268
P30575	Enolase 1	47.2	1053.67	39.77	13	13	13	23	440	5.81	0.005594
P83778	Malate dehydrogenase, cytoplasmic	36.0	1023.43	61.42	1	15	15	22	337	5.62	0.006987
Q5AML1	Eukaryotic translation initiation factor 3 subunit C	99.8	944.91	32.27	7	22	22	26	874	5.15	0.003184
P40910	40S ribosomal protein S1	29.0	936.32	57.81	53	16	16	27	256	10.04	0.011288

P46598	Heat shock protein 90 homolog	80.8	926.10	37.77	7	21	21	24	707	4.88	0.003633
Q5A8K2	Alanine--tRNA ligase	108.2	903.58	28.17	3	23	23	28	969	6.07	0.003093
O42817	40S ribosomal protein S0	28.7	889.08	61.30	33	12	12	30	261	4.91	0.012302
P47837	40S ribosomal protein S4	29.2	857.62	46.18	1	10	13	31	262	10.21	0.012663
O94049	Acetyl-coenzyme A synthetase 1	75.1	850.69	32.89	6	19	21	28	675	6.32	0.004440
C4YKT4	Ras-like protein 1	32.2	827.34	35.76	2	6	6	11	288	4.67	0.004088
Q8NJN3	Acetyl-coenzyme A synthetase 2	73.8	824.03	32.04	13	16	18	25	671	6.13	0.003987
P46273	Phosphoglycerate kinase	45.2	815.94	54.44	14	19	19	25	417	6.48	0.006416
Q59KZ1	Aminopeptidase 2	104.3	814.98	28.90	2	22	22	22	924	5.36	0.002548
Q59PT0	V-type proton ATPase subunit B	57.2	805.29	45.51	5	2	18	21	512	5.03	0.004390
P43071	Multidrug resistance protein CDR1	169.8	798.22	15.79	6	16	18	21	1501	6.98	0.001497
Q5AKA5	Cys-Gly metallodipeptidase DUG1	53.6	796.42	40.41	1	13	13	17	485	5.24	0.003751
Q9HGT6	Serine--tRNA ligase, cytoplasmic	53.0	789.56	43.72	4	18	18	21	462	5.73	0.004865
O74676	ABC transporter CDR4	169.2	766.48	17.85	7	18	20	20	1490	7.65	0.001437
Q59KI0	UTP--glucose-1-phosphate uridylyltransferase	55.5	760.52	29.60	3	11	11	15	500	6.73	0.003211
P83774	Guanine nucleotide-binding protein subunit beta-like protein	34.5	747.66	52.37	1	11	11	19	317	6.54	0.006415
O74261	Heat shock protein 60, mitochondrial	60.1	740.47	32.16	6	13	13	17	566	5.30	0.003215
P83777	Inorganic pyrophosphatase	32.1	722.85	60.07	12	12	12	17	288	5.26	0.006317
Q5A4E2	ATP-dependent RNA helicase DED1	72.8	715.18	33.63	47	14	16	21	672	8.28	0.003345
P83782	Cytochrome b-c1 complex subunit 2, mitochondrial	39.5	701.52	60.43	1	16	16	17	374	5.57	0.004865
P52495	Ubiquitin-activating enzyme E1 1	114.2	673.63	20.76	2	15	15	16	1021	5.01	0.001677
O93827	Mannose-1-phosphate guanyltransferase	40.0	665.50	57.46	15	19	19	22	362	6.30	0.006504
O94083	Eukaryotic translation initiation factor 5A	17.1	662.73	52.53	6	7	7	15	158	5.05	0.010161
P82611	Aconitate hydratase, mitochondrial	84.2	657.32	27.93	8	17	17	21	777	6.39	0.002893
P82610	5-methyltetrahydropteroyltriglutamate--homocysteine methyltransferase	85.6	652.32	23.73	3	12	13	16	767	5.60	0.002233
P53704	Glutamine--fructose-6-phosphate aminotransferase [isomerizing]	79.2	647.75	25.53	3	13	13	16	713	6.24	0.002402
Q5AHH4	Small heat shock protein 21	21.5	645.57	63.49	1	10	10	20	189	5.35	0.011325
Q9P843	60S ribosomal protein L27	15.5	626.03	52.21	2	8	8	18	136	10.18	0.014165
P83781	Mitochondrial outer membrane protein porin	29.7	610.39	53.90	1	12	12	15	282	8.57	0.005693
Q5AJD0	ATP-dependent RNA helicase DBP5	60.2	605.72	29.63	25	12	12	14	540	5.76	0.002775
P53707	37 kDa cell surface protein	37.0	603.09	35.83	4	12	12	16	321	7.06	0.005335
O13289	Peroxisomal catalase	54.8	598.87	28.25	2	11	11	13	485	6.65	0.002869

O13354	Eukaryotic peptide chain release factor GTP-binding subunit	79.0	589.78	22.94	7	12	12	14	715	8.47	0.002096
Q00310	Glycolipid 2-alpha-mannosyltransferase 1	50.0	586.97	40.37	2	12	14	16	431	6.90	0.003973
Q96UX5	Heat shock protein 78, mitochondrial	91.6	580.34	21.92	2	11	11	13	812	6.80	0.001713
Q5ALX3	Transcription elongation factor SPT5	105.8	574.74	16.63	2	12	12	12	956	5.16	0.001343
P47828	T-complex protein 1 subunit theta	58.9	568.21	29.81	2	13	13	13	540	7.97	0.002577
Q5AJB1	V-type proton ATPase catalytic subunit A	67.6	554.91	17.18	6	8	8	10	617	5.27	0.001735
Q5AAU3	Protein transport protein SEC31	136.2	549.02	14.78	4	13	13	14	1265	6.77	0.001184
P10613	Lanosterol 14-alpha demethylase	60.6	545.16	30.49	3	15	15	17	528	7.17	0.003446
Q5AME2	Pentafunctional AROM polypeptide	169.3	543.32	14.89	8	18	18	18	1551	6.58	0.001242
Q5AGV4	Eukaryotic translation initiation factor 3 subunit B	84.2	542.48	23.00	5	13	13	17	739	6.39	0.002462
O13287	6-phosphogluconate dehydrogenase, decarboxylating	56.9	542.40	34.82	4	13	13	16	517	6.57	0.003312
P10875	Tubulin beta chain	49.9	542.35	30.29	13	10	10	15	449	4.74	0.003575
P43084	Probable NADPH dehydrogenase	46.0	540.47	41.52	2	14	14	17	407	6.39	0.004470
Q9UVJ4	60S ribosomal protein L10a	24.4	533.53	36.87	4	9	9	14	217	9.76	0.006905
P87206	ATP-dependent RNA helicase eIF4A	44.6	532.73	51.13	40	16	16	16	397	5.36	0.004313
Q59ZX6	U3 small nucleolar RNA-associated protein 10	204.2	528.28	7.92	5	11	11	12	1818	5.87	0.000706
O94038	Alcohol dehydrogenase 2	36.8	518.24	31.32	7	7	9	13	348	6.68	0.003998
Q5AIR7	Endo-1,3(4)-beta-glucanase 1	124.0	514.60	13.36	2	12	12	13	1145	5.40	0.001215
O94201	ATP-dependent 6-phosphofructokinase subunit alpha	108.5	511.53	23.00	3	18	19	19	987	6.62	0.002060
O42825	GTP-binding protein RHO1	22.0	498.51	52.53	7	9	9	13	198	5.73	0.007027
P30573	Chitin synthase 3	136.1	490.77	13.93	9	13	13	16	1213	7.17	0.001412
Q59LU0	ATP-dependent RNA helicase DBP2	61.2	490.65	39.68	33	19	20	24	562	8.88	0.004570
Q5AJY5	1,3-beta-glucanosyltransferase PGA4	49.0	480.82	22.84	1	8	8	9	451	4.74	0.002136
O13432	Phenylalanine--tRNA ligase beta subunit	66.8	479.59	27.03	2	15	15	16	592	5.35	0.002893
Q5APD4	Sphingolipid C9-methyltransferase	58.7	473.20	23.98	2	11	11	13	513	6.92	0.002712
Q5A0W7	RuvB-like helicase 1	50.0	466.31	36.46	9	10	10	10	458	6.01	0.002337
O42766	14-3-3 protein homolog	29.5	465.73	30.30	8	7	7	11	264	4.81	0.004459
P43057	Protein kinase C-like 1	125.2	464.26	10.03	1	9	9	10	1097	7.42	0.000976
O13318	pH-responsive protein 2	58.7	454.40	15.63	1	7	8	9	544	4.64	0.001771
A0A1D8PTW6	Hydroxymethylglutaryl-CoA synthase	49.7	448.68	23.06	6	8	8	9	451	5.97	0.002136
Q59S78	Small COPII coat GTPase SAR1	21.5	440.62	57.37	17	8	8	12	190	5.59	0.006759
Q5AEN1	Cytochrome c peroxidase, mitochondrial	40.7	437.49	39.62	1	11	11	14	366	6.34	0.004094

O94008	60S ribosomal protein L32	14.9	436.72	41.98	1	7	7	17	131	10.54	0.013889
Q9P940	Triosephosphate isomerase	26.6	434.63	52.82	3	10	10	12	248	6.01	0.005179
Q5A4M8	Protein SUR7	29.9	428.47	32.22	1	5	5	7	270	7.69	0.002775
Q5AK59	ATP-dependent RNA helicase HAS1	63.0	427.61	19.65	30	8	8	9	565	8.91	0.001705
Q5AI15	Polyadenylate-binding protein, cytoplasmic and nuclear	70.4	419.03	21.46	9	11	11	14	629	5.29	0.002382
O13426	Serine hydroxymethyltransferase, cytosolic	52.0	417.44	31.70	7	15	15	18	470	7.20	0.004099
O74198	Sterol 24-C-methyltransferase	43.0	416.36	39.89	3	11	11	14	376	6.11	0.003985
Q59M70	NADH-cytochrome b5 reductase 2	33.4	413.32	34.22	2	10	10	13	301	8.48	0.004622
O94200	ATP-dependent 6-phosphofructokinase subunit beta	104.0	410.39	14.38	3	11	12	13	946	6.35	0.001471
Q96W53	40S ribosomal protein S14	14.4	409.96	43.28	4	7	7	10	134	10.61	0.007987
Q59PR9	Transcriptional regulator HMO1	24.8	409.70	38.12	1	6	6	10	223	9.19	0.004799
Q5AAW3	ATP-dependent RNA helicase DHH1	62.1	398.68	19.67	8	9	9	12	549	8.75	0.002339
P29717	Glucan 1,3-beta-glucosidase	50.0	398.37	32.88	3	8	8	10	438	5.64	0.002443
Q8TGH6	Guanosine-diphosphatase	65.9	398.22	19.20	1	9	9	10	599	5.94	0.001787
Q96W54	40S ribosomal protein S22	14.8	388.53	64.62	9	8	8	10	130	9.88	0.008233
O43101	Centromere/microtubule-binding protein CBF5	54.3	380.36	32.36	2	10	10	11	479	9.20	0.002458
Q59MQ0	Myosin-5	146.9	367.74	8.97	20	10	10	10	1316	9.35	0.000813
Q59PL9	Eukaryotic translation initiation factor 3 subunit A	106.0	356.08	15.48	5	13	13	14	930	8.31	0.001611
C4YG73	Vacuolar protein sorting/targeting protein 10	179.2	350.39	10.47	2	13	13	13	1586	5.03	0.000877
Q5AI37	Probable metalloprotease ARX1	62.2	345.86	20.74	1	10	10	10	564	6.92	0.001898
Q5A860	Translationally-controlled tumor protein homolog	18.5	337.40	47.31	2	5	5	6	167	4.46	0.003845
O59931	60S ribosomal protein L13	23.0	335.55	41.09	1	6	6	12	202	10.61	0.006358
Q92206	Squalene monooxygenase	55.3	330.51	18.55	1	7	7	9	496	8.73	0.001942
P46596	Opaque-phase-specific protein OP4	41.3	328.92	9.70	1	2	2	3	402	5.26	0.000799
Q59X38	Pescadillo homolog	67.8	327.58	18.74	5	9	9	9	587	6.19	0.001641
P87066	Tubulin alpha chain	49.9	325.78	16.07	15	6	6	9	448	5.06	0.002150
P31353	Phosphomannomutase	29.0	323.52	39.68	3	9	9	10	252	5.69	0.004247
P0CH96	Adenylosuccinate synthetase	47.9	322.60	23.83	10	9	9	10	428	7.17	0.002501
Q5AF03	Glyoxalase 3	25.8	318.59	38.98	1	5	5	8	236	4.83	0.003628
P39826	Cell division control protein 3	47.8	315.78	17.55	3	6	6	7	416	6.64	0.001801
Q5AGZ9	RuvB-like helicase 2	54.5	315.09	20.08	12	7	7	8	498	5.15	0.001719
Q9P4E9	GTP-binding nuclear protein GSP1/Ran	24.3	309.68	55.61	8	10	10	14	214	7.02	0.007002

Q5ACZ2	Mannan endo-1,6-alpha-mannosidase DFG5	50.0	308.95	16.85	1	6	6	6	451	4.65	0.001424
P79023	Phospho-2-dehydro-3-deoxyheptonate aldolase, tyrosine-inhibited	40.3	307.97	26.76	3	7	8	9	370	6.65	0.002603
O13425	Serine hydroxymethyltransferase, mitochondrial	54.5	306.33	21.30	2	8	8	8	493	8.97	0.001737
P34732	Vesicular-fusion protein SEC18	88.9	304.28	14.86	1	8	9	9	794	7.43	0.001213
Q59S06	Nucleolar protein 58	57.1	303.14	19.19	9	7	7	10	516	8.31	0.002074
Q5ALX8	Adenine phosphoribosyltransferase	20.9	302.03	38.30	1	5	5	6	188	5.31	0.003416
P47834	60S ribosomal protein L36	11.1	301.55	30.30	2	4	4	6	99	11.40	0.006486
Q9P8Q7	Isocitrate lyase	61.4	297.61	20.55	17	9	9	10	550	7.11	0.001946
Q9UW25	Oxysterol-binding protein-like protein OBPa	49.5	296.72	26.33	4	10	10	11	433	6.13	0.002719
Q5APT8	ATP-dependent RNA helicase DBP3	63.1	296.26	19.68	12	9	9	9	564	9.45	0.001708
Q59Y31	Yeast-form wall Protein 1	54.2	292.11	4.50	1	2	2	14	533	4.81	0.002811
O94017	40S ribosomal protein S16	15.7	291.71	45.07	7	6	6	10	142	10.29	0.007537
Q59ZH9	ATP-dependent RNA helicase MAK5	88.0	287.30	11.89	8	8	8	9	782	6.04	0.001232
P82612	Phosphoglycerate mutase	27.4	279.82	25.00	1	4	4	6	248	6.16	0.002589
P87220	V-type proton ATPase subunit D	30.0	270.04	24.34	3	4	4	5	267	5.85	0.002004
P83783	Adenosylhomocysteinase	49.0	267.82	24.00	4	8	8	8	450	5.59	0.001903
Q5AI86	Eukaryotic translation initiation factor 3 subunit I	38.2	262.89	22.00	9	7	7	7	350	5.47	0.002140
Q5ACU6	ATP-dependent rRNA helicase RRP3	59.8	257.98	18.54	8	7	7	8	534	9.73	0.001603
A0A1D8PH78	Farnesyl pyrophosphate synthase	40.7	255.51	17.66	1	5	5	6	351	4.98	0.001829
P43060	Phosphoribosylaminoimidazole-succinocarboxamide synthase	32.9	247.18	25.77	7	6	6	7	291	5.50	0.002574
Q59VP7	Ribosome biogenesis protein ERB1	97.3	244.34	14.02	13	7	9	9	849	4.74	0.001135
Q5ABV6	SWI5-dependent HO expression protein 3	59.2	244.11	10.02	4	4	4	5	519	9.20	0.001031
Q59MN0	Vacuolar protein 8	63.4	239.29	11.79	5	5	5	6	585	5.07	0.001098
Q5APF2	GMP synthase [glutamine-hydrolyzing]	58.8	237.15	18.68	8	8	8	8	530	5.92	0.001615
Q5ANB2	ATP-dependent RNA helicase DBP10	103.5	233.34	9.03	2	6	6	6	908	9.07	0.000707
Q5A5S7	Autophagy-related protein 27	28.3	231.91	23.02	1	4	4	4	252	5.53	0.001699
Q9Y7F0	Peroxiredoxin TSA1	21.8	230.15	32.14	4	5	5	5	196	5.06	0.002730
P43070	Glucan 1,3-beta-glucosidase	33.5	229.86	10.71	2	3	3	7	308	4.78	0.002432
Q9P975	Eukaryotic translation initiation factor 4E	24.2	227.07	27.27	1	5	5	6	209	5.26	0.003072
Q59L13	Eukaryotic translation initiation factor 6	26.4	226.64	22.45	7	4	4	5	245	4.58	0.002184
Q59XX2	Cell surface mannoprotein MP65	39.2	225.06	11.90	1	3	3	4	378	5.45	0.001133
Q5A1D5	FACT complex subunit SPT16	121.3	222.50	8.02	1	7	7	7	1060	5.16	0.000707

Q5ANE3	Non-classical export protein 102	18.1	220.79	20.59	1	2	2	4	170	9.01	0.002518
Q5A4X0	E3 ubiquitin-protein ligase BRE1	78.5	220.64	8.66	1	5	5	5	681	6.14	0.000786
G1UB61	Septin CDC11	46.7	217.00	11.94	1	3	3	4	402	5.07	0.001065
Q59R28	Alpha-1,2-mannosyltransferase MNN26	87.2	216.33	8.86	1	6	6	6	756	6.52	0.000849
P83780	Glucose-6-phosphate isomerase	61.1	216.16	17.09	5	8	8	9	550	6.40	0.001751
P47831	60S ribosomal protein L28 (Fragment)	6.6	215.81	46.77	1	3	3	7	62	9.09	0.012083
Q59Q46	Inosine-5'-monophosphate dehydrogenase	56.2	215.26	5.76	1	2	2	3	521	6.55	0.000616
Q9P8P7	Ribosomal RNA small subunit methyltransferase NEP1	29.5	210.39	30.34	2	5	5	5	267	8.43	0.002004
O93852	D-arabinono-1,4-lactone oxidase	63.4	210.33	17.77	1	7	7	7	557	6.61	0.001345
A0A1D8PN12	Glycerophosphodiester transporter GIT2	59.3	209.55	12.92	1	5	5	5	534	8.10	0.001002
C4YLH0	MICOS complex subunit MIC60	62.6	204.44	7.61	3	4	4	4	565	6.44	0.000758
Q59TU0	Nascent polypeptide-associated complex subunit beta	17.0	201.48	42.68	4	4	4	4	157	5.71	0.002727
Q59MA9	Clustered mitochondria protein homolog	155.7	196.22	7.63	14	8	9	9	1363	5.39	0.000707
Q59L72	GPI-anchored protein 52	41.4	195.78	14.58	1	4	4	5	384	4.91	0.001394
Q59KF3	AdoMet-dependent rRNA methyltransferase SPB1	96.8	190.54	6.15	3	5	5	5	845	5.76	0.000633
O74189	Dolichyl-phosphate-mannose--protein mannosyltransferase 1	99.9	184.30	6.50	1	4	4	5	877	6.95	0.000610
P87219	Sorbose reductase SOU1	30.0	183.48	17.08	1	3	3	4	281	5.30	0.001523
P0CY31	Ras-related protein SEC4	23.1	182.90	24.76	1	4	4	5	210	5.47	0.002548
A0A1D8PI71	Squalene synthase ERG9	51.2	181.73	14.96	2	7	7	8	448	6.84	0.001911
P43063	Cyclin-dependent kinase 1	36.6	181.05	21.45	15	7	7	8	317	6.61	0.002701
Q59YF0	Protein transport protein SSO2	34.3	180.58	14.58	1	4	4	5	295	5.36	0.001814
Q5A744	Protein SDS23	67.5	176.20	7.17	1	3	3	3	628	8.44	0.000511
Q5ADT9	37S ribosomal protein S10, mitochondrial	27.4	175.84	21.79	1	4	4	4	234	9.48	0.001829
O74933	UDP-N-acetylglucosamine pyrophosphorylase	54.6	171.22	13.17	1	5	5	5	486	6.28	0.001101
Q59WB3	S-adenosylmethionine permease GAP4	66.4	170.99	7.08	1	4	4	5	607	7.46	0.000882
Q59W33	Glycerol-3-phosphate dehydrogenase [NAD(+)] 2	40.8	170.67	18.33	1	6	6	6	371	5.31	0.001731
Q5A455	Protein transport protein SEC23	85.6	170.60	5.91	5	4	4	4	762	5.71	0.000562
P40954	Chitinase 3	60.0	170.48	4.23	1	1	1	2	567	4.91	0.000378
P46585	Ribose-phosphate pyrophosphokinase 1	35.3	169.12	15.89	1	4	4	5	321	6.79	0.001667
P78590	Elongation factor 1-beta	23.5	169.08	12.21	1	2	2	3	213	4.40	0.001507
Q5A1L6	Major glycerophosphoinositol permease GIT3	59.4	168.35	10.65	2	4	4	5	535	8.06	0.001000
Q59KG2	Respiratory growth induced protein 1	23.6	168.12	24.88	4	4	4	5	201	5.82	0.002662

Q9B8D8	Cytochrome c oxidase subunit 2	29.8	165.44	10.31	12	2	2	3	262	4.68	0.001225
P46250	SEC14 cytosolic factor	34.7	164.77	19.27	1	5	5	6	301	6.40	0.002133
Q5AFA2	Extracellular glycosidase CRH11	46.7	163.65	11.92	1	4	4	5	453	4.96	0.001181
Q5AJC0	Extracellular glycosidase UTR2	51.7	162.77	9.15	1	4	4	4	470	4.73	0.000911
P10977	Vacuolar aspartic protease	45.4	162.62	13.13	1	3	3	3	419	4.83	0.000766
P22274	ADP-ribosylation factor	20.2	161.07	20.11	1	1	2	3	179	5.40	0.001794
P30572	Chitin synthase 2	115.5	160.22	3.96	1	3	3	4	1009	5.73	0.000424
O74712	Histidine biosynthesis trifunctional protein	91.8	159.57	8.35	4	6	6	6	838	5.38	0.000766
Q59VR3	FK506-binding protein 3	47.6	159.53	13.38	1	5	5	5	426	4.46	0.001256
A0A1D8PCL1	High-affinity glucose transporter 1	60.6	159.27	9.36	1	5	5	5	545	7.62	0.000982
Q9HEW1	cAMP-dependent protein kinase regulatory subunit	50.3	157.41	8.28	1	3	3	3	459	5.48	0.000700
Q5AQ76	Protein transport protein SEC24	102.0	157.31	11.21	2	5	7	7	928	5.40	0.000807
Q5A1B0	Sterol-4-alpha-carboxylate 3-dehydrogenase ERG26, decarboxylating	39.2	156.09	11.43	1	3	3	3	350	6.73	0.000917
Q59ZV5	Eukaryotic translation initiation factor 3 subunit G	30.7	155.46	15.77	3	4	4	4	279	9.06	0.001534
P87078	DNA topoisomerase 2	165.3	154.89	4.79	2	6	6	6	1461	6.40	0.000440
Q5A762	Multiple drug resistance-associated protein-like transporter 1	180.6	153.63	4.55	1	7	7	7	1606	6.32	0.000466
Q59KI4	Chromatin-remodeling ATPase INO80	158.7	153.58	5.12	1	5	5	5	1387	8.09	0.000386
Q9HGY5	Negative regulator of the PHO system	37.3	153.37	13.80	6	4	4	4	326	6.86	0.001313
Q5A302	Endoplasmic reticulum vesicle protein 25	24.5	153.23	20.00	1	4	4	5	215	7.12	0.002489
C4YJI1	Altered inheritance of mitochondria protein 36, mitochondrial	34.7	152.00	19.18	3	5	5	5	292	8.34	0.001833
P30418	Glycylpeptide N-tetradecanoyltransferase	51.8	149.58	7.54	1	2	2	2	451	6.48	0.000475
P43065	Saccharopine dehydrogenase [NAD(+), L-lysine-forming]	42.4	149.20	11.52	1	3	3	4	382	5.44	0.001121
Q5AAR0	Transcription factor IWS1	45.8	148.82	15.00	1	5	5	5	400	8.12	0.001338
Q5A4P9	ATP-dependent RNA helicase DBP9	65.1	148.40	9.23	6	5	5	5	574	9.06	0.000932
P39827	Cell division control protein 10	40.7	146.65	9.24	1	2	2	2	357	7.11	0.000600
P46592	Glycolipid 2-alpha-mannosyltransferase 2	54.5	146.38	10.63	1	3	5	5	461	6.67	0.001161
P34725	Phospho-2-dehydro-3-deoxyheptonate aldolase, phenylalanine-inhibited	40.7	144.48	17.12	3	4	5	5	368	7.11	0.001454
P52498	Ras-related protein RSR1	27.6	143.18	18.95	2	4	4	4	248	5.21	0.001726
Q5AL27	Palmitoyltransferase AKR1	91.7	139.98	3.44	1	2	2	2	813	6.24	0.000263
Q5ACI8	Peptidyl-prolyl cis-trans isomerase D	40.7	139.90	16.26	2	3	4	4	369	6.39	0.001160
P87023	Beta-glucan synthesis-associated protein KRE6	82.4	139.52	6.89	1	4	4	6	740	4.78	0.000868
O42617	Poly(A) polymerase PAPalpha	63.2	136.93	7.17	3	3	3	3	558	8.07	0.000575

P22011	Peptidyl-prolyl cis-trans isomerase	17.6	135.85	19.14	2	2	3	4	162	7.97	0.002643
Q59MV9	Flavohemoprotein	45.8	134.36	13.82	1	3	3	4	398	5.83	0.001076
P42800	Inositol-3-phosphate synthase	57.7	133.67	13.27	1	5	5	5	520	5.54	0.001029
Q5ACM9	Eukaryotic translation initiation factor 3 subunit J	32.0	131.39	16.14	1	4	4	4	285	4.98	0.001502
Q59LF3	Regulator of cytoskeleton and endocytosis RVS167	49.2	130.95	8.64	1	3	3	4	440	6.02	0.000973
Q5AAI8	Nucleosome assembly protein 1	49.5	130.82	11.49	1	4	4	4	435	4.31	0.000984
Q59P03	NADH-cytochrome b5 reductase 1	32.5	130.12	20.41	2	5	5	5	294	8.10	0.001820
Q5AFE4	Regulator of cytoskeleton and endocytosis RVS161	30.1	128.27	20.45	1	5	5	5	264	7.14	0.002027
P83773	Acetyl-CoA hydrolase	58.0	125.23	6.30	2	3	3	3	524	6.92	0.000613
Q59KM8	Cell cycle protein kinase DBF2	82.3	119.66	3.10	1	2	2	3	710	9.13	0.000452
Q59VX8	Septation protein 7	75.7	117.54	4.78	1	3	3	4	670	6.02	0.000639
A0A1D8PNZ7	Glycerophosphocholine phosphodiesterase GDE1	130.8	117.54	2.41	1	2	2	2	1162	6.30	0.000184
Q00313	DNA topoisomerase 1	90.4	114.73	4.37	1	2	3	3	778	9.03	0.000413
O94150	37S ribosomal protein S9, mitochondrial	38.6	114.30	9.52	1	3	3	3	336	10.26	0.000956
Q9HFQ6	60S acidic ribosomal protein P1-B	10.7	113.93	14.81	1	1	1	1	108	3.93	0.000991
Q5AG77	Amino-acid permease GAP1	63.9	113.72	2.75	1	1	1	1	582	9.17	0.000184
O74226	Cell wall synthesis protein KRE9	29.1	113.06	10.33	1	2	2	2	271	8.18	0.000790
A0A1D8PLI2	Isopentenyl-diphosphate delta-isomerase	32.3	112.45	15.14	1	3	3	3	284	4.97	0.001131
O94072	V-type proton ATPase subunit E	25.4	112.41	21.24	2	4	4	4	226	5.40	0.001894
O42816	Signal recognition particle 54 kDa protein homolog	60.7	112.38	8.27	2	4	4	4	556	9.13	0.000770
Q5AP65	Protein FMP52, mitochondrial	24.3	112.37	13.10	1	3	3	3	229	8.98	0.001402
Q5AC48	Actin-related protein 4	52.6	111.69	9.19	1	3	3	4	468	5.57	0.000915
Q5ANP2	Nascent polypeptide-associated complex subunit alpha	19.5	110.00	14.04	3	2	2	2	178	4.82	0.001203
O42779	Candidapepsin-9	58.6	108.48	6.25	2	3	3	3	544	5.25	0.000590
Q9P844	40S ribosomal protein S21	9.6	104.55	28.74	2	2	2	2	87	8.15	0.002460
Q92207	Mitogen-activated protein kinase HOG1	42.9	104.43	8.49	4	3	3	3	377	5.19	0.000852
Q59WG0	Adenosine 5'-monophosphoramidase HNT1	17.0	103.83	23.68	1	2	2	2	152	6.86	0.001408
Q9UVL1	Non-histone chromosomal protein 6	10.5	103.04	28.26	2	2	2	3	92	9.70	0.003490
Q5ACK7	ATP-dependent RNA helicase DRS1	69.3	102.97	5.71	2	3	3	3	613	6.19	0.000524
Q8J0Q0	Mannosyl-oligosaccharide 1,2-alpha-mannosidase	64.6	102.88	7.08	1	4	4	4	565	5.11	0.000758
Q9Y7C4	ATP-dependent RNA helicase CHR1	65.4	101.69	7.09	5	3	3	3	578	9.23	0.000555
Q92209	Homoserine kinase	39.1	100.55	11.20	1	3	3	3	357	5.67	0.000899

O14427	Serine/threonine-protein kinase CLA4	106.8	100.39	3.50	2	3	3	3	971	9.35	0.000331
Q5AFT3	Protein CFT1	161.8	100.33	1.62	1	1	1	2	1420	5.21	0.000151
Q5A761	CCR4-Not complex 3'-5'-exoribonuclease subunit Ccr4	90.3	99.82	5.46	2	4	4	4	787	7.20	0.000544
Q5AED9	Branchpoint-bridging protein	50.0	99.23	5.93	1	2	2	2	455	9.42	0.000470
Q5AP66	Phosphatidylinositol transfer protein SFH5	36.6	97.68	9.69	1	2	2	2	320	5.33	0.000669
P34948	Mannose-6-phosphate isomerase	48.8	97.28	9.98	1	4	4	4	441	5.33	0.000971
Q5A309	Histone-lysine N-methyltransferase, H3 lysine-79 specific	155.6	96.91	1.94	1	2	2	2	1343	6.38	0.000159
P87024	Beta-glucan synthesis-associated protein SKN1	83.7	96.22	4.48	1	2	2	2	737	5.15	0.000290
P53697	Mannan polymerase complex subunit MNN9	42.8	96.09	5.98	1	2	2	3	368	9.14	0.000872
A0A1D8PC43	Diphosphomevalonate decarboxylase	39.5	96.07	9.12	1	3	3	3	362	6.46	0.000887
A0A1D8PEL1	Mevalonate kinase	47.0	94.31	7.19	1	2	2	2	431	5.68	0.000497
G1UB11	C-22 sterol desaturase ERG5	59.6	94.24	8.12	1	4	4	4	517	6.64	0.000828
Q5ADW3	Cullin-associated NEDD8-dissociated protein 1	134.4	93.17	5.44	1	5	5	5	1195	4.96	0.000448
Q5AGM0	Protein HIR2	114.4	91.69	2.26	1	2	2	2	1017	8.12	0.000210
P46588	DNA polymerase delta catalytic subunit	118.8	90.78	2.60	1	2	2	2	1038	8.21	0.000206
P53705	Bud site selection protein BUD4	192.8	88.55	1.11	1	1	1	1	1709	6.04	0.000063
Q59NP1	Copper transport protein CTR1	27.8	88.06	17.93	1	2	2	2	251	6.93	0.000853
Q5APC0	Golgi apparatus membrane protein TVP18	18.8	87.93	10.98	1	1	1	1	173	7.74	0.000619
P0CY33	Cell division control protein 42 homolog	21.2	86.92	18.85	1	3	3	3	191	6.54	0.001681
Q5A4Q1	Adenylate kinase	27.6	85.86	10.44	8	3	3	3	249	8.10	0.001289
C4YH95	tRNA (guanine(37)-N1)-methyltransferase	51.8	85.85	11.58	2	4	4	4	449	8.02	0.000953
Q92210	Phosphoribosylaminoimidazole carboxylase	62.4	85.41	5.63	1	3	3	3	568	6.49	0.000565
Q59WH0	Transcriptional adapter 2	51.1	85.39	5.39	1	2	2	2	445	7.46	0.000481
Q5AL52	Formin BNI1	196.7	83.80	1.62	1	2	2	2	1732	6.15	0.000124
Q5A6Q4	tRNA (adenine(58)-N(1))-methyltransferase non-catalytic subunit TRM6	51.2	82.72	3.53	1	1	1	1	453	6.98	0.000236
Q874I4	Dihydroorotate dehydrogenase (quinone), mitochondrial	48.4	82.66	5.86	3	2	2	2	444	9.20	0.000482
P43068	Mitogen-activated protein kinase MKC1	58.2	81.05	3.99	2	1	1	2	501	5.02	0.000427
Q9UW14	pH-response transcription factor pacC/RIM101	74.6	79.57	3.18	28	2	2	2	661	7.06	0.000324
Q59KJ7	Alpha-1,2-mannosyltransferase MNN21	76.9	79.29	2.12	1	1	1	1	660	7.11	0.000162
Q5ALL8	FACT complex subunit POB3	60.9	79.07	5.20	1	2	2	2	538	4.83	0.000398
Q5A2J7	Calcium channel YVC1	77.3	79.04	3.41	1	2	2	2	675	5.08	0.000317
Q59X67	Enhanced filamentous growth protein 1	59.6	78.68	3.64	4	2	2	2	550	9.36	0.000389

Q9P8E3	Protein transport protein SEC61 subunit alpha	52.5	78.62	8.14	2	3	3	4	479	8.95	0.000894
Q5AEF2	Protein transport protein SEC13	33.0	77.68	8.05	1	2	2	2	298	5.62	0.000718
A0A1D8PD39	3-hydroxy-3-methylglutaryl-coenzyme A reductase 1	116.4	77.24	2.89	3	3	3	3	1073	7.33	0.000299
Q5AQ57	37S ribosomal protein S25, mitochondrial	34.2	76.59	8.14	1	2	2	2	295	8.03	0.000726
P0CB54	ATPase GET3	39.6	76.57	10.00	5	3	3	3	350	5.02	0.000917
Q59W44	Mitochondrial import inner membrane translocase subunit TIM50	54.2	74.77	6.61	2	3	3	3	469	6.65	0.000685
P13649	Orotidine 5'-phosphate decarboxylase	29.9	74.53	9.26	4	2	2	2	270	5.76	0.000793
Q5AP53	Serine/threonine-protein kinase CBK1	84.4	74.49	5.05	1	3	3	3	732	6.40	0.000439
C4YS65	Protein SEY1	90.2	74.46	2.41	2	1	1	1	790	5.15	0.000135
Q5AH60	tRNA (guanine-N(7)-)-methyltransferase non-catalytic subunit TRM82	49.1	74.06	4.69	1	1	1	1	426	5.26	0.000251
P46586	ATP phosphoribosyltransferase	32.6	73.84	5.03	1	1	1	1	298	5.34	0.000359
Q5ABP8	Protein ROT1	29.9	72.89	5.38	1	1	1	1	260	7.80	0.000412
Q9UWF6	Lysophospholipase 1	66.4	72.74	2.15	1	1	1	1	605	4.87	0.000177
P87185	Cysteine desulfurase, mitochondrial	53.5	72.08	8.40	4	3	3	4	488	8.18	0.000877
P53716	Uncharacterized protein CAWG_04269, mitochondrial	25.0	71.90	10.76	1	2	2	2	223	8.63	0.000960
Q5ADM9	Dolichyl-phosphate-mannose--protein mannosyltransferase 2	88.3	71.34	2.34	1	2	2	2	769	7.21	0.000278
Q5AJC1	Vacuolar protein-sorting protein BRO1	108.8	70.72	1.38	1	1	1	1	945	5.52	0.000113
O93831	Rab proteins geranylgeranyltransferase component A	72.2	70.33	7.34	1	3	3	3	640	4.81	0.000502
Q5AD56	General negative regulator of transcription subunit 3	85.7	69.33	5.19	1	2	2	2	752	5.57	0.000285
Q59PE7	Protein BCP1	37.4	69.09	3.12	1	1	1	1	321	4.77	0.000333
Q59S59	ATP-dependent RNA helicase MRH4, mitochondrial	62.5	68.58	2.88	1	1	1	1	555	9.70	0.000193
Q00314	Vanadate resistance protein	50.7	68.07	5.88	2	2	2	2	442	5.41	0.000484
A0A1D8PL26	2-(3-amino-3-carboxypropyl)histidine synthase subunit 2-2	59.1	68.01	2.65	2	1	1	1	529	5.73	0.000202
Q5AI21	Translocation protein SEC62	33.3	66.63	11.95	1	3	3	3	293	9.51	0.001096
C4YHS3	RNA polymerase II degradation factor 1	54.1	66.30	3.46	2	1	1	1	492	4.97	0.000218
Q59V93	Very-long-chain 3-oxoacyl-CoA reductase	38.3	66.25	3.15	1	1	1	1	349	9.57	0.000307
Q59PR3	ATP-dependent RNA helicase DBP8	48.8	65.98	4.32	13	1	2	2	440	8.91	0.000486
Q59PD6	Spindle assembly checkpoint component MAD1	82.2	64.85	2.30	1	1	1	1	696	6.93	0.000154
Q9P4V2	Phosphoacetylglucosamine mutase	60.4	64.73	4.60	1	2	2	2	544	5.39	0.000393
P43102	Ubiquitin-conjugating enzyme E2 4	16.3	64.56	12.24	6	2	2	2	147	7.40	0.001456
P0CU36	Ribosome biogenesis protein C3_06160C_A	29.6	64.33	12.26	7	3	3	3	261	10.18	0.001230
Q5A9Z6	ATP-dependent RNA helicase FAL1	45.5	64.05	3.26	1	1	1	1	399	8.02	0.000268

Q59X23	Dolichyl-phosphate-mannose--protein mannosyltransferase 4	86.6	62.86	2.25	1	2	2	2	755	8.87	0.000284
Q59S72	GDP-Man:Man(3)GlcNAc(2)-PP-Dol alpha-1,2-mannosyltransferase	70.9	61.40	2.30	1	1	1	1	609	8.53	0.000176
O13427	Low-specificity L-threonine aldolase	41.8	61.03	4.01	1	1	1	1	374	6.42	0.000286
O74270	Origin recognition complex subunit 1	91.2	59.96	1.61	1	1	1	1	805	6.25	0.000133
Q5AF95	ATP-dependent RNA helicase DBP4	86.7	59.58	3.27	1	2	2	2	765	8.15	0.000280
O13359	Kexin	105.1	58.60	2.35	1	2	2	2	938	5.03	0.000228
Q5AG40	Vacuolar protein sorting-associated protein 4	48.4	58.17	2.51	1	1	1	1	439	5.64	0.000244
O93875	Delta(7)-sterol 5(6)-desaturase	45.4	57.81	5.18	3	2	2	2	386	6.80	0.000555
Q5A310	ISWI chromatin-remodeling complex ATPase ISW2	123.0	57.60	2.08	1	2	2	2	1056	6.73	0.000203
C4YKP5	Increased recombination centers protein 22-1	25.9	57.01	22.18	4	3	3	3	239	5.68	0.001343
Q5A368	Lysophospholipase NTE1	155.6	55.80	1.95	1	3	3	3	1386	8.38	0.000232
Q5AHG6	Serine/threonine-protein kinase SCH9	88.7	55.67	1.27	1	1	1	1	787	6.20	0.000136
Q5A119	Chromatin modification-related protein EAF1	79.5	54.92	1.90	1	1	1	1	686	9.76	0.000156
Q5ABG1	Histone-lysine N-methyltransferase, H3 lysine-4 specific	119.1	54.58	1.44	1	1	1	1	1040	7.75	0.000103
Q5AI97	Mitochondrial genome maintenance protein MGM101	30.7	53.82	5.45	2	1	1	1	275	9.10	0.000389
Q5AD77	Sorting nexin-4	71.8	53.51	2.06	1	1	1	1	630	5.63	0.000170
Q59WF4	Alpha-1,2-mannosyltransferase MNN2	69.1	53.10	4.02	1	2	2	2	597	6.27	0.000359
P52499	Protein RCC1	51.3	53.07	4.74	1	2	2	2	464	5.43	0.000461
Q5A6P6	MFS antiporter QDR1	55.4	53.02	2.16	1	1	1	1	509	8.85	0.000210
Q5AJS6	Multiple RNA-binding domain-containing protein 1	94.9	51.86	1.55	1	1	1	1	841	5.62	0.000127
Q96WL3	Protein URE2	39.1	51.58	3.49	1	1	1	1	344	5.94	0.000311
Q5A3M6	GPI ethanolamine phosphate transferase 1	110.6	50.98	0.93	1	1	1	1	971	5.96	0.000110
P53698	Cytochrome c	12.2	50.19	25.45	13	3	3	4	110	9.66	0.003892
Q5APM7	ATP-dependent RNA helicase MSS116, mitochondrial	76.7	50.03	4.04	1	2	2	2	668	9.48	0.000320
Q5AHB1	Actin cytoskeleton-regulatory complex protein PAN1	152.3	49.77	1.22	1	1	1	1	1396	6.23	0.000077
Q9HFQ7	60S acidic ribosomal protein P1-A	11.0	49.75	6.60	1	1	1	1	106	4.02	0.001010
Q59XM1	Exocyst complex component EXO84	88.5	49.60	2.15	1	1	1	1	791	8.98	0.000135
Q59JU3	Pre-rRNA-processing protein IPI1-1	41.0	49.39	3.83	1	1	1	1	366	9.70	0.000292
Q5AD78	Mannan endo-1,6-alpha-mannosidase DCW1	50.3	49.21	6.19	1	2	2	2	452	4.73	0.000474
Q5AKU3	CAP1-binding-protein	73.9	48.39	1.87	1	1	1	1	643	5.92	0.000166
Q5AA50	Nuclear protein localization protein 4	67.3	48.17	2.34	1	1	1	1	598	6.29	0.000179
O93803	mRNA-capping enzyme subunit beta	58.8	47.60	2.12	1	1	1	1	520	7.87	0.000206

Q59NY7	Coupling of ubiquitin conjugation to ER degradation protein 1	21.2	47.52	5.29	1	1	1	1	189	7.15	0.000566
O14415	Signal recognition particle SEC65 subunit	31.1	46.74	4.00	1	1	1	1	275	7.53	0.000389
Q59VP2	Histone H2A.2	13.8	46.43	23.66	45	1	2	2	131	10.24	0.001634
Q00312	Transcription factor RBF1	59.4	46.27	1.71	2	1	1	1	527	8.66	0.000203
P56091	Galactokinase	57.0	45.86	1.75	1	1	1	1	515	5.54	0.000208
P23286	Calmodulin	16.5	45.61	20.13	9	2	2	2	149	4.41	0.001437
P53699	Cell division control protein 4	76.0	45.56	3.36	1	2	2	2	684	5.95	0.000313
Q59Y46	Morphogenetic regulator of filamentous growth protein 1	83.4	44.90	1.06	1	1	1	1	758	10.17	0.000141
Q59Y41	DNA mismatch repair protein MSH3	118.8	44.51	0.96	1	1	1	1	1037	7.52	0.000103
Q59NX9	Diphthine methyl ester synthase 1	33.9	43.37	9.36	1	3	3	3	299	4.87	0.001074
Q5AEK8	Delta 8-(E)-sphingolipid desaturase	67.3	42.54	1.37	1	1	1	1	584	6.96	0.000183
Q59XQ1	3'(2'),5'-bisphosphate nucleotidase 2	38.7	42.14	4.19	1	1	1	1	358	5.86	0.000299
Q5A2A2	Mitochondrial homologous recombination protein 1	28.3	42.06	4.60	1	1	1	1	239	8.50	0.000448
P31225	Corticosteroid-binding protein	55.5	41.73	2.04	1	1	1	1	489	5.29	0.000219
Q5AK62	Virulence protein SSD1	141.2	41.48	1.65	1	2	2	2	1274	7.36	0.000168
Q59Q38	Peptide-N(4)-(N-acetyl-beta-glucosaminyl)asparagine amidase	45.8	41.31	5.06	1	2	2	2	395	7.18	0.000542
Q5A1Q5	Probable kinetochore protein NUF2	56.6	41.31	1.86	1	1	1	1	485	5.55	0.000221
Q12572	L-2-aminoadipate reductase large subunit	154.6	40.87	1.80	1	3	3	3	1391	6.87	0.000231
A0A1D8PN88	Amino-acid permease GAP3	65.5	40.24	1.84	1	1	1	1	599	8.43	0.000179
Q5A360	Nucleotide exchange factor SIL1	47.0	39.52	3.62	1	1	1	1	414	5.07	0.000259
Q5A3V6	3,4-dihydroxy-2-butanone 4-phosphate synthase	22.6	39.07	3.43	1	1	1	1	204	5.44	0.000525
Q5AB93	Protein PNS1	58.0	38.85	1.16	1	1	1	1	518	8.46	0.000207
Q59Z14	Deoxyhypusine hydroxylase	35.8	37.28	9.12	1	2	2	2	318	4.74	0.000673
Q3MNT0	Transcription elongation factor SPT6	162.0	36.89	1.14	1	1	1	1	1401	5.24	0.000076
Q59RP7	54S ribosomal protein L4, mitochondrial	36.3	36.35	2.59	1	1	1	1	309	5.96	0.000346
Q59PP6	Mediator of RNA polymerase II transcription subunit 16	117.7	35.97	0.85	1	1	1	1	1053	7.61	0.000102
Q59Y20	Protein DSE1	81.0	35.85	2.49	1	1	1	1	724	5.94	0.000148
Q5AHJ5	Transcription factor TBF1	100.8	35.73	1.13	1	1	1	1	886	4.67	0.000121
Q5ABD0	Vacuolar-sorting protein SNF7	25.9	35.44	5.31	1	1	1	1	226	4.88	0.000474
Q5A8X7	Protein FYV4, mitochondrial	19.8	35.37	5.29	1	1	1	1	170	9.99	0.000630
Q59PP0	DNA mismatch repair protein HSM3	55.4	35.01	1.69	1	1	1	1	472	5.25	0.000227
Q9P836	60S ribosomal protein L37 (Fragment)	9.9	33.63	7.95	1	1	1	1	88	11.63	0.001216

Q5AM44	mRNA 3'-end-processing protein RNA14	91.6	32.70	1.26	1	1	1	1	791	5.21	0.000135
P87163	Translation initiation factor eIF-2B subunit epsilon	81.9	32.25	1.64	1	1	1	1	732	4.65	0.000146
Q59U67	Transcription initiation factor TFIID subunit 4	39.6	31.77	2.75	1	1	1	1	363	9.13	0.000295
C4YJ78	Methylthioribose-1-phosphate isomerase	46.3	31.71	2.88	2	1	1	1	416	5.33	0.000257
Q59LF2	Alpha-1,3/1,6-mannosyltransferase ALG2	50.4	31.66	2.26	1	1	1	1	443	8.31	0.000242
C4YF50	NAD(P)H-hydrate epimerase	28.7	31.19	6.59	2	1	1	1	258	6.70	0.000415
Q9UVX1	Lysophospholipase 3	81.4	30.99	1.06	1	1	1	1	754	4.84	0.000142
Q5A0W6	GPN-loop GTPase 3	30.8	30.63	8.79	1	2	2	2	273	4.50	0.000784
C4YS59	Vacuolar membrane protease	93.7	30.29	1.79	2	1	1	1	837	5.29	0.000128
Q5A2K0	Stress response protein NST1	158.4	30.04	0.71	1	1	1	1	1399	5.96	0.000077
Q92410	Alpha,alpha-trehalose-phosphate synthase [UDP-forming]	54.4	29.86	1.46	1	1	1	1	478	6.23	0.000224
Q59S50	ATP-dependent RNA helicase DBP7	81.1	29.59	1.24	3	1	1	1	727	8.95	0.000147
Q5AEM8	Presequence translocated-associated motor subunit PAM17, mitochondrial	21.0	29.27	10.81	1	1	1	1	185	10.14	0.000579
Q5A287	Transcription factor SFL1	90.1	28.71	2.24	1	1	1	1	805	9.50	0.000133
Q5A7S7	Fork-head transcriptional regulator 2	76.3	28.36	1.02	2	1	1	1	687	9.42	0.000156
C4YTL7	Cytochrome c oxidase assembly factor 3, mitochondrial	10.1	28.33	23.86	1	1	1	1	88	8.97	0.001216
Q5A519	EKC/KEOPS complex subunit CGI121	23.4	28.29	8.87	1	1	1	1	203	5.97	0.000527
Q5A599	Histidine protein kinase NIK1	118.9	27.18	2.78	1	2	2	2	1081	5.80	0.000198
Q5A4W8	Bromodomain-containing factor 1	82.5	27.07	1.23	1	1	1	1	732	5.02	0.000146
A0A1D8PJ25	Delta(24(24(1)))-sterol reductase	54.8	26.99	2.13	1	1	1	1	469	7.06	0.000228
C4YTG0	Pheromone-processing carboxypeptidase KEX1	78.7	26.12	1.28	2	1	1	1	702	5.12	0.000152
A0A1D8PNP3	Amino-acid permease GAP6	61.8	24.72	1.58	1	1	1	1	568	8.72	0.000188
Q5A3P6	Serine/threonine-protein kinase PKH2	106.5	24.36	1.80	1	1	1	1	947	8.28	0.000113
Q5ADL9	Defective in cullin neddylation protein 1	34.9	24.22	3.38	1	1	1	1	296	5.05	0.000362
Q59U81	Helicase SWR1	187.6	23.62	0.55	1	1	1	1	1641	5.58	0.000065
P0C8K9	Cytochrome c oxidase subunit 1	58.7	22.18	5.08	1	1	1	1	531	6.37	0.000202
Q5ACL9	Protein BFR2	58.4	21.69	1.95	1	1	1	1	512	4.74	0.000209
Q5A416	tRNA (adenine(58)-N(1))-methyltransferase catalytic subunit TRM61	38.0	20.71	2.69	1	1	1	1	335	7.55	0.000319
**EV_{AMB}**											
Q92211	Glyceraldehyde-3-phosphate dehydrogenase	35.8	10731.85	86.27	41	21	26	347	335	7.12	0.101500
P83779	Pyruvate decarboxylase	62.4	5642.11	62.96	8	27	27	149	567	5.58	0.025751
P41797	Heat shock protein SSA1	70.3	4816.14	50.61	9	7	25	144	656	5.17	0.021510

P46587	Heat shock protein SSA2	70.0	4806.99	63.41	7	15	30	147	645	5.06	0.022333
P25997	Elongation factor 3	116.9	4569.73	54.76	6	39	44	122	1050	5.73	0.011386
P0CY35	Elongation factor 1-alpha 1	50.0	3434.48	50.00	21	6	21	195	458	9.03	0.041721
P43067	Alcohol dehydrogenase 1	36.9	3376.88	60.00	3	14	17	111	350	6.44	0.031077
P87222	Heat shock protein SSB1	66.4	2961.42	59.54	9	26	26	80	613	5.38	0.012788
Q9URB4	Fructose-bisphosphate aldolase	39.2	2945.25	70.19	3	18	18	78	359	6.06	0.021290
P28877	Plasma membrane ATPase 1	97.4	2830.42	38.55	7	17	26	75	895	4.96	0.008211
P34731	Fatty acid synthase subunit beta	227.8	2221.07	31.08	3	46	46	60	2037	6.02	0.002886
C4YJQ8	Elongation factor 2	93.3	2032.73	49.52	13	36	36	64	842	6.47	0.007448
P43098	Fatty acid synthase subunit alpha	207.5	1931.53	24.77	9	37	37	49	1885	5.62	0.002547
Q96VB9	Heat shock protein homolog SSE1	78.5	1909.98	61.34	7	33	33	49	701	5.30	0.006850
P14235	Actin	41.7	1845.44	50.53	30	15	15	51	376	5.69	0.013291
P83776	Hexokinase-2	53.4	1836.00	65.91	3	21	23	54	484	5.55	0.010933
P83784	Heat shock protein SSC1, mitochondrial	69.7	1455.55	47.38	3	24	24	34	648	5.60	0.005141
P43076	pH-responsive protein 1	59.4	1278.09	43.25	2	20	21	29	548	5.07	0.005186
Q9Y872	Sulfate adenylyltransferase	58.8	1271.33	51.61	20	22	22	32	527	6.40	0.005950
P30575	Enolase 1	47.2	1261.80	49.77	11	15	15	27	440	5.81	0.006013
O13434	Phosphoenolpyruvate carboxykinase [ATP]	60.8	1183.63	47.74	1	19	19	29	553	6.60	0.005139
P83778	Malate dehydrogenase, cytoplasmic	36.0	1039.99	57.57	1	14	14	21	337	5.62	0.006106
P46598	Heat shock protein 90 homolog	80.8	1026.29	39.46	7	23	23	27	707	4.88	0.003742
O94039	Transketolase 1	73.7	1017.82	38.55	1	19	19	26	677	5.82	0.003763
P46273	Phosphoglycerate kinase	45.2	978.17	61.15	21	21	21	35	417	6.48	0.008225
P43071	Multidrug resistance protein CDR1	169.8	952.83	17.52	6	18	20	25	1501	6.98	0.001632
Q59PT0	V-type proton ATPase subunit B	57.2	948.22	52.93	5	2	22	25	512	5.03	0.004785
O74261	Heat shock protein 60, mitochondrial	60.1	945.66	35.34	5	15	15	20	566	5.30	0.003463
O74676	ABC transporter CDR4	169.2	925.27	19.13	5	20	22	23	1490	7.65	0.001513
O94049	Acetyl-coenzyme A synthetase 1	75.1	887.64	43.41	6	22	23	32	675	6.32	0.004645
Q9HGT6	Serine--tRNA ligase, cytoplasmic	53.0	870.63	51.08	4	17	17	24	462	5.73	0.005090
P82610	5-methyltetrahydropteroyltriglutamate--homocysteine methyltransferase	85.6	847.88	28.81	2	15	15	19	767	5.60	0.002427
P46614	Pyruvate kinase	55.4	825.07	36.11	14	12	12	16	504	6.99	0.003111
Q5A8K2	Alanine--tRNA ligase	108.2	822.42	29.62	3	24	24	25	969	6.07	0.002528
P40910	40S ribosomal protein S1	29.0	815.02	57.42	53	15	15	23	256	10.04	0.008804

O42817	40S ribosomal protein S0	28.7	804.51	61.30	33	12	12	27	261	4.91	0.010137
P83781	Mitochondrial outer membrane protein porin	29.7	761.65	54.96	1	13	13	20	282	8.57	0.006950
P83782	Cytochrome b-c1 complex subunit 2, mitochondrial	39.5	757.93	75.40	1	17	17	21	374	5.57	0.005502
P30573	Chitin synthase 3	136.1	752.21	18.38	9	16	16	20	1213	7.17	0.001616
Q96UX5	Heat shock protein 78, mitochondrial	91.6	748.53	24.38	2	13	13	16	812	6.80	0.001931
P83774	Guanine nucleotide-binding protein subunit beta-like protein	34.5	746.69	48.58	1	10	10	19	317	6.54	0.005873
P82611	Aconitate hydratase, mitochondrial	84.2	741.15	26.38	2	15	15	19	777	6.39	0.002396
Q9P843	60S ribosomal protein L27	15.5	732.00	47.06	1	6	6	23	136	10.18	0.016572
Q8NJN3	Acetyl-coenzyme A synthetase 2	73.8	730.22	29.96	13	18	19	25	671	6.13	0.003651
Q59KZ1	Aminopeptidase 2	104.3	718.36	25.54	2	20	20	21	924	5.36	0.002227
Q5AML1	Eukaryotic translation initiation factor 3 subunit C	99.8	702.09	23.23	5	18	18	19	874	5.15	0.002130
P47837	40S ribosomal protein S4	29.2	682.61	43.89	1	8	11	21	262	10.21	0.007854
O94038	Alcohol dehydrogenase 2	36.8	681.53	45.40	8	9	13	17	348	6.68	0.004787
P53707	37 kDa cell surface protein	37.0	678.99	41.74	1	12	12	18	321	7.06	0.005495
Q5AKA5	Cys-Gly metallodipeptidase DUG1	53.6	659.11	35.46	1	11	11	16	485	5.24	0.003233
Q5AJB1	V-type proton ATPase catalytic subunit A	67.6	657.04	25.12	7	12	12	15	617	5.27	0.002382
O13287	6-phosphogluconate dehydrogenase, decarboxylating	56.9	651.29	30.56	4	13	13	17	517	6.57	0.003222
Q59PR9	Transcriptional regulator HMO1	24.8	634.87	47.09	1	6	6	18	223	9.19	0.007910
P52495	Ubiquitin-activating enzyme E1 1	114.2	608.30	21.45	2	15	15	17	1021	5.01	0.001632
O94083	Eukaryotic translation initiation factor 5A	17.1	604.71	52.53	6	7	7	17	158	5.05	0.010543
P83777	Inorganic pyrophosphatase	32.1	600.21	50.35	12	12	12	15	288	5.26	0.005104
P47828	T-complex protein 1 subunit theta	58.9	595.10	32.96	2	13	13	15	540	7.97	0.002722
Q9UVJ4	60S ribosomal protein L10a	24.4	588.59	39.17	4	9	9	16	217	9.76	0.007225
Q5AHH4	Small heat shock protein 21	21.5	582.93	58.20	1	9	9	22	189	5.35	0.011406
O42825	GTP-binding protein RHO1	22.0	580.67	52.02	7	8	8	12	198	5.73	0.005939
O93827	Mannose-1-phosphate guanyltransferase	40.0	580.03	57.18	15	17	17	20	362	6.30	0.005414
Q00310	Glycolipid 2-alpha-mannosyltransferase 1	50.0	563.77	37.35	1	11	13	15	431	6.90	0.003410
P10613	Lanosterol 14-alpha demethylase	60.6	546.51	31.06	5	15	15	16	528	7.17	0.002969
Q96W54	40S ribosomal protein S22	14.8	541.56	58.46	9	6	6	11	130	9.88	0.008291
Q59KI0	UTP--glucose-1-phosphate uridylyltransferase	55.5	536.22	24.60	3	9	9	11	500	6.73	0.002156
P43057	Protein kinase C-like 1	125.2	535.71	13.95	1	11	11	11	1097	7.42	0.000983
Q5A4E2	ATP-dependent RNA helicase DED1	72.8	531.71	30.06	56	13	15	18	672	8.28	0.002625

O94200	ATP-dependent 6-phosphofructokinase subunit beta	104.0	530.66	18.29	3	14	15	17	946	6.35	0.001761
P87206	ATP-dependent RNA helicase eIF4A	44.6	527.55	45.34	40	14	14	18	397	5.36	0.004443
Q5AJD0	ATP-dependent RNA helicase DBP5	60.2	525.53	31.67	10	13	13	14	540	5.76	0.002540
O13318	pH-responsive protein 2	58.7	522.31	18.38	1	8	9	11	544	4.64	0.001981
Q9P8Q7	Isocitrate lyase	61.4	519.72	33.27	17	14	14	16	550	7.11	0.002851
Q59S78	Small COPII coat GTPase SAR1	21.5	509.68	57.37	17	8	8	11	190	5.59	0.005673
P83773	Acetyl-CoA hydrolase	58.0	503.56	44.27	3	16	16	18	524	6.92	0.003366
Q5APD4	Sphingolipid C9-methyltransferase	58.7	492.57	22.03	2	11	11	12	513	6.92	0.002292
O13354	Eukaryotic peptide chain release factor GTP-binding subunit	79.0	483.49	20.28	6	9	9	10	715	8.47	0.001370
Q5AEN1	Cytochrome c peroxidase, mitochondrial	40.7	480.14	37.43	1	10	10	15	366	6.34	0.004016
A0A1D8PCL1	High-affinity glucose transporter 1	60.6	477.30	22.02	1	10	10	13	545	7.62	0.002337
O13289	Peroxisomal catalase	54.8	474.68	25.36	2	10	10	12	485	6.65	0.002424
O74198	Sterol 24-C-methyltransferase	43.0	472.85	42.02	3	12	12	14	376	6.11	0.003649
O94201	ATP-dependent 6-phosphofructokinase subunit alpha	108.5	461.47	21.68	3	18	19	20	987	6.62	0.001986
Q5AJY5	1,3-beta-glucanosyltransferase PGA4	49.0	461.22	21.06	1	7	7	10	451	4.74	0.002173
C4YKT4	Ras-like protein 1	32.2	458.53	28.47	2	5	5	8	288	4.67	0.002722
O42766	14-3-3 protein homolog	29.5	458.18	39.39	8	7	7	11	264	4.81	0.004083
Q59M70	NADH-cytochrome b5 reductase 2	33.4	457.69	46.84	3	12	12	15	301	8.48	0.004883
Q96W53	40S ribosomal protein S14	14.4	456.77	46.27	4	6	6	11	134	10.61	0.008044
Q5ALX3	Transcription elongation factor SPT5	105.8	448.45	12.55	1	8	8	9	956	5.16	0.000923
Q5A860	Translationally-controlled tumor protein homolog	18.5	446.62	42.51	2	4	4	6	167	4.46	0.003521
O13425	Serine hydroxymethyltransferase, mitochondrial	54.5	443.83	23.53	5	9	9	12	493	8.97	0.002385
Q5AI37	Probable metalloprotease ARX1	62.2	440.35	21.63	2	10	10	10	564	6.92	0.001737
Q5AGV4	Eukaryotic translation initiation factor 3 subunit B	84.2	436.35	22.06	5	12	12	14	739	6.39	0.001856
Q5AME2	Pentafunctional AROM polypeptide	169.3	432.58	8.64	5	11	11	11	1551	6.58	0.000695
Q9P940	Triosephosphate isomerase	26.6	425.35	51.61	4	11	11	14	248	6.01	0.005532
Q5APF2	GMP synthase [glutamine-hydrolyzing]	58.8	423.80	29.81	7	12	12	14	530	5.92	0.002588
Q5AAU3	Protein transport protein SEC31	136.2	417.39	11.86	4	10	10	13	1265	6.77	0.001007
Q59ZX6	U3 small nucleolar RNA-associated protein 10	204.2	410.72	6.66	5	9	9	10	1818	5.87	0.000539
P87066	Tubulin alpha chain	49.9	390.75	29.69	15	10	10	12	448	5.06	0.002625
Q59MQ0	Myosin-5	146.9	390.30	9.12	20	10	10	10	1316	9.35	0.000745
Q5AIR7	Endo-1,3(4)-beta-glucanase 1	124.0	389.49	11.27	1	10	10	11	1145	5.40	0.000941

P39826	Cell division control protein 3	47.8	388.84	21.88	3	7	7	8	416	6.64	0.001884
Q5A4M8	Protein SUR7	29.9	383.41	32.22	1	5	5	7	270	7.69	0.002540
Q92206	Squalene monooxygenase	55.3	379.13	18.75	1	8	8	11	496	8.73	0.002173
P34732	Vesicular-fusion protein SEC18	88.9	378.90	19.14	3	10	11	11	794	7.43	0.001358
Q59LU0	ATP-dependent RNA helicase DBP2	61.2	375.50	32.38	31	14	15	18	562	8.88	0.003138
Q5AF03	Glyoxalase 3	25.8	374.66	37.29	1	5	5	8	236	4.83	0.003322
P10875	Tubulin beta chain	49.9	372.48	26.73	9	6	9	11	449	4.74	0.002401
P53704	Glutamine--fructose-6-phosphate aminotransferase [isomerizing]	79.2	364.81	19.50	3	10	10	11	713	6.24	0.001512
A0A1D8PN12	Glycerophosphodiester transporter GIT2	59.3	363.64	16.48	1	7	7	9	534	8.10	0.001652
Q8TGH6	Guanosine-diphosphatase	65.9	359.03	18.86	1	8	8	9	599	5.94	0.001472
P43084	Probable NADPH dehydrogenase	46.0	355.52	32.43	2	11	11	13	407	6.39	0.003130
O59931	60S ribosomal protein L13	23.0	354.37	50.00	1	9	9	14	202	10.61	0.006791
A0A1D8PTW6	Hydroxymethylglutaryl-CoA synthase	49.7	352.68	19.73	6	6	6	7	451	5.97	0.001521
O13426	Serine hydroxymethyltransferase, cytosolic	52.0	352.58	29.36	7	13	13	14	470	7.20	0.002919
Q5AAW3	ATP-dependent RNA helicase DHH1	62.1	346.46	16.03	8	7	7	10	549	8.75	0.001785
Q5A762	Multiple drug resistance-associated protein-like transporter 1	180.6	338.87	8.90	1	11	11	11	1606	6.32	0.000671
P0CH96	Adenylosuccinate synthetase	47.9	334.93	26.87	14	12	12	13	428	7.17	0.002976
C4YG73	Vacuolar protein sorting/targeting protein 10	179.2	324.55	8.58	5	5	10	10	1586	5.03	0.000618
Q59PL9	Eukaryotic translation initiation factor 3 subunit A	106.0	323.18	15.16	5	11	11	11	930	8.31	0.001159
O93852	D-arabinono-1,4-lactone oxidase	63.4	317.04	23.88	1	10	10	11	557	6.61	0.001935
Q5AGZ9	RuvB-like helicase 2	54.5	315.00	18.07	12	6	6	7	498	5.15	0.001377
Q5AI86	Eukaryotic translation initiation factor 3 subunit I	38.2	311.63	24.00	6	7	7	7	350	5.47	0.001960
Q5APT8	ATP-dependent RNA helicase DBP3	63.1	307.43	17.73	13	10	10	10	564	9.45	0.001737
P47834	60S ribosomal protein L36	11.1	300.85	30.30	2	4	4	7	99	11.40	0.006929
Q5ACZ2	Mannan endo-1,6-alpha-mannosidase DFG5	50.0	300.43	12.42	1	4	4	4	451	4.65	0.000869
O94008	60S ribosomal protein L32	14.9	299.21	41.98	1	7	7	13	131	10.54	0.009724
Q59MN0	Vacuolar protein 8	63.4	298.44	11.97	5	5	5	6	585	5.07	0.001005
O94017	40S ribosomal protein S16	15.7	297.77	45.07	7	6	6	11	142	10.29	0.007591
Q5A0W7	RuvB-like helicase 1	50.0	295.66	22.93	4	7	7	7	458	6.01	0.001498
Q59XX2	Cell surface mannoprotein MP65	39.2	294.83	17.99	1	4	4	5	378	5.45	0.001296
Q5ALX8	Adenine phosphoribosyltransferase	20.9	293.58	37.23	1	5	5	7	188	5.31	0.003649
O43101	Centromere/microtubule-binding protein CBF5	54.3	291.55	26.72	2	9	9	10	479	9.20	0.002046

A0A1D8PH78	Farnesyl pyrophosphate synthase	40.7	289.35	20.51	2	6	6	7	351	4.98	0.001954
Q9P4E9	GTP-binding nuclear protein GSP1/Ran	24.3	281.78	57.94	7	2	9	13	214	7.02	0.005953
Q59L72	GPI-anchored protein 52	41.4	279.62	17.19	1	5	5	6	384	4.91	0.001531
P31353	Phosphomannomutase	29.0	279.35	33.33	3	8	8	9	252	5.69	0.003500
Q5AFE4	Regulator of cytoskeleton and endocytosis RVS161	30.1	278.24	32.58	1	6	6	7	264	7.14	0.002598
Q5ADM9	Dolichyl-phosphate-mannose--protein mannosyltransferase 2	88.3	277.52	6.89	1	4	4	5	769	7.21	0.000637
P43070	Glucan 1,3-beta-glucosidase	33.5	277.50	14.29	2	4	4	7	308	4.78	0.002227
Q5AI15	Polyadenylate-binding protein, cytoplasmic and nuclear	70.4	277.08	17.49	8	9	9	10	629	5.29	0.001558
P46596	Opaque-phase-specific protein OP4	41.3	273.67	9.70	1	2	2	3	402	5.26	0.000731
Q5AK59	ATP-dependent RNA helicase HAS1	63.0	272.25	17.35	29	7	7	7	565	8.91	0.001214
P46585	Ribose-phosphate pyrophosphokinase 1	35.3	272.21	15.89	1	4	4	6	321	6.79	0.001832
P82612	Phosphoglycerate mutase	27.4	264.01	29.44	1	5	5	7	248	6.16	0.002766
P83780	Glucose-6-phosphate isomerase	61.1	262.56	16.73	4	7	7	7	550	6.40	0.001247
Q59S06	Nucleolar protein 58	57.1	262.17	17.44	9	6	6	7	516	8.31	0.001329
P0CY31	Ras-related protein SEC4	23.1	261.54	31.90	1	5	5	7	210	5.47	0.003266
Q5ACU6	ATP-dependent rRNA helicase RRP3	59.8	261.10	20.60	8	8	8	9	534	9.73	0.001652
Q5A302	Endoplasmic reticulum vesicle protein 25	24.5	258.97	32.56	1	6	6	8	215	7.12	0.003646
Q9P8P7	Ribosomal RNA small subunit methyltransferase NEP1	29.5	257.99	35.58	2	6	6	7	267	8.43	0.002569
Q5A1D5	FACT complex subunit SPT16	121.3	253.65	10.09	1	9	9	9	1060	5.16	0.000832
O94150	37S ribosomal protein S9, mitochondrial	38.6	250.80	17.26	1	5	5	5	336	10.26	0.001458
P0CY33	Cell division control protein 42 homolog	21.2	250.23	14.66	1	2	2	5	191	6.54	0.002565
Q59R28	Alpha-1,2-mannosyltransferase MNN26	87.2	250.03	9.79	1	6	6	6	756	6.52	0.000778
Q5A5S7	Autophagy-related protein 27	28.3	239.78	26.59	1	5	5	5	252	5.53	0.001944
Q5A1L6	Major glycerophosphoinositol permease GIT3	59.4	232.47	10.09	1	3	3	5	535	8.06	0.000916
Q59L13	Eukaryotic translation initiation factor 6	26.4	230.98	22.45	7	4	4	5	245	4.58	0.002000
Q5ABV6	SWI5-dependent HO expression protein 3	59.2	230.81	10.02	4	4	4	5	519	9.20	0.000944
Q5AP66	Phosphatidylinositol transfer protein SFH5	36.6	230.73	22.19	1	5	5	5	320	5.33	0.001531
Q5ANE3	Non-classical export protein 102	18.1	229.67	20.59	1	2	2	4	170	9.01	0.002306
Q9UW25	Oxysterol-binding protein-like protein OBPa	49.5	229.55	19.17	4	7	7	8	433	6.13	0.001810
P29717	Glucan 1,3-beta-glucosidase	50.0	228.85	13.47	3	4	4	6	438	5.64	0.001342
P10977	Vacuolar aspartic protease	45.4	221.92	13.13	1	3	3	4	419	4.83	0.000935
Q9Y7F0	Peroxiredoxin TSA1	21.8	220.33	44.39	4	6	6	6	196	5.06	0.003000

O13432	Phenylalanine--tRNA ligase beta subunit	66.8	219.59	14.86	2	8	8	10	592	5.35	0.001655
Q59Q46	Inosine-5'-monophosphate dehydrogenase	56.2	211.92	5.76	1	2	2	3	521	6.55	0.000564
O74189	Dolichyl-phosphate-mannose--protein mannosyltransferase 1	99.9	211.75	9.92	1	6	6	6	877	6.95	0.000670
Q59ZH9	ATP-dependent RNA helicase MAK5	88.0	210.10	12.02	9	8	8	9	782	6.04	0.001128
Q59Y31	Yeast-form wall Protein 1	54.2	208.21	4.50	1	2	2	7	533	4.81	0.001287
P46592	Glycolipid 2-alpha-mannosyltransferase 2	54.5	205.43	16.49	1	5	7	7	461	6.67	0.001488
P83783	Adenosylhomocysteinase	49.0	200.91	17.78	2	6	6	7	450	5.59	0.001524
Q9B8D8	Cytochrome c oxidase subunit 2	29.8	199.21	17.94	12	3	3	4	262	4.68	0.001496
A0A1D8PD39	3-hydroxy-3-methylglutaryl-coenzyme A reductase 1	116.4	199.04	4.85	3	4	4	4	1073	7.33	0.000365
P78590	Elongation factor 1-beta	23.5	198.05	33.80	1	4	4	4	213	4.40	0.001840
P87220	V-type proton ATPase subunit D	30.0	198.00	7.49	1	1	1	2	267	5.85	0.000734
P30418	Glycylpeptide N-tetradecanoyltransferase	51.8	197.62	10.42	1	3	3	3	451	6.48	0.000652
O42617	Poly(A) polymerase PAPalpha	63.2	195.00	9.86	3	4	4	4	558	8.07	0.000702
O74933	UDP-N-acetylglucosamine pyrophosphorylase	54.6	192.24	14.20	1	5	5	5	486	6.28	0.001008
Q5ANB2	ATP-dependent RNA helicase DBP10	103.5	190.58	8.70	4	6	6	6	908	9.07	0.000648
P43060	Phosphoribosylaminoimidazole-succinocarboxamide synthase	32.9	188.89	16.84	3	4	4	5	291	5.50	0.001684
Q5AL27	Palmitoyltransferase AKR1	91.7	188.63	4.31	1	2	2	3	813	6.24	0.000362
Q5ANP2	Nascent polypeptide-associated complex subunit alpha	19.5	188.08	23.03	3	3	3	3	178	4.82	0.001652
Q59YF0	Protein transport protein SSO2	34.3	186.45	17.63	1	5	5	6	295	5.36	0.001993
P47831	60S ribosomal protein L28 (Fragment)	6.6	185.08	46.77	1	3	3	7	62	9.09	0.011063
P34725	Phospho-2-dehydro-3-deoxyheptonate aldolase, phenylalanine-inhibited	40.7	184.01	24.46	3	6	7	7	368	7.11	0.001864
Q59WB3	S-adenosylmethionine permease GAP4	66.4	182.97	9.06	1	5	5	5	607	7.46	0.000807
G1UB11	C-22 sterol desaturase ERG5	59.6	180.76	18.96	1	7	7	7	517	6.64	0.001327
Q5ADT9	37S ribosomal protein S10, mitochondrial	27.4	180.72	18.80	1	3	3	3	234	9.48	0.001256
P34948	Mannose-6-phosphate isomerase	48.8	172.35	16.10	1	6	6	6	441	5.33	0.001333
P22274	ADP-ribosylation factor	20.2	172.32	20.11	1	1	2	3	179	5.40	0.001642
Q874I4	Dihydroorotate dehydrogenase (quinone), mitochondrial	48.4	170.39	17.57	3	6	6	8	444	9.20	0.001766
Q59MA9	Clustered mitochondria protein homolog	155.7	169.56	5.72	2	7	7	7	1363	5.39	0.000503
Q5A455	Protein transport protein SEC23	85.6	167.84	5.91	5	4	4	4	762	5.71	0.000514
Q5AFA2	Extracellular glycosidase CRH11	46.7	166.79	13.69	1	5	5	6	453	4.96	0.001298
P43063	Cyclin-dependent kinase 1	36.6	166.54	21.14	16	6	6	6	317	6.61	0.001855
Q5A4Q1	Adenylate kinase	27.6	166.31	27.71	16	6	6	7	249	8.10	0.002755

P22011	Peptidyl-prolyl cis-trans isomerase	17.6	166.02	27.16	2	3	4	5	162	7.97	0.003024
P79023	Phospho-2-dehydro-3-deoxyheptonate aldolase, tyrosine-inhibited	40.3	165.91	16.22	3	4	5	5	370	6.65	0.001324
Q5AC48	Actin-related protein 4	52.6	165.17	12.18	1	4	4	5	468	5.57	0.001047
Q59MV9	Flavohemoprotein	45.8	164.38	19.85	1	5	5	6	398	5.83	0.001477
Q5AQ76	Protein transport protein SEC24	102.0	160.66	7.44	3	6	6	6	928	5.40	0.000634
Q5A4X0	E3 ubiquitin-protein ligase BRE1	78.5	159.67	3.67	1	2	2	2	681	6.14	0.000288
Q5AI21	Translocation protein SEC62	33.3	157.93	18.09	1	4	4	4	293	9.51	0.001338
C4YH95	tRNA (guanine(37)-N1)-methyltransferase	51.8	155.36	11.36	2	4	4	5	449	8.02	0.001091
Q59VP7	Ribosome biogenesis protein ERB1	97.3	154.80	8.01	11	4	6	6	849	4.74	0.000693
P46250	SEC14 cytosolic factor	34.7	154.76	23.59	5	6	6	7	301	6.40	0.002279
Q00314	Vanadate resistance protein	50.7	154.49	7.24	2	2	2	2	442	5.41	0.000443
Q59TU0	Nascent polypeptide-associated complex subunit beta	17.0	154.48	31.21	5	4	4	4	157	5.71	0.002497
P30572	Chitin synthase 2	115.5	153.59	4.56	1	3	3	4	1009	5.73	0.000388
Q8J0Q0	Mannosyl-oligosaccharide 1,2-alpha-mannosidase	64.6	153.13	15.58	1	7	7	8	565	5.11	0.001387
Q5ACK7	ATP-dependent RNA helicase DRS1	69.3	153.09	6.53	1	3	3	3	613	6.19	0.000480
Q5A1B0	Sterol-4-alpha-carboxylate 3-dehydrogenase ERG26, decarboxylating	39.2	151.67	11.71	1	3	3	4	350	6.73	0.001120
P40954	Chitinase 3	60.0	150.66	4.23	1	1	1	2	567	4.91	0.000346
P87023	Beta-glucan synthesis-associated protein KRE6	82.4	150.26	7.30	1	4	4	4	740	4.78	0.000530
O42779	Candidapepsin-9	58.6	149.64	8.09	2	4	4	4	544	5.25	0.000721
Q5AFT3	Protein CFT1	161.8	148.58	3.03	1	2	2	3	1420	5.21	0.000207
Q59P03	NADH-cytochrome b5 reductase 1	32.5	146.89	20.41	2	5	5	7	294	8.10	0.002333
Q59LF3	Regulator of cytoskeleton and endocytosis RVS167	49.2	146.10	17.73	1	5	5	6	440	6.02	0.001336
Q59NX9	Diphthine methyl ester synthase 1	33.9	145.88	15.38	1	4	4	5	299	4.87	0.001639
Q59ZV5	Eukaryotic translation initiation factor 3 subunit G	30.7	142.60	19.00	2	3	3	3	279	9.06	0.001054
Q59NP1	Copper transport protein CTR1	27.8	141.73	12.35	1	3	3	4	251	6.93	0.001562
P42800	Inositol-3-phosphate synthase	57.7	140.42	15.00	1	5	5	5	520	5.54	0.000942
Q9P975	Eukaryotic translation initiation factor 4E	24.2	139.69	20.57	1	4	4	5	209	5.26	0.002344
P87078	DNA topoisomerase 2	165.3	137.42	2.81	1	3	3	3	1461	6.40	0.000201
P43065	Saccharopine dehydrogenase [NAD(+), L-lysine-forming]	42.4	136.14	13.61	1	4	4	5	382	5.44	0.001283
Q5AEF2	Protein transport protein SEC13	33.0	132.94	15.44	1	4	4	4	298	5.62	0.001315
Q5AAR0	Transcription factor IWS1	45.8	132.65	15.50	2	5	5	5	400	8.12	0.001225
Q59KF3	AdoMet-dependent rRNA methyltransferase SPB1	96.8	132.39	2.84	1	2	2	2	845	5.76	0.000232

Q9P8E3	Protein transport protein SEC61 subunit alpha	52.5	130.69	10.44	2	4	4	6	479	8.95	0.001227
Q5A7M3	Kynurenine 3-monooxygenase	51.5	129.53	6.80	1	2	2	2	456	7.50	0.000430
O14427	Serine/threonine-protein kinase CLA4	106.8	128.75	4.63	2	4	4	4	971	9.35	0.000404
Q5ACI8	Peptidyl-prolyl cis-trans isomerase D	40.7	128.44	11.92	2	3	4	4	369	6.39	0.001062
Q59VX8	Septation protein 7	75.7	125.53	4.78	1	3	3	4	670	6.02	0.000585
P31225	Corticosteroid-binding protein	55.5	125.34	10.84	1	4	4	4	489	5.29	0.000802
P52498	Ras-related protein RSR1	27.6	125.34	18.95	2	4	4	4	248	5.21	0.001580
Q9P844	40S ribosomal protein S21	9.6	122.32	28.74	2	2	2	2	87	8.15	0.002253
A0A1D8PEL1	Mevalonate kinase	47.0	121.75	8.82	1	3	3	3	431	5.68	0.000682
P23286	Calmodulin	16.5	118.21	14.77	1	1	1	1	149	4.41	0.000658
Q5AJC0	Extracellular glycosidase UTR2	51.7	116.93	6.81	1	3	3	3	470	4.73	0.000625
Q5A061	Mitochondrial escape protein 2	99.3	116.67	3.11	1	2	2	2	867	7.84	0.000226
Q92210	Phosphoribosylaminoimidazole carboxylase	62.4	115.31	8.45	1	4	4	4	568	6.49	0.000690
Q9HGY5	Negative regulator of the PHO system	37.3	114.74	13.80	2	4	4	4	326	6.86	0.001202
Q5AP65	Protein FMP52, mitochondrial	24.3	113.18	17.03	1	4	4	4	229	8.98	0.001712
C4YLH0	MICOS complex subunit MIC60	62.6	111.85	5.49	2	3	3	3	565	6.44	0.000520
Q9HEW1	cAMP-dependent protein kinase regulatory subunit	50.3	110.58	6.10	1	2	2	2	459	5.48	0.000427
Q59WG0	Adenosine 5'-monophosphoramidase HNT1	17.0	110.57	23.68	1	2	2	2	152	6.86	0.001289
Q59X38	Pescadillo homolog	67.8	109.72	8.35	4	4	4	4	587	6.19	0.000668
Q5A4P9	ATP-dependent RNA helicase DBP9	65.1	109.47	5.40	2	3	3	3	574	9.06	0.000512
P0CU36	Ribosome biogenesis protein C3_06160C_A	29.6	109.09	9.58	7	2	2	3	261	10.18	0.001126
Q5AM84	U1 small nuclear ribonucleoprotein component SNU71	70.5	106.75	3.42	1	1	1	1	614	5.20	0.000160
Q5AF95	ATP-dependent RNA helicase DBP4	86.7	106.57	6.54	1	4	4	4	765	8.15	0.000512
Q59KG2	Respiratory growth induced protein 1	23.6	105.75	16.42	4	3	3	3	201	5.82	0.001463
C4YJI1	Altered inheritance of mitochondria protein 36, mitochondrial	34.7	103.61	11.99	3	3	3	3	292	8.34	0.001007
G1UB61	Septin CDC11	46.7	103.02	8.71	1	2	2	2	402	5.07	0.000488
A0A1D8PNZ7	Glycerophosphocholine phosphodiesterase GDE1	130.8	102.56	3.27	1	3	3	3	1162	6.30	0.000253
Q59WF4	Alpha-1,2-mannosyltransferase MNN2	69.1	100.79	8.71	1	4	4	4	597	6.27	0.000657
A0A1D8PLI2	Isopentenyl-diphosphate delta-isomerase	32.3	100.75	11.97	1	2	2	2	284	4.97	0.000690
A0A1D8PI71	Squalene synthase ERG9	51.2	100.31	12.50	2	6	6	7	448	6.84	0.001531
Q59W33	Glycerol-3-phosphate dehydrogenase [NAD(+)] 2	40.8	98.42	11.86	1	4	4	4	371	5.31	0.001056
C4YKP5	Increased recombination centers protein 22-1	25.9	95.96	22.18	5	3	3	3	239	5.68	0.001230

Q5ACM9	Eukaryotic translation initiation factor 3 subunit J	32.0	95.29	12.28	1	3	3	3	285	4.98	0.001031
Q5A744	Protein SDS23	67.5	95.04	7.17	1	3	3	3	628	8.44	0.000468
Q9HFQ6	60S acidic ribosomal protein P1-B	10.7	94.66	14.81	1	1	1	1	108	3.93	0.000907
C4YS65	Protein SEY1	90.2	93.95	4.30	5	2	2	2	790	5.15	0.000248
Q59WH0	Transcriptional adapter 2	51.1	93.00	5.39	1	2	2	2	445	7.46	0.000440
Q5AG77	Amino-acid permease GAP1	63.9	92.28	2.75	1	1	1	1	582	9.17	0.000168
O94072	V-type proton ATPase subunit E	25.4	90.46	11.95	2	2	2	2	226	5.40	0.000867
P53705	Bud site selection protein BUD4	192.8	88.11	1.11	1	1	1	1	1709	6.04	0.000057
Q5AEM8	Presequence translocated-associated motor subunit PAM17, mitochondrial	21.0	86.72	20.00	1	3	3	3	185	10.14	0.001589
P43094	Candidapepsin-5	45.6	84.77	5.98	1	2	2	2	418	6.38	0.000469
Q59W44	Mitochondrial import inner membrane translocase subunit TIM50	54.2	84.02	4.26	1	2	2	2	469	6.65	0.000418
Q59KJ7	Alpha-1,2-mannosyltransferase MNN21	76.9	83.08	2.12	1	1	1	1	660	7.11	0.000148
P87185	Cysteine desulfurase, mitochondrial	53.5	82.53	6.76	2	2	2	2	488	8.18	0.000402
Q5AH25	Exocyst complex protein EXO70	76.1	81.95	3.00	1	1	1	1	667	8.73	0.000147
Q5A360	Nucleotide exchange factor SIL1	47.0	81.82	4.59	1	1	1	1	414	5.07	0.000237
Q5A9Z6	ATP-dependent RNA helicase FAL1	45.5	80.74	3.26	1	1	1	1	399	8.02	0.000246
O74712	Histidine biosynthesis trifunctional protein	91.8	80.23	3.94	1	3	3	3	838	5.38	0.000351
Q9P4V2	Phosphoacetylglucosamine mutase	60.4	79.82	5.88	1	2	2	2	544	5.39	0.000360
Q9HF78	Glutamate--cysteine ligase	79.1	78.14	3.33	1	2	2	2	690	5.34	0.000284
Q59V93	Very-long-chain 3-oxoacyl-CoA reductase	38.3	77.67	8.31	1	3	3	3	349	9.57	0.000842
P46588	DNA polymerase delta catalytic subunit	118.8	77.45	2.60	1	2	2	2	1038	8.21	0.000189
Q5ABP8	Protein ROT1	29.9	76.50	9.23	1	2	2	2	260	7.80	0.000754
Q59PD6	Spindle assembly checkpoint component MAD1	82.2	76.21	2.30	1	1	1	1	696	6.93	0.000141
Q59S72	GDP-Man:Man(3)GlcNAc(2)-PP-Dol alpha-1,2-mannosyltransferase	70.9	75.67	4.43	1	2	2	2	609	8.53	0.000322
O74226	Cell wall synthesis protein KRE9	29.1	74.36	10.33	1	2	2	2	271	8.18	0.000723
Q96WL3	Protein URE2	39.1	74.34	8.43	1	2	2	2	344	5.94	0.000570
Q5AED9	Branchpoint-bridging protein	50.0	74.34	3.30	1	1	1	1	455	9.42	0.000215
Q5ALL8	FACT complex subunit POB3	60.9	73.86	5.20	1	2	2	2	538	4.83	0.000364
Q59PR3	ATP-dependent RNA helicase DBP8	48.8	73.58	6.59	13	2	3	3	440	8.91	0.000668
Q9UWF6	Lysophospholipase 1	66.4	72.52	2.15	1	1	1	1	605	4.87	0.000162
Q9Y7C4	ATP-dependent RNA helicase CHR1	65.4	72.38	8.65	5	4	4	4	578	9.23	0.000678
O13427	Low-specificity L-threonine aldolase	41.8	72.08	4.01	1	1	1	1	374	6.42	0.000262

Q5A2J7	Calcium channel YVC1	77.3	71.24	1.93	1	1	1	1	675	5.08	0.000145
P43068	Mitogen-activated protein kinase MKC1	58.2	70.88	3.79	62	1	2	2	501	5.02	0.000391
O13401	Superoxide dismutase [Mn], mitochondrial	26.2	70.67	7.69	1	1	1	1	234	8.73	0.000419
P53716	Uncharacterized protein CAWG_04269, mitochondrial	25.0	70.44	10.76	1	2	2	2	223	8.63	0.000879
Q5AGM0	Protein HIR2	114.4	69.92	1.18	1	1	1	1	1017	8.12	0.000096
Q00312	Transcription factor RBF1	59.4	69.84	2.85	2	1	1	1	527	8.66	0.000186
Q5ANL6	13 kDa ribonucleoprotein-associated protein	13.6	69.77	26.98	4	2	2	2	126	7.97	0.001555
Q00313	DNA topoisomerase 1	90.4	69.76	2.57	1	2	2	2	778	9.03	0.000252
C4YF50	NAD(P)H-hydrate epimerase	28.7	68.58	11.24	2	2	2	3	258	6.70	0.001139
A0A1D8PL26	2-(3-amino-3-carboxypropyl)histidine synthase subunit 2-2	59.1	67.65	2.65	2	1	1	1	529	5.73	0.000185
Q5AL52	Formin BNI1	196.7	67.33	0.81	1	1	1	1	1732	6.15	0.000057
O13359	Kexin	105.1	67.07	2.67	1	2	2	2	938	5.03	0.000209
Q5A216	Probable kinetochore protein NDC80	92.4	66.68	1.78	1	1	1	1	788	5.45	0.000124
Q59RP7	54S ribosomal protein L4, mitochondrial	36.3	66.63	8.74	1	3	3	3	309	5.96	0.000951
Q5AD56	General negative regulator of transcription subunit 3	85.7	65.84	1.99	1	1	1	1	752	5.57	0.000130
Q5AH60	tRNA (guanine-N(7)-)-methyltransferase non-catalytic subunit TRM82	49.1	65.82	4.69	1	1	1	1	426	5.26	0.000230
Q5A888	3-keto-steroid reductase ERG27	39.0	65.27	5.78	2	2	2	2	346	7.91	0.000566
Q5APQ8	Putative alpha-1,3-mannosyltransferase MNN12	97.5	65.22	1.57	1	1	1	1	828	8.16	0.000118
Q5AD78	Mannan endo-1,6-alpha-mannosidase DCW1	50.3	65.09	6.19	1	2	2	2	452	4.73	0.000434
Q5AJS6	Multiple RNA-binding domain-containing protein 1	94.9	64.64	1.55	1	1	1	1	841	5.62	0.000117
Q5AP53	Serine/threonine-protein kinase CBK1	84.4	64.23	3.96	1	3	3	3	732	6.40	0.000402
Q3MNT0	Transcription elongation factor SPT6	162.0	63.84	2.14	1	2	2	2	1401	5.24	0.000140
O93875	Delta(7)-sterol 5(6)-desaturase	45.4	63.76	7.51	3	3	3	3	386	6.80	0.000762
Q5A4E3	Initiation-specific alpha-1,6-mannosyltransferase	44.8	63.39	4.68	1	1	1	1	385	8.44	0.000255
Q5AQ57	37S ribosomal protein S25, mitochondrial	34.2	63.08	8.14	1	2	2	2	295	8.03	0.000664
Q9B8C9	NADH-ubiquinone oxidoreductase chain 5	61.5	62.93	3.44	1	2	2	2	552	6.74	0.000355
Q5A477	GDP-mannose transporter	41.2	62.85	2.70	1	1	1	4	371	9.36	0.001056
Q59KI4	Chromatin-remodeling ATPase INO80	158.7	62.52	2.67	1	3	3	3	1387	8.09	0.000212
P53697	Mannan polymerase complex subunit MNN9	42.8	62.12	15.76	25	3	4	4	368	9.14	0.001065
P0CB63	Golgi to ER traffic protein 2	33.6	61.83	4.03	1	1	1	1	298	5.52	0.000329
O74161	Chitin biosynthesis protein CHS5	62.1	60.76	1.96	1	1	1	1	562	4.59	0.000174
P53698	Cytochrome c	12.2	60.17	25.45	22	3	3	4	110	9.66	0.003563

Q5AKU5	Secreted beta-glucosidase SIM1	39.4	60.12	5.38	1	1	1	1	372	4.87	0.000263
Q59KM8	Cell cycle protein kinase DBF2	82.3	59.83	1.69	1	1	1	2	710	9.13	0.000276
O93831	Rab proteins geranylgeranyltransferase component A	72.2	59.02	2.81	1	2	2	2	640	4.81	0.000306
Q5A246	DNA-directed RNA polymerase III subunit RPC3	68.3	58.41	1.85	1	1	1	1	595	8.85	0.000165
Q5ABG1	Histone-lysine N-methyltransferase, H3 lysine-4 specific	119.1	57.86	1.44	1	1	1	1	1040	7.75	0.000094
Q59S59	ATP-dependent RNA helicase MRH4, mitochondrial	62.5	55.92	2.88	1	1	1	1	555	9.70	0.000177
Q5AAI8	Nucleosome assembly protein 1	49.5	54.67	6.90	1	2	2	2	435	4.31	0.000451
Q9UVL1	Non-histone chromosomal protein 6	10.5	54.48	28.26	2	2	2	2	92	9.70	0.002130
Q5AHB1	Actin cytoskeleton-regulatory complex protein PAN1	152.3	54.08	1.22	1	1	1	1	1396	6.23	0.000070
Q9HFQ7	60S acidic ribosomal protein P1-A	11.0	53.62	14.15	1	1	1	1	106	4.02	0.000924
Q5A3P6	Serine/threonine-protein kinase PKH2	106.5	53.17	1.80	1	1	1	1	947	8.28	0.000103
P87219	Sorbose reductase SOU1	30.0	53.08	5.34	1	1	1	1	281	5.30	0.000349
A0A1D8PLH0	Phosphomevalonate kinase	48.2	52.38	3.70	1	1	1	1	432	5.44	0.000227
Q5A8X7	Protein FYV4, mitochondrial	19.8	52.15	14.12	1	2	2	2	170	9.99	0.001153
Q5AHJ5	Transcription factor TBF1	100.8	51.55	1.13	1	1	1	1	886	4.67	0.000111
P53699	Cell division control protein 4	76.0	51.48	2.05	1	1	1	1	684	5.95	0.000143
Q5AI97	Mitochondrial genome maintenance protein MGM101	30.7	50.56	5.45	2	1	1	1	275	9.10	0.000356
P52499	Protein RCC1	51.3	49.58	2.80	1	1	1	1	464	5.43	0.000211
Q5A310	ISWI chromatin-remodeling complex ATPase ISW2	123.0	49.42	1.14	1	1	1	1	1056	6.73	0.000093
Q5A7S5	Pre-mRNA-splicing factor RSE1	136.3	49.40	1.72	1	1	1	1	1219	5.83	0.000080
O14415	Signal recognition particle SEC65 subunit	31.1	49.14	4.00	1	1	1	1	275	7.53	0.000356
Q9HFQ5	60S acidic ribosomal protein P2-A	10.9	48.74	11.11	1	1	1	1	108	3.93	0.000907
Q5AD51	Ergosterol biosynthetic protein 28	16.0	48.34	7.97	1	1	1	1	138	9.31	0.000710
P13649	Orotidine 5'-phosphate decarboxylase	29.9	47.77	2.96	3	1	1	1	270	5.76	0.000363
Q5AA50	Nuclear protein localization protein 4	67.3	47.25	2.34	1	1	1	1	598	6.29	0.000164
P87024	Beta-glucan synthesis-associated protein SKN1	83.7	46.98	2.44	1	1	1	1	737	5.15	0.000133
P56091	Galactokinase	57.0	46.92	1.75	1	1	1	1	515	5.54	0.000190
Q59MF9	Conserved oligomeric Golgi complex subunit 6	88.4	46.16	1.30	1	1	1	1	771	5.03	0.000127
Q5A2V2	Protein RMD9, mitochondrial	70.2	45.83	2.45	1	1	1	1	613	7.17	0.000160
O93803	mRNA-capping enzyme subunit beta	58.8	44.66	3.08	1	1	1	1	520	7.87	0.000188
Q5A2A2	Mitochondrial homologous recombination protein 1	28.3	44.30	4.60	1	1	1	1	239	8.50	0.000410
P39827	Cell division control protein 10	40.7	43.87	5.32	1	1	1	1	357	7.11	0.000274

P0C8K9	Cytochrome c oxidase subunit 1	58.7	43.62	8.10	1	2	2	2	531	6.37	0.000369
Q59QC6	Assembly factor CBP4	17.1	43.36	7.64	1	1	1	1	144	8.09	0.000680
Q92207	Mitogen-activated protein kinase HOG1	42.9	42.32	8.75	63	3	4	4	377	5.19	0.001040
C4YTG0	Pheromone-processing carboxypeptidase KEX1	78.7	42.24	1.28	2	1	1	1	702	5.12	0.000140
Q5A761	CCR4-Not complex 3'-5'-exoribonuclease subunit Ccr4	90.3	41.89	2.54	2	2	2	3	787	7.20	0.000374
Q5A1Q5	Probable kinetochore protein NUF2	56.6	41.51	1.86	1	1	1	1	485	5.55	0.000202
Q9HFQ4	60S acidic ribosomal protein P2-B	11.2	41.32	10.81	1	1	1	1	111	4.07	0.000883
Q9B8D1	NADH-ubiquinone oxidoreductase chain 3	14.7	40.95	7.75	1	1	1	1	129	5.06	0.000760
Q5AAJ7	SWR1-complex protein 4	74.0	40.52	1.42	1	1	1	1	635	8.19	0.000154
Q5AG40	Vacuolar protein sorting-associated protein 4	48.4	40.31	2.51	1	1	1	1	439	5.64	0.000223
O74270	Origin recognition complex subunit 1	91.2	40.18	1.74	1	1	1	1	805	6.25	0.000122
Q59N80	Inosine triphosphate pyrophosphatase	22.0	39.70	4.46	1	1	1	1	202	5.66	0.000485
Q5A3V6	3,4-dihydroxy-2-butanone 4-phosphate synthase	22.6	38.68	3.43	1	1	1	1	204	5.44	0.000480
O42816	Signal recognition particle 54 kDa protein homolog	60.7	38.67	3.42	1	2	2	2	556	9.13	0.000352
Q5A599	Histidine protein kinase NIK1	118.9	38.14	1.30	1	1	1	1	1081	5.80	0.000091
Q59Y46	Morphogenetic regulator of filamentous growth protein 1	83.4	36.94	1.06	1	1	1	1	758	10.17	0.000129
Q5AHZ7	Mediator of RNA polymerase II transcription subunit 17	65.6	36.45	2.12	1	1	1	1	565	5.45	0.000173
Q59VP2	Histone H2A.2	13.8	34.88	18.32	1	1	1	1	131	10.24	0.000748
Q5ACU3	Dolichyl-phosphate-mannose--protein mannosyltransferase 5	84.5	33.74	1.52	1	1	1	1	725	7.80	0.000135
P87163	Translation initiation factor eIF-2B subunit epsilon	81.9	33.63	1.64	1	1	1	1	732	4.65	0.000134
P78600	Proline--tRNA ligase, cytoplasmic	66.2	33.06	2.43	1	1	1	1	575	5.96	0.000170
Q5A3M6	GPI ethanolamine phosphate transferase 1	110.6	32.99	0.93	1	1	1	1	971	5.96	0.000101
A0A1D8PNP3	Amino-acid permease GAP6	61.8	32.70	1.58	1	1	1	1	568	8.72	0.000173
Q5ABD0	Vacuolar-sorting protein SNF7	25.9	31.41	5.31	1	1	1	1	226	4.88	0.000434
Q9UVX1	Lysophospholipase 3	81.4	30.91	1.06	1	1	1	1	754	4.84	0.000130
Q59U67	Transcription initiation factor TFIID subunit 4	39.6	30.88	2.75	1	1	1	1	363	9.13	0.000270
Q5A309	Histone-lysine N-methyltransferase, H3 lysine-79 specific	155.6	30.59	0.89	1	1	1	1	1343	6.38	0.000073
Q9P836	60S ribosomal protein L37 (Fragment)	9.9	30.36	7.95	1	1	1	1	88	11.63	0.001114
C4YS59	Vacuolar membrane protease	93.7	30.32	1.79	2	1	1	1	837	5.29	0.000117
Q92410	Alpha,alpha-trehalose-phosphate synthase [UDP-forming]	54.4	30.06	1.46	1	1	1	1	478	6.23	0.000205
A0A1D8PH52	Acetyl-CoA acetyltransferase	41.9	29.99	5.22	1	2	2	2	402	6.90	0.000488
P23316	Chitin synthase 1	88.2	29.57	2.45	1	1	1	1	776	8.63	0.000126

Q59LF2	Alpha-1,3/1,6-mannosyltransferase ALG2	50.4	29.37	2.26	1	1	1	1	443	8.31	0.000221
A0A1D8PCB9	C-8 sterol isomerase ERG2	24.5	27.42	8.76	1	2	2	2	217	5.48	0.000903
Q5ACM4	Pre-rRNA-processing protein PNO1	30.6	27.39	3.62	1	1	1	1	276	8.90	0.000355
P30574	Carboxypeptidase Y	61.0	27.17	1.48	1	1	1	1	542	5.47	0.000181
Q5AK62	Virulence protein SSD1	141.2	25.20	1.10	1	1	1	1	1274	7.36	0.000077
A0A1D8PN88	Amino-acid permease GAP3	65.5	24.74	1.34	1	1	1	1	599	8.43	0.000164
Q59U81	Helicase SWR1	187.6	24.44	0.55	1	1	1	1	1641	5.58	0.000060
A0A1D8PC43	Diphosphomevalonate decarboxylase	39.5	23.65	3.87	1	1	1	1	362	6.46	0.000271
A0A1D8PJ25	Delta(24(24(1)))-sterol reductase	54.8	23.45	2.13	1	1	1	1	469	7.06	0.000209
Q5AP90	Alpha-1,2-mannosyltransferase MNN23	69.6	23.37	1.32	1	1	1	1	606	5.40	0.000162
Q04782	Lanosterol synthase	83.7	23.25	2.06	1	1	1	1	728	5.92	0.000135
Q59XM1	Exocyst complex component EXO84	88.5	22.65	1.26	1	1	1	1	791	8.98	0.000124
Q59N29	ATP-dependent rRNA helicase SPB4	72.3	21.68	2.22	1	1	1	1	631	9.10	0.000155
C4YFX2	Transcriptional repressor TUP1	57.5	21.25	2.54	2	1	1	1	511	6.00	0.000192
Q5AAG1	Enhancer of polycomb-like protein 1	87.4	20.04	1.33	1	1	1	1	753	9.10	0.000130
Q5AAV3	Ribosome-releasing factor 2, mitochondrial	90.2	14.88	1.49	1	1	1	1	807	5.87	0.000121
**EV_{FLU}**											
Q92211	Glyceraldehyde-3-phosphate dehydrogenase	35.8	7441.01	84.18	34	23	26	214	335	7.12	0.072071
P28877	Plasma membrane ATPase 1	97.4	3768.33	36.20	8	18	26	93	895	4.96	0.011723
P83779	Pyruvate decarboxylase	62.4	3538.24	58.91	4	24	24	83	567	5.58	0.016515
P0CY35	Elongation factor 1-alpha 1	50.0	3165.29	50.22	20	6	20	152	458	9.03	0.037443
P25997	Elongation factor 3	116.9	3136.05	48.57	6	34	39	87	1050	5.73	0.009348
P41797	Heat shock protein SSA1	70.3	3084.40	54.27	11	10	28	89	656	5.17	0.015307
P46587	Heat shock protein SSA2	70.0	3024.55	64.50	9	15	32	90	645	5.06	0.015743
P87222	Heat shock protein SSB1	66.4	2630.24	54.00	9	23	23	63	613	5.38	0.011595
Q9URB4	Fructose-bisphosphate aldolase	39.2	1984.30	72.14	3	19	19	56	359	6.06	0.017599
P43067	Alcohol dehydrogenase 1	36.9	1893.35	62.00	4	17	19	67	350	6.44	0.021597
P83784	Heat shock protein SSC1, mitochondrial	69.7	1880.89	52.62	3	28	28	44	648	5.60	0.007661
P34731	Fatty acid synthase subunit beta	227.8	1718.05	24.35	5	39	39	45	2037	6.02	0.002492
C4YJQ8	Elongation factor 2	93.3	1658.77	41.92	13	32	32	50	842	6.47	0.006700
P43076	pH-responsive protein 1	59.4	1603.02	47.45	2	23	24	35	548	5.07	0.007206
P83781	Mitochondrial outer membrane protein porin	29.7	1520.49	58.87	1	14	14	37	282	8.57	0.014803

P14235	Actin	41.7	1519.61	50.53	30	16	16	47	376	5.69	0.014103
Q96VB9	Heat shock protein homolog SSE1	78.5	1351.72	52.64	5	29	29	37	701	5.30	0.005955
O13434	Phosphoenolpyruvate carboxykinase [ATP]	60.8	1328.65	52.08	2	22	22	35	553	6.60	0.007141
P83776	Hexokinase-2	53.4	1313.27	56.82	4	21	21	39	484	5.55	0.009091
P43098	Fatty acid synthase subunit alpha	207.5	1288.73	20.05	9	31	31	32	1885	5.62	0.001915
O94049	Acetyl-coenzyme A synthetase 1	75.1	1213.54	46.22	6	26	28	39	675	6.32	0.006519
Q9Y872	Sulfate adenylyltransferase	58.8	1095.50	48.39	5	21	21	30	527	6.40	0.006422
P83782	Cytochrome b-c1 complex subunit 2, mitochondrial	39.5	1082.81	71.12	1	17	17	22	374	5.57	0.006637
O74676	ABC transporter CDR4	169.2	1079.63	20.94	7	22	24	25	1490	7.65	0.001893
Q59PT0	V-type proton ATPase subunit B	57.2	1040.31	46.48	5	2	19	24	512	5.03	0.005289
O74261	Heat shock protein 60, mitochondrial	60.1	996.49	46.29	6	19	19	25	566	5.30	0.004983
P83778	Malate dehydrogenase, cytoplasmic	36.0	960.94	61.13	1	15	15	26	337	5.62	0.008704
Q59KZ1	Aminopeptidase 2	104.3	942.39	31.60	2	24	24	24	924	5.36	0.002930
P30575	Enolase 1	47.2	884.67	49.77	11	15	15	21	440	5.81	0.005385
P46598	Heat shock protein 90 homolog	80.8	883.97	29.56	7	19	19	23	707	4.88	0.003670
P43071	Multidrug resistance protein CDR1	169.8	881.38	20.99	6	24	26	30	1501	6.98	0.002255
P53707	37 kDa cell surface protein	37.0	881.19	39.88	1	12	12	24	321	7.06	0.008435
P10613	Lanosterol 14-alpha demethylase	60.6	777.57	39.20	5	19	19	22	528	7.17	0.004701
Q96UX5	Heat shock protein 78, mitochondrial	91.6	762.97	29.31	4	17	17	19	812	6.80	0.002640
Q5A8K2	Alanine--tRNA ligase	108.2	747.38	28.28	3	23	23	26	969	6.07	0.003027
Q5AML1	Eukaryotic translation initiation factor 3 subunit C	99.8	722.40	27.57	7	20	20	21	874	5.15	0.002711
Q5AJB1	V-type proton ATPase catalytic subunit A	67.6	720.58	25.61	6	12	12	13	617	5.27	0.002377
Q5AEN1	Cytochrome c peroxidase, mitochondrial	40.7	685.03	43.44	1	12	12	18	366	6.34	0.005549
O94038	Alcohol dehydrogenase 2	36.8	679.91	52.59	7	14	16	20	348	6.68	0.006484
C4YG73	Vacuolar protein sorting/targeting protein 10	179.2	676.67	15.89	5	12	19	19	1586	5.03	0.001352
Q8NJN3	Acetyl-coenzyme A synthetase 2	73.8	672.37	30.25	12	15	17	22	671	6.13	0.003699
Q5AHH4	Small heat shock protein 21	21.5	670.41	61.90	1	10	10	22	189	5.35	0.013133
P82611	Aconitate hydratase, mitochondrial	84.2	663.26	30.12	2	16	16	19	777	6.39	0.002759
O94039	Transketolase 1	73.7	655.23	26.44	1	14	14	19	677	5.82	0.003166
Q00310	Glycolipid 2-alpha-mannosyltransferase 1	50.0	613.49	46.17	2	14	16	20	431	6.90	0.005235
Q9HGT6	Serine--tRNA ligase, cytoplasmic	53.0	612.29	41.77	4	16	16	17	462	5.73	0.004151
P46273	Phosphoglycerate kinase	45.2	606.77	49.40	21	18	18	21	417	6.48	0.005682

O74198	Sterol 24-C-methyltransferase	43.0	602.93	44.68	3	13	13	18	376	6.11	0.005401
Q5AJY5	1,3-beta-glucanosyltransferase PGA4	49.0	602.15	26.83	1	9	9	12	451	4.74	0.003002
Q5A762	Multiple drug resistance-associated protein-like transporter 1	180.6	598.43	12.52	2	16	17	17	1606	6.32	0.001194
P40910	40S ribosomal protein S1	29.0	597.95	52.34	53	13	13	19	256	10.04	0.008373
P82610	5-methyltetrahydropteroyltriglutamate--homocysteine methyltransferase	85.6	587.87	26.73	2	14	14	16	767	5.60	0.002354
C4YKT4	Ras-like protein 1	32.2	587.85	52.43	3	8	8	11	288	4.67	0.004309
P43057	Protein kinase C-like 1	125.2	586.34	17.96	1	15	15	15	1097	7.42	0.001543
P83774	Guanine nucleotide-binding protein subunit beta-like protein	34.5	581.37	52.37	1	11	11	15	317	6.54	0.005339
Q8TGH6	Guanosine-diphosphatase	65.9	577.14	29.05	1	13	13	15	599	5.94	0.002825
Q9P8Q7	Isocitrate lyase	61.4	564.52	32.18	17	14	14	17	550	7.11	0.003487
O42825	GTP-binding protein RHO1	22.0	560.45	56.57	7	10	10	13	198	5.73	0.007407
O42817	40S ribosomal protein S0	28.7	559.02	44.06	33	10	10	16	261	4.91	0.006916
P30573	Chitin synthase 3	136.1	546.04	15.83	9	15	15	16	1213	7.17	0.001488
P10875	Tubulin beta chain	49.9	541.21	34.08	13	10	10	13	449	4.74	0.003267
C4YLH0	MICOS complex subunit MIC60	62.6	541.13	27.08	4	14	14	15	565	6.44	0.002995
P47837	40S ribosomal protein S4	29.2	514.84	41.60	1	7	10	17	262	10.21	0.007320
Q59M70	NADH-cytochrome b5 reductase 2	33.4	514.59	44.52	2	13	13	17	301	8.48	0.006372
P34732	Vesicular-fusion protein SEC18	88.9	505.78	18.89	2	10	11	13	794	7.43	0.001847
Q5AJD0	ATP-dependent RNA helicase DBP5	60.2	493.24	34.81	12	14	14	14	540	5.76	0.002925
O93852	D-arabinono-1,4-lactone oxidase	63.4	485.45	30.52	1	13	13	17	557	6.61	0.003443
P47828	T-complex protein 1 subunit theta	58.9	483.30	28.15	2	12	12	14	540	7.97	0.002925
O93827	Mannose-1-phosphate guanyltransferase	40.0	478.74	52.76	15	16	16	17	362	6.30	0.005298
Q92206	Squalene monooxygenase	55.3	462.03	29.84	1	12	12	15	496	8.73	0.003412
P83777	Inorganic pyrophosphatase	32.1	455.02	55.21	12	11	11	14	288	5.26	0.005484
P87206	ATP-dependent RNA helicase eIF4A	44.6	448.52	37.03	40	13	13	14	397	5.36	0.003979
Q59S78	Small COPII coat GTPase SAR1	21.5	447.63	61.58	27	9	9	12	190	5.59	0.007126
Q9P843	60S ribosomal protein L27	15.5	443.18	47.79	1	7	7	13	136	10.18	0.010784
Q59MN0	Vacuolar protein 8	63.4	442.84	14.70	7	6	6	8	585	5.07	0.001543
Q5APD4	Sphingolipid C9-methyltransferase	58.7	440.38	23.20	2	12	12	12	513	6.92	0.002639
O42766	14-3-3 protein homolog	29.5	437.55	27.27	8	6	6	10	264	4.81	0.004274
Q5A4E2	ATP-dependent RNA helicase DED1	72.8	432.96	42.26	45	17	19	21	672	8.28	0.003526
P83773	Acetyl-CoA hydrolase	58.0	426.30	33.21	8	14	14	15	524	6.92	0.003230

O13318	pH-responsive protein 2	58.7	426.01	11.95	1	6	7	9	544	4.64	0.001867
O13287	6-phosphogluconate dehydrogenase, decarboxylating	56.9	423.09	20.31	4	10	10	13	517	6.57	0.002837
P43084	Probable NADPH dehydrogenase	46.0	418.05	31.20	2	10	10	11	407	6.39	0.003049
Q59R28	Alpha-1,2-mannosyltransferase MNN26	87.2	417.45	21.69	1	15	15	15	756	6.52	0.002239
A0A1D8PTW6	Hydroxymethylglutaryl-CoA synthase	49.7	411.71	22.84	6	7	7	8	451	5.97	0.002001
A0A1D8PCL1	High-affinity glucose transporter 1	60.6	410.52	19.45	1	9	9	10	545	7.62	0.002070
Q5AL27	Palmitoyltransferase AKR1	91.7	404.64	14.15	1	8	8	10	813	6.24	0.001388
Q59KI0	UTP--glucose-1-phosphate uridylyltransferase	55.5	403.85	19.20	1	7	7	9	500	6.73	0.002031
Q5ACZ2	Mannan endo-1,6-alpha-mannosidase DFG5	50.0	396.60	20.62	1	7	7	7	451	4.65	0.001751
P0CY31	Ras-related protein SEC4	23.1	396.15	49.05	1	7	7	9	210	5.47	0.004835
Q5ADM9	Dolichyl-phosphate-mannose--protein mannosyltransferase 2	88.3	395.90	14.30	3	11	11	12	769	7.21	0.001761
O74189	Dolichyl-phosphate-mannose--protein mannosyltransferase 1	99.9	390.91	12.43	1	8	8	12	877	6.95	0.001544
Q59YF0	Protein transport protein SSO2	34.3	390.82	23.39	1	6	6	10	295	5.36	0.003824
Q5ALX3	Transcription elongation factor SPT5	105.8	387.75	12.66	1	9	9	9	956	5.16	0.001062
Q9UW25	Oxysterol-binding protein-like protein OBPa	49.5	384.52	32.56	4	13	13	15	433	6.13	0.003908
Q59PL9	Eukaryotic translation initiation factor 3 subunit A	106.0	380.24	15.27	5	12	12	14	930	8.31	0.001698
O94201	ATP-dependent 6-phosphofructokinase subunit alpha	108.5	375.69	19.15	1	15	15	15	987	6.62	0.001715
Q874I4	Dihydroorotate dehydrogenase (quinone), mitochondrial	48.4	374.06	25.68	3	8	8	11	444	9.20	0.002795
Q5AIR7	Endo-1,3(4)-beta-glucanase 1	124.0	369.69	9.96	1	8	8	8	1145	5.40	0.000788
P46614	Pyruvate kinase	55.4	369.21	24.80	10	8	8	9	504	6.99	0.002015
P52495	Ubiquitin-activating enzyme E1 1	114.2	368.03	11.56	2	9	9	9	1021	5.01	0.000995
Q5AKA5	Cys-Gly metallodipeptidase DUG1	53.6	357.89	25.57	1	8	8	8	485	5.24	0.001861
P87066	Tubulin alpha chain	49.9	354.72	31.47	16	11	11	13	448	5.06	0.003274
O13354	Eukaryotic peptide chain release factor GTP-binding subunit	79.0	347.80	13.85	7	8	8	9	715	8.47	0.001420
Q59WB3	S-adenosylmethionine permease GAP4	66.4	343.64	12.36	1	8	8	9	607	7.46	0.001673
O13289	Peroxisomal catalase	54.8	343.03	23.09	2	9	9	11	485	6.65	0.002559
A0A1D8PN12	Glycerophosphodiester transporter GIT2	59.3	342.84	17.79	1	8	8	10	534	8.10	0.002113
O94200	ATP-dependent 6-phosphofructokinase subunit beta	104.0	340.94	12.05	2	10	10	11	946	6.35	0.001312
Q5A4M8	Protein SUR7	29.9	339.84	32.22	1	5	5	6	270	7.69	0.002507
Q96W54	40S ribosomal protein S22	14.8	334.06	52.31	9	6	6	9	130	9.88	0.007811
Q5A0W7	RuvB-like helicase 1	50.0	333.46	31.88	9	8	8	8	458	6.01	0.001971
O94083	Eukaryotic translation initiation factor 5A	17.1	331.87	52.53	6	7	7	11	158	5.05	0.007855

Q59PR9	Transcriptional regulator HMO1	24.8	328.49	33.18	1	5	5	8	223	9.19	0.004047
P53704	Glutamine--fructose-6-phosphate aminotransferase [isomerizing]	79.2	327.74	15.15	2	8	8	8	713	6.24	0.001266
Q96W53	40S ribosomal protein S14	14.4	325.05	40.30	4	5	5	7	134	10.61	0.005894
O13426	Serine hydroxymethyltransferase, cytosolic	52.0	322.20	19.15	7	9	9	10	470	7.20	0.002400
A0A1D8PD39	3-hydroxy-3-methylglutaryl-coenzyme A reductase 1	116.4	316.24	8.29	3	6	6	6	1073	7.33	0.000631
Q59LF3	Regulator of cytoskeleton and endocytosis RVS167	49.2	316.00	25.23	1	7	7	7	440	6.02	0.001795
O94017	40S ribosomal protein S16	15.7	313.87	50.00	7	7	7	10	142	10.29	0.007945
Q5AAW3	ATP-dependent RNA helicase DHH1	62.1	309.40	20.40	9	9	9	11	549	8.75	0.002261
Q5AGV4	Eukaryotic translation initiation factor 3 subunit B	84.2	302.80	18.94	5	12	12	13	739	6.39	0.001985
Q5A5S7	Autophagy-related protein 27	28.3	300.97	29.37	1	6	6	7	252	5.53	0.003134
Q5ALX8	Adenine phosphoribosyltransferase	20.9	299.53	42.02	1	6	6	8	188	5.31	0.004801
Q9UVJ4	60S ribosomal protein L10a	24.4	298.83	34.10	4	7	7	8	217	9.76	0.004159
P47834	60S ribosomal protein L36	11.1	298.73	39.39	8	6	6	8	99	11.40	0.009117
Q5AP66	Phosphatidylinositol transfer protein SFH5	36.6	290.23	27.50	1	6	6	6	320	5.33	0.002115
Q5A061	Mitochondrial escape protein 2	99.3	288.98	9.69	4	8	8	8	867	7.84	0.001041
Q9P4E9	GTP-binding nuclear protein GSP1/Ran	24.3	285.83	51.87	8	9	9	11	214	7.02	0.005799
P0CH96	Adenylosuccinate synthetase	47.9	283.78	26.17	20	11	11	13	428	7.17	0.003427
Q59LU0	ATP-dependent RNA helicase DBP2	61.2	281.80	18.51	32	8	10	12	562	8.88	0.002409
P43070	Glucan 1,3-beta-glucosidase	33.5	279.77	16.56	3	5	5	8	308	4.78	0.002930
Q5AME2	Pentafunctional AROM polypeptide	169.3	278.41	5.93	7	7	7	7	1551	6.58	0.000509
O59931	60S ribosomal protein L13	23.0	278.23	41.09	2	7	7	12	202	10.61	0.006702
Q59ZX6	U3 small nucleolar RNA-associated protein 10	204.2	272.84	4.51	4	7	7	7	1818	5.87	0.000434
P39826	Cell division control protein 3	47.8	266.20	15.14	3	5	5	6	416	6.64	0.001627
P46596	Opaque-phase-specific protein OP4	41.3	265.98	9.70	1	2	2	3	402	5.26	0.000842
P46592	Glycolipid 2-alpha-mannosyltransferase 2	54.5	265.93	20.17	1	7	9	9	461	6.67	0.002203
C4YS65	Protein SEY1	90.2	265.84	10.38	5	6	6	6	790	5.15	0.000857
Q5A302	Endoplasmic reticulum vesicle protein 25	24.5	264.05	20.00	1	5	5	8	215	7.12	0.004198
Q5A2J7	Calcium channel YVC1	77.3	263.45	8.74	1	5	5	8	675	5.08	0.001337
Q5AI15	Polyadenylate-binding protein, cytoplasmic and nuclear	70.4	259.17	15.58	9	9	9	10	629	5.29	0.001794
P31353	Phosphomannomutase	29.0	258.18	30.16	3	7	7	8	252	5.69	0.003582
Q5AI86	Eukaryotic translation initiation factor 3 subunit I	38.2	256.77	22.00	9	7	7	7	350	5.47	0.002256
P87220	V-type proton ATPase subunit D	30.0	256.64	23.60	1	4	4	5	267	5.85	0.002113

O94008	60S ribosomal protein L32	14.9	247.70	41.22	1	6	6	9	131	10.54	0.007751
O94150	37S ribosomal protein S9, mitochondrial	38.6	244.79	20.83	1	6	6	7	336	10.26	0.002350
O13425	Serine hydroxymethyltransferase, mitochondrial	54.5	242.18	17.65	3	7	7	7	493	8.97	0.001602
P79023	Phospho-2-dehydro-3-deoxyheptonate aldolase, tyrosine-inhibited	40.3	240.78	21.89	3	5	6	6	370	6.65	0.001830
Q5AFE4	Regulator of cytoskeleton and endocytosis RVS161	30.1	238.97	44.70	1	10	10	10	264	7.14	0.004274
Q9P940	Triosephosphate isomerase	26.6	236.29	39.11	3	8	8	9	248	6.01	0.004094
Q5APT8	ATP-dependent RNA helicase DBP3	63.1	236.24	19.15	14	9	9	9	564	9.45	0.001800
Q59MQ0	Myosin-5	146.9	235.24	6.84	17	8	8	9	1316	9.35	0.000772
P10977	Vacuolar aspartic protease	45.4	233.04	13.13	1	3	3	4	419	4.83	0.001077
Q8J0Q0	Mannosyl-oligosaccharide 1,2-alpha-mannosidase	64.6	229.14	15.40	1	7	7	7	565	5.11	0.001398
O43101	Centromere/microtubule-binding protein CBF5	54.3	229.06	25.05	1	7	7	7	479	9.20	0.001649
Q59L72	GPI-anchored protein 52	41.4	228.99	17.19	1	5	5	5	384	4.91	0.001469
Q00314	Vanadate resistance protein	50.7	227.83	13.80	2	4	4	4	442	5.41	0.001021
Q5AI37	Probable metalloprotease ARX1	62.2	225.63	15.60	1	7	7	7	564	6.92	0.001400
Q59XX2	Cell surface mannoprotein MP65	39.2	219.87	11.90	1	3	3	4	378	5.45	0.001194
P0CY33	Cell division control protein 42 homolog	21.2	219.46	19.90	3	3	3	6	191	6.54	0.003544
Q5AAU3	Protein transport protein SEC31	136.2	219.26	6.32	1	5	5	5	1265	6.77	0.000446
Q5AD78	Mannan endo-1,6-alpha-mannosidase DCW1	50.3	217.61	14.60	1	4	4	5	452	4.73	0.001248
Q5AFA2	Extracellular glycosidase CRH11	46.7	215.74	14.35	1	5	5	6	453	4.96	0.001494
Q9B8D8	Cytochrome c oxidase subunit 2	29.8	214.08	17.94	12	3	3	5	262	4.68	0.002153
P43060	Phosphoribosylaminoimidazole-succinocarboxamide synthase	32.9	213.56	19.24	2	3	3	4	291	5.50	0.001551
P30418	Glycylpeptide N-tetradecanoyltransferase	51.8	210.73	10.42	1	3	3	3	451	6.48	0.000750
P46585	Ribose-phosphate pyrophosphokinase 1	35.3	209.74	15.89	1	4	4	5	321	6.79	0.001757
Q9P8P7	Ribosomal RNA small subunit methyltransferase NEP1	29.5	208.32	30.34	2	6	6	6	267	8.43	0.002535
C4YJI1	Altered inheritance of mitochondria protein 36, mitochondrial	34.7	208.22	26.03	3	7	7	7	292	8.34	0.002705
Q5ANE3	Non-classical export protein 102	18.1	206.99	18.82	1	2	2	3	170	9.01	0.001991
Q5ADT9	37S ribosomal protein S10, mitochondrial	27.4	202.93	21.79	1	4	4	4	234	9.48	0.001929
Q59SU1	Candidapepsin-9	58.4	202.51	9.93	2	6	6	6	544	5.25	0.001244
P83783	Adenosylhomocysteinase	49.0	200.46	18.44	3	6	6	7	450	5.59	0.001755
P22274	ADP-ribosylation factor	20.2	199.17	27.93	1	1	3	4	179	5.40	0.002521
P78590	Elongation factor 1-beta	23.5	197.00	23.47	1	3	3	4	213	4.40	0.002119
Q59X23	Dolichyl-phosphate-mannose--protein mannosyltransferase 4	86.6	193.57	6.09	1	4	4	4	755	8.87	0.000598

Q5AK59	ATP-dependent RNA helicase HAS1	63.0	193.53	16.99	30	8	8	8	565	8.91	0.001597
Q5AF03	Glyoxalase 3	25.8	189.50	22.03	1	3	3	4	236	4.83	0.001912
Q9Y7F0	Peroxiredoxin TSA1	21.8	186.20	23.47	4	4	4	4	196	5.06	0.002302
Q5AGZ9	RuvB-like helicase 2	54.5	184.30	7.63	6	3	3	4	498	5.15	0.000906
P22011	Peptidyl-prolyl cis-trans isomerase	17.6	182.21	27.16	2	3	4	5	162	7.97	0.003482
P83780	Glucose-6-phosphate isomerase	61.1	180.95	20.18	5	9	9	9	550	6.40	0.001846
Q59P03	NADH-cytochrome b5 reductase 1	32.5	179.06	20.07	2	6	6	7	294	8.10	0.002686
Q5APF2	GMP synthase [glutamine-hydrolyzing]	58.8	178.33	17.55	8	7	7	7	530	5.92	0.001490
P34725	Phospho-2-dehydro-3-deoxyheptonate aldolase, phenylalanine-inhibited	40.7	176.21	18.21	3	4	5	5	368	7.11	0.001533
Q5A4Q1	Adenylate kinase	27.6	175.83	21.69	16	5	5	5	249	8.10	0.002265
O13432	Phenylalanine--tRNA ligase beta subunit	66.8	175.60	9.80	2	5	5	6	592	5.35	0.001143
Q5A455	Protein transport protein SEC23	85.6	170.89	5.91	5	4	4	4	762	5.71	0.000592
A0A1D8PH78	Farnesyl pyrophosphate synthase	40.7	170.38	11.11	1	3	3	4	351	4.98	0.001286
P46250	SEC14 cytosolic factor	34.7	170.01	20.27	5	5	5	5	301	6.40	0.001874
Q5ABV6	SWI5-dependent HO expression protein 3	59.2	166.25	7.71	3	3	3	3	519	9.20	0.000652
Q5A4X0	E3 ubiquitin-protein ligase BRE1	78.5	164.92	4.70	1	3	3	3	681	6.14	0.000497
G1UB11	C-22 sterol desaturase ERG5	59.6	164.04	17.79	1	7	7	7	517	6.64	0.001528
Q5ACI8	Peptidyl-prolyl cis-trans isomerase D	40.7	163.11	13.55	2	3	4	4	369	6.39	0.001223
A0A1D8PI71	Squalene synthase ERG9	51.2	161.55	18.75	2	9	9	10	448	6.84	0.002518
Q9P975	Eukaryotic translation initiation factor 4E	24.2	160.82	16.27	1	3	3	4	209	5.26	0.002159
Q5A1B0	Sterol-4-alpha-carboxylate 3-dehydrogenase ERG26, decarboxylating	39.2	160.35	11.71	2	5	5	5	350	6.73	0.001612
Q59KG2	Respiratory growth induced protein 1	23.6	158.91	20.40	4	4	4	5	201	5.82	0.002807
Q5A860	Translationally-controlled tumor protein homolog	18.5	158.76	29.34	2	3	3	3	167	4.46	0.002027
Q59S06	Nucleolar protein 58	57.1	158.53	11.82	9	5	5	7	516	8.31	0.001531
Q59V93	Very-long-chain 3-oxoacyl-CoA reductase	38.3	157.49	13.18	1	5	5	5	349	9.57	0.001616
P29717	Glucan 1,3-beta-glucosidase	50.0	157.22	13.47	3	4	4	5	438	5.64	0.001288
Q59MV9	Flavohemoprotein	45.8	153.77	21.86	1	6	6	7	398	5.83	0.001984
Q59Y31	Yeast-form wall Protein 1	54.2	153.40	4.50	1	2	2	7	533	4.81	0.001482
P52498	Ras-related protein RSR1	27.6	152.26	30.24	2	6	6	6	248	5.21	0.002730
Q5AP65	Protein FMP52, mitochondrial	24.3	152.26	29.69	1	6	6	6	229	8.98	0.002956
Q5AJC0	Extracellular glycosidase UTR2	51.7	151.54	9.15	1	4	4	4	470	4.73	0.000960
Q5A7M3	Kynurenine 3-monooxygenase	51.5	150.29	12.50	1	4	4	4	456	7.50	0.000990

Q5AI21	Translocation protein SEC62	33.3	149.54	15.02	2	4	4	4	293	9.51	0.001540
O93807	Tubulin gamma chain	56.4	149.52	2.99	1	1	1	1	502	4.96	0.000225
P34948	Mannose-6-phosphate isomerase	48.8	148.41	9.98	1	4	4	5	441	5.33	0.001279
G1UB61	Septin CDC11	46.7	147.95	8.96	1	3	3	3	402	5.07	0.000842
P0CU36	Ribosome biogenesis protein C3_06160C_A	29.6	145.97	18.77	10	5	5	6	261	10.18	0.002594
P30572	Chitin synthase 2	115.5	144.07	4.36	1	3	3	4	1009	5.73	0.000447
P82612	Phosphoglycerate mutase	27.4	143.98	21.77	1	3	3	3	248	6.16	0.001365
P87023	Beta-glucan synthesis-associated protein KRE6	82.4	143.97	8.24	1	3	4	5	740	4.78	0.000762
Q59NP1	Copper transport protein CTR1	27.8	139.40	24.70	1	4	4	4	251	6.93	0.001798
Q59Q46	Inosine-5'-monophosphate dehydrogenase	56.2	137.57	3.07	1	1	1	2	521	6.55	0.000433
Q59ZV5	Eukaryotic translation initiation factor 3 subunit G	30.7	136.69	12.90	3	3	3	3	279	9.06	0.001213
Q9HFQ7	60S acidic ribosomal protein P1-A	11.0	136.02	20.75	1	2	2	2	106	4.02	0.002129
Q59MA9	Clustered mitochondria protein homolog	155.7	135.71	4.55	1	5	5	5	1363	5.39	0.000414
P43063	Cyclin-dependent kinase 1	36.6	135.21	13.25	18	4	4	4	317	6.61	0.001424
P87219	Sorbose reductase SOU1	30.0	133.28	13.17	1	2	2	2	281	5.30	0.000803
Q5ACU6	ATP-dependent rRNA helicase RRP3	59.8	131.89	9.93	7	4	4	4	534	9.73	0.000845
A0A1D8PLI2	Isopentenyl-diphosphate delta-isomerase	32.3	131.39	19.37	1	3	3	3	284	4.97	0.001192
P87024	Beta-glucan synthesis-associated protein SKN1	83.7	131.25	7.33	1	2	3	4	737	5.15	0.000612
P47831	60S ribosomal protein L28 (Fragment)	6.6	129.50	46.77	1	3	3	6	62	9.09	0.010918
C4YKP5	Increased recombination centers protein 22-1	25.9	128.73	22.18	5	3	3	4	239	5.68	0.001888
Q5AQ57	37S ribosomal protein S25, mitochondrial	34.2	126.39	14.24	1	4	4	4	295	8.03	0.001530
Q5A1D5	FACT complex subunit SPT16	121.3	125.75	7.08	1	6	6	6	1060	5.16	0.000639
Q5APC0	Golgi apparatus membrane protein TVP18	18.8	124.82	15.03	2	2	2	2	173	7.74	0.001304
Q5AEM8	Presequence translocated-associated motor subunit PAM17, mitochondrial	21.0	122.77	20.00	1	3	3	4	185	10.14	0.002439
Q59WU0	Probable lysine/arginine permease CAN2	63.1	122.59	3.87	3	2	2	2	568	6.98	0.000397
Q59W44	Mitochondrial import inner membrane translocase subunit TIM50	54.2	121.53	15.99	2	7	7	7	469	6.65	0.001684
Q5ABP8	Protein ROT1	29.9	121.22	12.31	2	3	3	3	260	7.80	0.001302
A0A1D8PNZ7	Glycerophosphocholine phosphodiesterase GDE1	130.8	119.61	2.41	1	2	2	2	1162	6.30	0.000194
Q59VP7	Ribosome biogenesis protein ERB1	97.3	119.08	4.24	12	3	3	3	849	4.74	0.000399
Q9UVX1	Lysophospholipase 3	81.4	118.13	4.51	1	3	3	3	754	4.84	0.000449
O13359	Kexin	105.1	113.42	4.37	1	4	4	4	938	5.03	0.000481
Q59ZH9	ATP-dependent RNA helicase MAK5	88.0	113.27	6.52	8	5	5	5	782	6.04	0.000721

Q9HEW1	cAMP-dependent protein kinase regulatory subunit	50.3	113.11	6.10	1	2	2	2	459	5.48	0.000492
Q59WF4	Alpha-1,2-mannosyltransferase MNN2	69.1	112.56	6.53	1	3	3	3	597	6.27	0.000567
Q9P8E3	Protein transport protein SEC61 subunit alpha	52.5	111.80	8.14	2	3	3	4	479	8.95	0.000942
P0CB63	Golgi to ER traffic protein 2	33.6	111.72	8.72	2	2	2	2	298	5.52	0.000757
Q59S72	GDP-Man:Man(3)GlcNAc(2)-PP-Dol alpha-1,2-mannosyltransferase	70.9	110.56	4.43	1	2	2	2	609	8.53	0.000371
Q9B8C9	NADH-ubiquinone oxidoreductase chain 5	61.5	109.53	7.97	2	4	4	4	552	6.74	0.000818
Q9P844	40S ribosomal protein S21	9.6	108.86	28.74	2	2	2	2	87	8.15	0.002594
Q9UVL1	Non-histone chromosomal protein 6	10.5	106.56	28.26	2	2	2	3	92	9.70	0.003679
O42617	Poly(A) polymerase PAPalpha	63.2	105.78	5.38	3	2	2	2	558	8.07	0.000404
Q5AG77	Amino-acid permease GAP1	63.9	104.38	2.75	1	1	1	1	582	9.17	0.000194
Q5ANP2	Nascent polypeptide-associated complex subunit alpha	19.5	104.15	14.04	3	2	2	2	178	4.82	0.001268
P53697	Mannan polymerase complex subunit MNN9	42.8	103.04	11.68	1	3	3	4	368	9.14	0.001226
C4YH95	tRNA (guanine(37)-N1)-methyltransferase	51.8	102.72	8.02	2	2	2	2	449	8.02	0.000503
P0C8K9	Cytochrome c oxidase subunit 1	58.7	101.69	3.01	1	1	1	2	531	6.37	0.000425
Q5AJC1	Vacuolar protein-sorting protein BRO1	108.8	99.53	2.75	1	2	2	2	945	5.52	0.000239
Q59KM8	Cell cycle protein kinase DBF2	82.3	99.24	4.79	1	3	3	3	710	9.13	0.000477
Q5A368	Lysophospholipase NTE1	155.6	98.87	2.16	1	3	3	3	1386	8.38	0.000244
P23286	Calmodulin	16.5	98.17	20.13	9	2	2	2	149	4.41	0.001514
Q5AF95	ATP-dependent RNA helicase DBP4	86.7	97.53	4.44	1	3	3	3	765	8.15	0.000442
O94072	V-type proton ATPase subunit E	25.4	97.12	10.62	2	2	2	2	226	5.40	0.000998
Q59KJ7	Alpha-1,2-mannosyltransferase MNN21	76.9	97.12	2.12	1	1	1	1	660	7.11	0.000171
Q5AC48	Actin-related protein 4	52.6	96.63	7.48	1	2	2	2	468	5.57	0.000482
Q5AAR0	Transcription factor IWS1	45.8	95.49	10.25	1	3	3	3	400	8.12	0.000846
Q59XM1	Exocyst complex component EXO84	88.5	93.85	3.16	1	2	2	2	791	8.98	0.000285
Q5AL52	Formin BNI1	196.7	91.99	1.44	1	2	2	2	1732	6.15	0.000130
C4YS59	Vacuolar membrane protease	93.7	90.92	1.79	2	1	1	2	837	5.29	0.000270
P39827	Cell division control protein 10	40.7	89.75	5.32	1	1	1	1	357	7.11	0.000316
Q9B8D1	NADH-ubiquinone oxidoreductase chain 3	14.7	88.52	19.38	1	2	2	2	129	5.06	0.001749
Q5A1L6	Major glycerophosphoinositol permease GIT3	59.4	87.87	7.10	1	2	2	3	535	8.06	0.000633
Q59W33	Glycerol-3-phosphate dehydrogenase [NAD(+)] 2	40.8	86.99	15.90	1	5	5	5	371	5.31	0.001521
Q59KF3	AdoMet-dependent rRNA methyltransferase SPB1	96.8	86.85	2.13	3	2	2	2	845	5.76	0.000267
Q59LV5	Phosphatidylethanolamine N-methyltransferase	102.4	86.62	3.83	6	4	4	4	888	7.74	0.000508

Q5AQ76	Protein transport protein SEC24	102.0	86.44	2.48	2	2	2	2	928	5.40	0.000243
Q5AAI8	Nucleosome assembly protein 1	49.5	85.99	6.21	1	3	3	3	435	4.31	0.000778
P33153	GTP-binding RHO-like protein	39.1	84.38	4.62	1	1	1	1	346	8.43	0.000326
Q59L13	Eukaryotic translation initiation factor 6	26.4	83.63	15.51	7	3	3	3	245	4.58	0.001381
Q5AB93	Protein PNS1	58.0	82.05	6.56	2	3	3	3	518	8.46	0.000653
P43068	Mitogen-activated protein kinase MKC1	58.2	81.00	2.20	2	1	1	1	501	5.02	0.000225
O42816	Signal recognition particle 54 kDa protein homolog	60.7	80.41	5.22	3	3	3	3	556	9.13	0.000609
O13427	Low-specificity L-threonine aldolase	41.8	78.94	4.01	1	1	1	1	374	6.42	0.000302
Q59TU0	Nascent polypeptide-associated complex subunit beta	17.0	76.53	13.38	4	2	2	2	157	5.71	0.001437
Q5A310	ISWI chromatin-remodeling complex ATPase ISW2	123.0	75.88	2.08	1	2	2	2	1056	6.73	0.000214
Q5AAF4	Formin BNR1	168.5	75.71	1.14	1	1	1	1	1485	8.44	0.000076
Q9HGY5	Negative regulator of the PHO system	37.3	75.55	4.91	1	1	1	1	326	6.86	0.000346
P43065	Saccharopine dehydrogenase [NAD(+), L-lysine-forming]	42.4	74.47	12.57	1	4	4	4	382	5.44	0.001181
P31225	Corticosteroid-binding protein	55.5	73.82	8.38	1	4	4	4	489	5.29	0.000923
O14427	Serine/threonine-protein kinase CLA4	106.8	73.27	2.06	2	2	2	2	971	9.35	0.000232
Q00312	Transcription factor RBF1	59.4	72.89	2.85	2	1	1	1	527	8.66	0.000214
Q92410	Alpha,alpha-trehalose-phosphate synthase [UDP-forming]	54.4	72.68	7.32	1	4	4	4	478	6.23	0.000944
O93875	Delta(7)-sterol 5(6)-desaturase	45.4	72.61	5.18	3	2	2	2	386	6.80	0.000585
Q5AIA1	Glucan 1,3-beta-glucosidase 2	54.5	71.88	5.64	1	2	2	2	479	5.53	0.000471
Q59KI4	Chromatin-remodeling ATPase INO80	158.7	71.62	1.73	1	2	2	2	1387	8.09	0.000163
Q5ABC5	Phosphatidylserine decarboxylase proenzyme 1, mitochondrial	67.5	71.56	5.76	1	2	2	2	590	9.48	0.000382
Q59SI2	Mitochondrial import inner membrane translocase subunit TIM14	17.3	71.38	26.11	2	2	2	3	157	9.64	0.002156
Q5ABD0	Vacuolar-sorting protein SNF7	25.9	69.86	9.73	2	2	2	2	226	4.88	0.000998
A0A1D8PEL1	Mevalonate kinase	47.0	69.41	5.80	1	2	2	2	431	5.68	0.000524
A0A1D8PC43	Diphosphomevalonate decarboxylase	39.5	69.38	5.25	1	2	2	2	362	6.46	0.000623
Q9HFQ6	60S acidic ribosomal protein P1-B	10.7	69.31	14.81	1	1	1	1	108	3.93	0.001045
Q59QC6	Assembly factor CBP4	17.1	69.12	17.36	1	2	2	2	144	8.09	0.001567
Q5ACM4	Pre-rRNA-processing protein PNO1	30.6	69.02	7.61	7	2	2	2	276	8.90	0.000818
Q5A9Z6	ATP-dependent RNA helicase FAL1	45.5	68.78	3.26	1	1	1	1	399	8.02	0.000283
Q5A3V6	3,4-dihydroxy-2-butanone 4-phosphate synthase	22.6	67.70	9.80	1	2	2	2	204	5.44	0.001106
Q9Y7C4	ATP-dependent RNA helicase CHR1	65.4	67.70	5.71	5	3	3	3	578	9.23	0.000586
Q5AED9	Branchpoint-bridging protein	50.0	66.90	3.30	1	1	1	1	455	9.42	0.000248

Q59VP2	Histone H2A.2	13.8	66.24	25.19	15	1	2	2	131	10.24	0.001722
Q5A888	3-keto-steroid reductase ERG27	39.0	64.84	5.78	2	2	2	2	346	7.91	0.000652
Q5APM7	ATP-dependent RNA helicase MSS116, mitochondrial	76.7	64.81	4.34	1	2	2	2	668	9.48	0.000338
Q59LP6	Altered inheritance of mitochondria protein 11	18.6	64.76	9.09	1	1	1	1	165	5.35	0.000684
Q5ACK7	ATP-dependent RNA helicase DRS1	69.3	64.32	1.96	1	1	1	1	613	6.19	0.000184
Q59PD6	Spindle assembly checkpoint component MAD1	82.2	64.29	2.30	1	1	1	1	696	6.93	0.000162
P87078	DNA topoisomerase 2	165.3	64.23	2.33	1	3	3	3	1461	6.40	0.000232
P56091	Galactokinase	57.0	63.92	2.33	1	1	1	1	515	5.54	0.000219
O14415	Signal recognition particle SEC65 subunit	31.1	63.64	6.91	1	2	2	2	275	7.53	0.000821
Q59VX8	Septation protein 7	75.7	63.28	4.48	1	3	3	3	670	6.02	0.000505
Q5A216	Probable kinetochore protein NDC80	92.4	62.80	1.78	1	1	1	1	788	5.45	0.000143
Q59X67	Enhanced filamentous growth protein 1	59.6	62.14	2.36	2	1	1	1	550	9.36	0.000205
Q5ALV2	Actin cytoskeleton-regulatory complex protein SLA1	138.8	60.70	1.83	1	2	2	2	1257	6.05	0.000180
Q5AK66	Phosphatidylserine decarboxylase proenzyme 2	121.6	60.37	1.78	1	2	2	2	1070	6.92	0.000211
Q5AAJ7	SWR1-complex protein 4	74.0	58.04	1.42	1	1	1	1	635	8.19	0.000178
Q3MNT0	Transcription elongation factor SPT6	162.0	57.73	1.21	1	1	1	1	1401	5.24	0.000081
O74933	UDP-N-acetylglucosamine pyrophosphorylase	54.6	56.83	5.97	1	2	2	2	486	6.28	0.000464
Q5AHJ5	Transcription factor TBF1	100.8	56.25	1.13	1	1	1	1	886	4.67	0.000127
P30574	Carboxypeptidase Y	61.0	55.92	4.98	1	3	3	3	542	5.47	0.000624
P52497	Carbon catabolite-derepressing protein kinase	70.0	55.86	4.19	2	2	2	2	620	7.91	0.000364
Q59PR3	ATP-dependent RNA helicase DBP8	48.8	55.61	4.32	13	1	2	2	440	8.91	0.000513
Q5AI97	Mitochondrial genome maintenance protein MGM101	30.7	54.80	5.45	2	1	1	1	275	9.10	0.000410
O74712	Histidine biosynthesis trifunctional protein	91.8	54.63	1.91	1	2	2	2	838	5.38	0.000269
Q5ALL3	tRNA-dihydrouridine(47) synthase [NAD(P)(+)]	69.6	53.92	2.12	1	1	1	1	613	7.69	0.000184
P53716	Uncharacterized protein CAWG_04269, mitochondrial	25.0	53.23	5.83	1	1	1	1	223	8.63	0.000506
P53705	Bud site selection protein BUD4	192.8	51.34	0.64	1	1	1	1	1709	6.04	0.000066
Q9P836	60S ribosomal protein L37 (Fragment)	9.9	51.19	18.18	1	2	2	2	88	11.63	0.002564
Q04782	Lanosterol synthase	83.7	50.99	2.06	1	1	1	1	728	5.92	0.000155
Q92210	Phosphoribosylaminoimidazole carboxylase	62.4	50.69	3.35	1	2	2	2	568	6.49	0.000397
Q5AP53	Serine/threonine-protein kinase CBK1	84.4	50.53	3.42	1	2	2	2	732	6.40	0.000308
O74226	Cell wall synthesis protein KRE9	29.1	50.48	5.54	1	1	1	1	271	8.18	0.000416
P84285	Sterol O-acyltransferase 2	71.3	50.15	1.64	1	1	1	1	609	8.46	0.000185

Q5AD77	Sorting nexin-4	71.8	50.10	2.06	1	1	1	1	630	5.63	0.000179
P83775	Putative NADPH-dependent methylglyoxal reductase GRP2	37.6	49.06	3.23	1	1	1	1	341	6.44	0.000331
P40954	Chitinase 3	60.0	48.99	4.23	1	1	1	1	567	4.91	0.000199
Q59MF9	Conserved oligomeric Golgi complex subunit 6	88.4	48.37	2.08	1	1	2	2	771	5.03	0.000293
O74270	Origin recognition complex subunit 1	91.2	48.31	3.35	1	2	2	2	805	6.25	0.000280
A0A1D8PCB9	C-8 sterol isomerase ERG2	24.5	48.21	5.53	1	1	1	1	217	5.48	0.000520
Q5A2A2	Mitochondrial homologous recombination protein 1	28.3	47.46	4.60	1	1	1	1	239	8.50	0.000472
Q5ADW3	Cullin-associated NEDD8-dissociated protein 1	134.4	47.15	1.26	1	1	1	1	1195	4.96	0.000094
Q59WH0	Transcriptional adapter 2	51.1	47.02	2.25	1	1	1	1	445	7.46	0.000254
Q59X38	Pescadillo homolog	67.8	46.78	5.45	3	3	3	3	587	6.19	0.000577
Q59PP6	Mediator of RNA polymerase II transcription subunit 16	117.7	46.36	0.85	1	1	1	2	1053	7.61	0.000214
Q5ACM9	Eukaryotic translation initiation factor 3 subunit J	32.0	46.00	4.21	1	1	1	1	285	4.98	0.000396
Q5AD56	General negative regulator of transcription subunit 3	85.7	45.65	1.99	1	1	1	1	752	5.57	0.000150
P52499	Protein RCC1	51.3	44.81	2.80	1	1	1	1	464	5.43	0.000243
Q5AJS6	Multiple RNA-binding domain-containing protein 1	94.9	44.72	1.55	1	1	1	1	841	5.62	0.000134
Q5A3P6	Serine/threonine-protein kinase PKH2	106.5	44.34	1.80	1	1	1	1	947	8.28	0.000119
A0A1D8PJ25	Delta(24(24(1)))-sterol reductase	54.8	44.18	4.26	1	2	2	2	469	7.06	0.000481
Q9HFQ5	60S acidic ribosomal protein P2-A	10.9	43.72	12.96	1	1	1	1	108	3.93	0.001045
Q5APQ8	Putative alpha-1,3-mannosyltransferase MNN12	97.5	43.57	1.57	1	1	1	1	828	8.16	0.000136
Q5ANB2	ATP-dependent RNA helicase DBP10	103.5	43.35	2.64	1	2	2	2	908	9.07	0.000249
Q5ANL6	13 kDa ribonucleoprotein-associated protein	13.6	43.03	26.98	4	2	2	2	126	7.97	0.001791
Q5AEK8	Delta 8-(E)-sphingolipid desaturase	67.3	42.94	1.37	1	1	1	1	584	6.96	0.000193
Q59NX9	Diphthine methyl ester synthase 1	33.9	42.80	6.35	1	2	2	2	299	4.87	0.000755
C4YPQ0	Altered inheritance of mitochondria protein 24, mitochondrial	52.7	42.80	1.68	2	1	1	1	475	9.91	0.000238
Q9HFQ4	60S acidic ribosomal protein P2-B	11.2	42.23	10.81	1	1	1	1	111	4.07	0.001016
A0A1D8PN88	Amino-acid permease GAP3	65.5	41.70	1.84	1	1	1	1	599	8.43	0.000188
A0A1D8PK89	General amino-acid permease GAP2	63.5	41.51	1.36	1	1	1	1	588	7.77	0.000192
P53698	Cytochrome c	12.2	41.49	19.09	4	2	2	3	110	9.66	0.003077
Q5ALL8	FACT complex subunit POB3	60.9	41.48	5.20	1	2	2	2	538	4.83	0.000419
Q5AHG6	Serine/threonine-protein kinase SCH9	88.7	41.39	1.27	1	1	1	1	787	6.20	0.000143
Q5A1Q5	Probable kinetochore protein NUF2	56.6	40.95	1.86	1	1	1	1	485	5.55	0.000233
C4YTG0	Pheromone-processing carboxypeptidase KEX1	78.7	40.66	1.28	2	1	1	1	702	5.12	0.000161

Q12572	L-2-aminoadipate reductase large subunit	154.6	40.58	0.79	1	1	1	1	1391	6.87	0.000081
Q5AD51	Ergosterol biosynthetic protein 28	16.0	39.43	7.97	1	1	1	1	138	9.31	0.000818
Q59NY7	Coupling of ubiquitin conjugation to ER degradation protein 1	21.2	39.21	5.29	1	1	1	1	189	7.15	0.000597
Q5AHC2	Mitochondrial inner membrane i-AAA protease complex subunit MGR1	41.5	38.99	2.45	1	1	1	1	368	5.41	0.000307
P87185	Cysteine desulfurase, mitochondrial	53.5	38.91	4.92	3	2	2	2	488	8.18	0.000462
Q5A477	GDP-mannose transporter	41.2	38.54	2.70	1	1	1	1	371	9.36	0.000304
Q59N80	Inosine triphosphate pyrophosphatase	22.0	38.04	4.46	1	1	1	1	202	5.66	0.000559
O93831	Rab proteins geranylgeranyltransferase component A	72.2	36.05	1.41	1	1	1	1	640	4.81	0.000176
Q9UWF6	Lysophospholipase 1	66.4	35.95	2.15	1	1	1	1	605	4.87	0.000186
Q5AEF2	Protein transport protein SEC13	33.0	35.92	3.69	1	1	1	1	298	5.62	0.000379
Q59S50	ATP-dependent RNA helicase DBP7	81.1	34.98	1.38	3	1	1	1	727	8.95	0.000155
P48990	Mitochondrial import inner membrane translocase subunit TIM54	46.1	34.91	3.50	1	1	1	1	400	5.52	0.000282
Q5AKU3	CAP1-binding-protein	73.9	34.62	1.87	1	1	1	1	643	5.92	0.000175
P23316	Chitin synthase 1	88.2	33.89	2.45	1	1	1	1	776	8.63	0.000145
Q59RP7	54S ribosomal protein L4, mitochondrial	36.3	33.65	2.59	1	1	1	1	309	5.96	0.000365
C4YF50	NAD(P)H-hydrate epimerase	28.7	31.95	6.59	2	1	1	1	258	6.70	0.000437
Q9B8D6	NADH-ubiquinone oxidoreductase chain 1	34.8	30.88	2.52	2	1	1	1	317	7.17	0.000356
O93803	mRNA-capping enzyme subunit beta	58.8	30.40	2.12	1	1	1	1	520	7.87	0.000217
Q59LF2	Alpha-1,3/1,6-mannosyltransferase ALG2	50.4	29.56	2.26	1	1	1	1	443	8.31	0.000255
Q5A761	CCR4-Not complex 3'-5'-exoribonuclease subunit Ccr4	90.3	28.89	1.52	1	1	1	1	787	7.20	0.000143
Q5APA2	Golgi apparatus membrane protein TVP23	29.9	28.58	5.62	1	1	1	1	267	6.19	0.000423
Q5A7S7	Fork-head transcriptional regulator 2	76.3	28.38	1.02	2	1	1	1	687	9.42	0.000164
Q5AK62	Virulence protein SSD1	141.2	27.76	0.55	1	1	1	2	1274	7.36	0.000177
Q5A744	Protein SDS23	67.5	27.19	1.91	1	1	1	1	628	8.44	0.000180
Q5AGA0	Putative alpha-1,3-mannosyltransferase MNN1	94.2	26.22	1.38	1	1	1	1	800	6.93	0.000141
Q59ZW9	Mitochondrial import inner membrane translocase subunit TIM16	13.3	26.16	11.57	1	1	1	1	121	7.24	0.000932
P46588	DNA polymerase delta catalytic subunit	118.8	25.43	1.64	1	1	1	2	1038	8.21	0.000217
Q5AP90	Alpha-1,2-mannosyltransferase MNN23	69.6	24.70	1.32	1	1	1	1	606	5.40	0.000186
G1UB37	Major facilitator superfamily multidrug transporter FLU1	67.6	24.34	1.64	1	1	1	1	610	6.65	0.000185
A0A1D8PNP3	Amino-acid permease GAP6	61.8	24.18	1.58	1	1	1	1	568	8.72	0.000199
Q59U67	Transcription initiation factor TFIID subunit 4	39.6	22.97	2.75	1	1	1	1	363	9.13	0.000311
Q5A599	Histidine protein kinase NIK1	118.9	22.77	1.30	1	1	1	1	1081	5.80	0.000104

Q59NG5	Crossover junction endonuclease MUS81	70.6	22.39	1.47	1	1	1	1	614	9.28	0.000184
Q92207	Mitogen-activated protein kinase HOG1	42.9	21.62	2.39	3	1	1	1	377	5.19	0.000299
P43102	Ubiquitin-conjugating enzyme E2 4	16.3	18.27	7.48	2	1	1	1	147	7.40	0.000767
Q00313	DNA topoisomerase 1	90.4	16.99	1.03	1	1	1	1	778	9.03	0.000145
C4YRP9	Respiratory supercomplex factor 1, mitochondrial	18.1	16.38	18.06	3	2	2	2	155	9.44	0.001456
**EV_{CASP}**											
Q92211	Glyceraldehyde-3-phosphate dehydrogenase	35.8	8842.73	86.27	41	20	26	293	335	7.12	0.090136
P83779	Pyruvate decarboxylase	62.4	6749.27	61.38	4	26	26	164	567	5.58	0.029808
P25997	Elongation factor 3	116.9	4080.58	50.86	5	34	41	101	1050	5.73	0.009913
P46587	Heat shock protein SSA2	70.0	3885.96	63.57	7	14	30	123	645	5.06	0.019653
P41797	Heat shock protein SSA1	70.3	3688.71	50.46	11	8	25	114	656	5.17	0.017909
P43067	Alcohol dehydrogenase 1	36.9	3342.45	60.00	2	14	18	111	350	6.44	0.032684
P87222	Heat shock protein SSB1	66.4	3193.42	58.08	9	24	24	81	613	5.38	0.013618
P28877	Plasma membrane ATPase 1	97.4	2881.77	36.09	8	17	25	73	895	4.96	0.008406
P34731	Fatty acid synthase subunit beta	227.8	2669.06	32.84	4	47	47	68	2037	6.02	0.003440
P0CY35	Elongation factor 1-alpha 1	50.0	2489.69	48.69	22	6	18	122	458	9.03	0.027452
Q9URB4	Fructose-bisphosphate aldolase	39.2	2354.57	70.47	3	18	18	60	359	6.06	0.017224
P43098	Fatty acid synthase subunit alpha	207.5	2133.65	26.53	8	40	40	50	1885	5.62	0.002734
C4YJQ8	Elongation factor 2	93.3	2057.45	42.04	12	21	32	58	842	6.47	0.007099
P83776	Hexokinase-2	53.4	1937.11	68.39	3	22	24	52	484	5.55	0.011072
Q96VB9	Heat shock protein homolog SSE1	78.5	1934.31	60.20	6	33	33	51	701	5.30	0.007498
P14235	Actin	41.7	1516.03	50.53	30	15	15	37	376	5.69	0.010141
P30575	Enolase 1	47.2	1379.32	49.55	13	15	15	31	440	5.81	0.007261
P43076	pH-responsive protein 1	59.4	1222.20	43.25	2	20	21	26	548	5.07	0.004890
P83784	Heat shock protein SSC1, mitochondrial	69.7	1122.62	37.65	1	20	20	26	648	5.60	0.004135
P46614	Pyruvate kinase	55.4	1095.69	47.02	14	15	15	22	504	6.99	0.004498
P46598	Heat shock protein 90 homolog	80.8	1092.88	37.34	7	24	24	29	707	4.88	0.004227
Q5A8K2	Alanine--tRNA ligase	108.2	1066.03	31.17	3	24	24	29	969	6.07	0.003084
O94039	Transketolase 1	73.7	1058.36	36.93	1	19	19	31	677	5.82	0.004719
P46273	Phosphoglycerate kinase	45.2	963.97	67.15	20	23	23	30	417	6.48	0.007414
P82610	5-methyltetrahydropteroyltriglutamate--homocysteine methyltransferase	85.6	959.98	29.47	2	16	16	21	767	5.60	0.002822
Q9Y872	Sulfate adenylyltransferase	58.8	946.11	44.21	20	19	19	27	527	6.40	0.005280

Q9HGT6	Serine--tRNA ligase, cytoplasmic	53.0	903.91	50.22	4	21	21	25	462	5.73	0.005577
O42817	40S ribosomal protein S0	28.7	903.49	61.30	33	12	12	30	261	4.91	0.011846
Q59PT0	V-type proton ATPase subunit B	57.2	887.03	43.16	5	2	18	23	512	5.03	0.004629
P83774	Guanine nucleotide-binding protein subunit beta-like protein	34.5	880.67	52.68	1	12	12	23	317	6.54	0.007477
Q59KZ1	Aminopeptidase 2	104.3	872.00	34.20	2	27	27	27	924	5.36	0.003011
P40910	40S ribosomal protein S1	29.0	858.36	54.69	53	15	15	27	256	10.04	0.010869
C4YKT4	Ras-like protein 1	32.2	824.75	35.76	2	6	6	14	288	4.67	0.005010
P47837	40S ribosomal protein S4	29.2	818.36	45.42	1	9	13	29	262	10.21	0.011407
P43071	Multidrug resistance protein CDR1	169.8	813.08	14.06	6	14	16	19	1501	6.98	0.001305
Q8NJN3	Acetyl-coenzyme A synthetase 2	73.8	811.64	31.15	13	17	19	26	671	6.13	0.003993
P87206	ATP-dependent RNA helicase eIF4A	44.6	783.42	49.12	40	15	15	17	397	5.36	0.004413
P82611	Aconitate hydratase, mitochondrial	84.2	770.87	33.46	5	17	17	20	777	6.39	0.002653
O74261	Heat shock protein 60, mitochondrial	60.1	760.38	42.76	6	17	17	20	566	5.30	0.003642
Q59KI0	UTP--glucose-1-phosphate uridylyltransferase	55.5	755.22	34.40	3	12	12	15	500	6.73	0.003092
Q5AML1	Eukaryotic translation initiation factor 3 subunit C	99.8	753.47	28.38	5	22	22	25	874	5.15	0.002948
O74676	ABC transporter CDR4	169.2	739.37	17.85	5	18	20	20	1490	7.65	0.001383
P83778	Malate dehydrogenase, cytoplasmic	36.0	734.30	42.73	1	11	11	14	337	5.62	0.004281
P83782	Cytochrome b-c1 complex subunit 2, mitochondrial	39.5	723.32	66.58	1	16	16	16	374	5.57	0.004409
P30573	Chitin synthase 3	136.1	713.15	22.84	9	21	21	23	1213	7.17	0.001954
Q59PL9	Eukaryotic translation initiation factor 3 subunit A	106.0	709.98	23.87	5	18	18	20	930	8.31	0.002216
P83777	Inorganic pyrophosphatase	32.1	705.43	57.64	9	11	11	16	288	5.26	0.005725
P43084	Probable NADPH dehydrogenase	46.0	698.62	45.21	1	2	15	19	407	6.39	0.004811
Q9P843	60S ribosomal protein L27	15.5	676.61	47.79	1	7	7	20	136	10.18	0.015155
Q5AKA5	Cys-Gly metallodipeptidase DUG1	53.6	672.82	32.78	1	11	11	16	485	5.24	0.003400
P52495	Ubiquitin-activating enzyme E1 1	114.2	663.46	22.53	2	17	17	18	1021	5.01	0.001817
O94049	Acetyl-coenzyme A synthetase 1	75.1	643.76	29.63	6	16	18	22	675	6.32	0.003359
P10613	Lanosterol 14-alpha demethylase	60.6	629.22	36.36	3	17	17	20	528	7.17	0.003904
A0A1D8PPK1	Probable NADPH dehydrogenase	46.1	623.58	40.54	1	1	14	18	407	6.05	0.004558
O94200	ATP-dependent 6-phosphofructokinase subunit beta	104.0	615.25	21.99	3	17	17	18	946	6.35	0.001961
P47828	T-complex protein 1 subunit theta	58.9	612.63	28.33	2	11	11	12	540	7.97	0.002290
Q5AIR7	Endo-1,3(4)-beta-glucanase 1	124.0	611.38	17.21	1	16	16	17	1145	5.40	0.001530
Q5AGV4	Eukaryotic translation initiation factor 3 subunit B	84.2	598.49	21.38	5	12	12	16	739	6.39	0.002231

Q5AJD0	ATP-dependent RNA helicase DBP5	60.2	586.28	33.52	25	14	14	16	540	5.76	0.003054
O93827	Mannose-1-phosphate guanyltransferase	40.0	584.85	50.00	15	16	16	20	362	6.30	0.005694
O94201	ATP-dependent 6-phosphofructokinase subunit alpha	108.5	584.69	26.55	1	22	22	22	987	6.62	0.002297
Q5AJB1	V-type proton ATPase catalytic subunit A	67.6	582.92	24.15	6	11	11	12	617	5.27	0.002004
Q00310	Glycolipid 2-alpha-mannosyltransferase 1	50.0	576.06	42.00	1	12	14	15	431	6.90	0.003587
Q5A4E2	ATP-dependent RNA helicase DED1	72.8	575.09	26.64	47	11	13	16	672	8.28	0.002454
O42825	GTP-binding protein RHO1	22.0	566.58	52.53	7	9	9	13	198	5.73	0.006766
P53704	Glutamine--fructose-6-phosphate aminotransferase [isomerizing]	79.2	562.72	22.44	3	12	12	15	713	6.24	0.002168
O13287	6-phosphogluconate dehydrogenase, decarboxylating	56.9	554.71	26.11	4	12	12	16	517	6.57	0.003189
O94083	Eukaryotic translation initiation factor 5A	17.1	542.57	48.10	3	7	7	14	158	5.05	0.009132
Q5AEN1	Cytochrome c peroxidase, mitochondrial	40.7	541.41	37.98	1	9	9	12	366	6.34	0.003379
P10875	Tubulin beta chain	49.9	540.21	36.08	13	11	11	16	449	4.74	0.003672
Q8TGH6	Guanosine-diphosphatase	65.9	533.35	29.05	1	14	14	16	599	5.94	0.002753
Q96W54	40S ribosomal protein S22	14.8	530.56	59.23	9	7	7	12	130	9.88	0.009513
Q5AJY5	1,3-beta-glucanosyltransferase PGA4	49.0	530.51	22.84	1	8	8	10	451	4.74	0.002285
P83781	Mitochondrial outer membrane protein porin	29.7	526.10	46.45	1	10	10	12	282	8.57	0.004385
O13318	pH-responsive protein 2	58.7	522.61	18.38	1	8	9	11	544	4.64	0.002084
O94038	Alcohol dehydrogenase 2	36.8	522.46	36.49	8	8	11	15	348	6.68	0.004442
O13434	Phosphoenolpyruvate carboxykinase [ATP]	60.8	522.31	32.37	2	12	12	14	553	6.60	0.002609
Q9UVJ4	60S ribosomal protein L10a	24.4	521.01	39.17	4	9	9	15	217	9.76	0.007124
O74198	Sterol 24-C-methyltransferase	43.0	518.22	42.55	3	12	12	17	376	6.11	0.004659
O42766	14-3-3 protein homolog	29.5	487.70	38.26	8	8	8	12	264	4.81	0.004684
Q5APD4	Sphingolipid C9-methyltransferase	58.7	486.69	22.42	2	11	11	12	513	6.92	0.002411
P53707	37 kDa cell surface protein	37.0	466.82	30.84	1	10	10	12	321	7.06	0.003853
Q59PR9	Transcriptional regulator HMO1	24.8	456.50	44.39	1	7	7	11	223	9.19	0.005084
Q59S78	Small COPII coat GTPase SAR1	21.5	447.21	51.58	17	7	7	10	190	5.59	0.005424
O94008	60S ribosomal protein L32	14.9	445.41	42.75	1	8	8	16	131	10.54	0.012587
P29717	Glucan 1,3-beta-glucosidase	50.0	442.05	27.17	3	7	7	12	438	5.64	0.002823
Q9P940	Triosephosphate isomerase	26.6	438.23	43.15	4	10	10	16	248	6.01	0.006649
Q5AHH4	Small heat shock protein 21	21.5	438.09	63.49	1	10	10	13	189	5.35	0.007089
Q59LU0	ATP-dependent RNA helicase DBP2	61.2	437.35	29.18	33	13	14	15	562	8.88	0.002751
P34732	Vesicular-fusion protein SEC18	88.9	436.25	19.90	1	12	12	13	794	7.43	0.001687

O13354	Eukaryotic peptide chain release factor GTP-binding subunit	79.0	431.42	18.18	7	9	9	10	715	8.47	0.001441
O13432	Phenylalanine--tRNA ligase beta subunit	66.8	429.67	27.20	2	14	14	18	592	5.35	0.003133
Q96W53	40S ribosomal protein S14	14.4	423.58	40.30	4	5	5	9	134	10.61	0.006922
Q5A0W7	RuvB-like helicase 1	50.0	422.64	33.19	9	10	10	10	458	6.01	0.002250
O13426	Serine hydroxymethyltransferase, cytosolic	52.0	418.09	27.45	8	14	14	18	470	7.20	0.003947
Q5AME2	Pentafunctional AROM polypeptide	169.3	411.35	7.87	26	10	10	10	1551	6.58	0.000664
Q5AI37	Probable metalloprotease ARX1	62.2	409.80	21.28	1	10	10	10	564	6.92	0.001827
A0A1D8PTW6	Hydroxymethylglutaryl-CoA synthase	49.7	407.62	22.84	6	7	7	8	451	5.97	0.001828
P46596	Opaque-phase-specific protein OP4	41.3	405.27	9.70	1	2	2	3	402	5.26	0.000769
Q5A860	Translationally-controlled tumor protein homolog	18.5	402.59	42.51	2	4	4	5	167	4.46	0.003086
A0A1D8PH78	Farnesyl pyrophosphate synthase	40.7	400.62	17.95	1	7	7	9	351	4.98	0.002642
C4YG73	Vacuolar protein sorting/targeting protein 10	179.2	399.42	9.14	5	7	12	12	1586	5.03	0.000780
O13289	Peroxisomal catalase	54.8	398.35	23.51	2	10	10	11	485	6.65	0.002337
P31353	Phosphomannomutase	29.0	397.33	41.27	3	10	10	12	252	5.69	0.004907
Q5AI15	Polyadenylate-binding protein, cytoplasmic and nuclear	70.4	396.72	22.89	9	12	12	16	629	5.29	0.002621
Q59MA9	Clustered mitochondria protein homolog	155.7	393.17	11.08	2	13	13	13	1363	5.39	0.000983
P83783	Adenosylhomocysteinase	49.0	392.26	29.33	4	12	12	14	450	5.59	0.003206
P43057	Protein kinase C-like 1	125.2	384.61	10.48	1	8	8	8	1097	7.42	0.000752
Q5A4M8	Protein SUR7	29.9	382.09	32.22	1	5	5	7	270	7.69	0.002672
P79023	Phospho-2-dehydro-3-deoxyheptonate aldolase, tyrosine-inhibited	40.3	376.93	30.54	3	9	10	11	370	6.65	0.003064
Q59ZX6	U3 small nucleolar RNA-associated protein 10	204.2	369.22	5.50	5	8	8	9	1818	5.87	0.000510
Q5AF03	Glyoxalase 3	25.8	362.52	42.37	1	6	6	9	236	4.83	0.003930
Q59Y31	Yeast-form wall Protein 1	54.2	358.42	4.50	1	2	2	23	533	4.81	0.004447
O94017	40S ribosomal protein S16	15.7	351.15	45.07	7	6	6	12	142	10.29	0.008709
Q59M70	NADH-cytochrome b5 reductase 2	33.4	348.03	31.89	2	7	7	8	301	8.48	0.002739
O59931	60S ribosomal protein L13	23.0	338.74	45.05	1	7	7	13	202	10.61	0.006632
P0CH96	Adenylosuccinate synthetase	47.9	338.64	26.64	13	10	10	11	428	7.17	0.002649
P47834	60S ribosomal protein L36	11.1	337.73	30.30	2	4	4	7	99	11.40	0.007287
Q5APF2	GMP synthase [glutamine-hydrolyzing]	58.8	336.53	25.66	9	11	11	12	530	5.92	0.002333
Q96UX5	Heat shock protein 78, mitochondrial	91.6	336.09	13.30	2	7	7	8	812	6.80	0.001015
Q92206	Squalene monooxygenase	55.3	335.01	23.99	1	10	10	11	496	8.73	0.002286
Q5AAU3	Protein transport protein SEC31	136.2	331.61	9.25	1	7	7	8	1265	6.77	0.000652

P87066	Tubulin alpha chain	49.9	329.38	18.30	15	7	7	9	448	5.06	0.002070
Q59R28	Alpha-1,2-mannosyltransferase MNN26	87.2	327.63	16.27	1	11	11	11	756	6.52	0.001499
Q5ALX3	Transcription elongation factor SPT5	105.8	321.23	9.83	1	7	7	7	956	5.16	0.000755
Q5ACZ2	Mannan endo-1,6-alpha-mannosidase DFG5	50.0	314.00	14.86	1	5	5	5	451	4.65	0.001143
Q59MN0	Vacuolar protein 8	63.4	301.87	11.62	5	5	5	7	585	5.07	0.001233
Q59MQ0	Myosin-5	146.9	301.64	7.22	17	8	8	8	1316	9.35	0.000626
P39826	Cell division control protein 3	47.8	301.60	18.51	3	6	6	7	416	6.64	0.001734
Q5AGZ9	RuvB-like helicase 2	54.5	301.57	20.08	12	7	7	8	498	5.15	0.001656
P34948	Mannose-6-phosphate isomerase	48.8	299.18	26.08	1	9	9	9	441	5.33	0.002103
Q5ALX8	Adenine phosphoribosyltransferase	20.9	298.07	40.96	1	6	6	7	188	5.31	0.003837
P83780	Glucose-6-phosphate isomerase	61.1	294.28	25.09	6	10	10	11	550	6.40	0.002061
Q9P4E9	GTP-binding nuclear protein GSP1/Ran	24.3	293.75	51.87	8	9	9	14	214	7.02	0.006742
Q5AI86	Eukaryotic translation initiation factor 3 subunit I	38.2	290.07	22.00	8	3	7	8	350	5.47	0.002356
O43101	Centromere/microtubule-binding protein CBF5	54.3	287.17	29.65	1	9	9	10	479	9.20	0.002151
Q59XX2	Cell surface mannoprotein MP65	39.2	287.07	17.99	1	4	4	5	378	5.45	0.001363
P0CY31	Ras-related protein SEC4	23.1	285.43	31.90	1	5	5	6	210	5.47	0.002944
Q59WB3	S-adenosylmethionine permease GAP4	66.4	284.53	9.06	1	5	5	6	607	7.46	0.001019
Q59LF3	Regulator of cytoskeleton and endocytosis RVS167	49.2	282.97	21.59	1	6	6	6	440	6.02	0.001405
Q5ACU6	ATP-dependent rRNA helicase RRP3	59.8	280.36	20.97	8	8	8	9	534	9.73	0.001737
Q5AAW3	ATP-dependent RNA helicase DHH1	62.1	278.53	17.12	8	8	8	9	549	8.75	0.001689
Q5ADM9	Dolichyl-phosphate-mannose--protein mannosyltransferase 2	88.3	277.52	8.19	1	6	6	7	769	7.21	0.000938
Q00314	Vanadate resistance protein	50.7	276.95	16.97	2	5	5	6	442	5.41	0.001399
Q5A302	Endoplasmic reticulum vesicle protein 25	24.5	269.27	20.00	1	5	5	8	215	7.12	0.003835
A0A1D8PCL1	High-affinity glucose transporter 1	60.6	266.76	16.51	1	8	8	9	545	7.62	0.001702
Q5ADW3	Cullin-associated NEDD8-dissociated protein 1	134.4	263.55	4.94	1	4	4	5	1195	4.96	0.000431
Q5APT8	ATP-dependent RNA helicase DBP3	63.1	258.98	16.13	16	8	8	8	564	9.45	0.001462
Q5A1D5	FACT complex subunit SPT16	121.3	256.30	9.15	1	8	8	8	1060	5.16	0.000778
Q59S06	Nucleolar protein 58	57.1	253.37	17.25	5	6	6	6	516	8.31	0.001198
Q5A762	Multiple drug resistance-associated protein-like transporter 1	180.6	252.40	6.72	1	9	9	9	1606	6.32	0.000578
G1UB11	C-22 sterol desaturase ERG5	59.6	251.83	23.02	1	9	9	10	517	6.64	0.001993
Q5AK59	ATP-dependent RNA helicase HAS1	63.0	248.83	13.63	29	7	7	7	565	8.91	0.001277
A0A1D8PN12	Glycerophosphodiester transporter GIT2	59.3	247.03	12.36	1	5	5	6	534	8.10	0.001158

Q9P975	Eukaryotic translation initiation factor 4E	24.2	246.47	27.27	1	5	5	7	209	5.26	0.003452
O74189	Dolichyl-phosphate-mannose--protein mannosyltransferase 1	99.9	245.76	9.35	1	6	6	8	877	6.95	0.000940
Q59L13	Eukaryotic translation initiation factor 6	26.4	245.06	22.45	7	4	4	5	245	4.58	0.002103
P30572	Chitin synthase 2	115.5	240.41	8.62	1	6	6	8	1009	5.73	0.000817
A0A1D8PLI2	Isopentenyl-diphosphate delta-isomerase	32.3	239.51	24.65	1	4	4	4	284	4.97	0.001452
Q5ANB2	ATP-dependent RNA helicase DBP10	103.5	239.31	10.02	12	9	9	9	908	9.07	0.001021
Q9Y7F0	Peroxiredoxin TSA1	21.8	236.44	32.14	4	5	5	5	196	5.06	0.002629
P87220	V-type proton ATPase subunit D	30.0	234.20	16.48	1	2	2	3	267	5.85	0.001158
Q59Q46	Inosine-5'-monophosphate dehydrogenase	56.2	233.32	5.76	1	2	2	3	521	6.55	0.000593
Q5ABV6	SWI5-dependent HO expression protein 3	59.2	232.98	13.10	4	5	5	6	519	9.20	0.001191
P43060	Phosphoribosylaminoimidazole-succinocarboxamide synthase	32.9	232.71	27.49	7	7	7	8	291	5.50	0.002833
P43070	Glucan 1,3-beta-glucosidase	33.5	230.36	7.79	2	2	2	7	308	4.78	0.002342
P87023	Beta-glucan synthesis-associated protein KRE6	82.4	225.80	8.65	1	3	4	6	740	4.78	0.000836
Q5A4X0	E3 ubiquitin-protein ligase BRE1	78.5	220.52	6.75	1	4	4	4	681	6.14	0.000605
Q5AFA2	Extracellular glycosidase CRH11	46.7	219.96	18.98	1	7	7	8	453	4.96	0.001820
Q59L72	GPI-anchored protein 52	41.4	216.32	14.58	1	4	4	5	384	4.91	0.001342
P0CY33	Cell division control protein 42 homolog	21.2	214.79	18.85	1	3	3	6	191	6.54	0.003237
Q5ANE3	Non-classical export protein 102	18.1	214.05	20.59	1	2	2	4	170	9.01	0.002425
Q59XQ1	3'(2'),5'-bisphosphate nucleotidase 2	38.7	213.81	14.80	3	4	4	5	358	5.86	0.001439
Q59NX9	Diphthine methyl ester synthase 1	33.9	210.89	20.74	4	5	5	6	299	4.87	0.002068
Q5A5S7	Autophagy-related protein 27	28.3	209.97	23.02	1	4	4	4	252	5.53	0.001636
A0A1D8PI71	Squalene synthase ERG9	51.2	208.26	18.75	4	9	9	9	448	6.84	0.002070
P30418	Glycylpeptide N-tetradecanoyltransferase	51.8	201.72	10.42	1	3	3	3	451	6.48	0.000686
O13425	Serine hydroxymethyltransferase, mitochondrial	54.5	201.22	16.43	2	6	6	6	493	8.97	0.001254
Q59VP7	Ribosome biogenesis protein ERB1	97.3	200.65	7.66	13	4	6	6	849	4.74	0.000728
Q9UW25	Oxysterol-binding protein-like protein OBPa	49.5	198.88	22.17	4	8	8	9	433	6.13	0.002142
Q5AFE4	Regulator of cytoskeleton and endocytosis RVS161	30.1	195.81	23.11	1	5	5	5	264	7.14	0.001952
Q59X38	Pescadillo homolog	67.8	194.60	14.65	5	7	7	8	587	6.19	0.001405
Q59WF4	Alpha-1,2-mannosyltransferase MNN2	69.1	193.67	13.23	1	6	6	6	597	6.27	0.001036
P47831	60S ribosomal protein L28 (Fragment)	6.6	189.72	46.77	1	3	3	6	62	9.09	0.009973
P22011	Peptidyl-prolyl cis-trans isomerase	17.6	185.20	27.16	2	3	4	5	162	7.97	0.003181
Q5A455	Protein transport protein SEC23	85.6	185.03	7.35	5	5	5	5	762	5.71	0.000676

Q9P8P7	Ribosomal RNA small subunit methyltransferase NEP1	29.5	184.25	21.35	1	4	4	4	267	8.43	0.001544
P10977	Vacuolar aspartic protease	45.4	183.65	13.13	1	3	3	3	419	4.83	0.000738
P46250	SEC14 cytosolic factor	34.7	180.89	24.58	5	6	6	6	301	6.40	0.002054
Q59ZH9	ATP-dependent RNA helicase MAK5	88.0	177.73	8.44	9	6	6	6	782	6.04	0.000791
Q59ZV5	Eukaryotic translation initiation factor 3 subunit G	30.7	175.57	24.73	2	4	4	4	279	9.06	0.001478
Q9P8Q7	Isocitrate lyase	61.4	174.44	11.27	2	5	5	5	550	7.11	0.000937
C4YLH0	MICOS complex subunit MIC60	62.6	173.52	7.08	3	4	4	4	565	6.44	0.000730
O94072	V-type proton ATPase subunit E	25.4	171.69	28.32	2	5	5	5	226	5.40	0.002280
P46585	Ribose-phosphate pyrophosphokinase 1	35.3	171.66	10.59	1	3	3	4	321	6.79	0.001284
P78590	Elongation factor 1-beta	23.5	170.16	23.47	1	3	3	3	213	4.40	0.001452
O93852	D-arabinono-1,4-lactone oxidase	63.4	167.53	15.98	1	6	6	6	557	6.61	0.001110
Q9HEW1	cAMP-dependent protein kinase regulatory subunit	50.3	165.41	8.28	1	3	3	3	459	5.48	0.000674
Q5AQ76	Protein transport protein SEC24	102.0	164.83	7.87	2	5	5	5	928	5.40	0.000555
Q5AC48	Actin-related protein 4	52.6	164.43	12.18	2	4	4	4	468	5.57	0.000881
P46592	Glycolipid 2-alpha-mannosyltransferase 2	54.5	163.23	12.80	1	4	6	6	461	6.67	0.001341
Q5AAI8	Nucleosome assembly protein 1	49.5	162.60	13.79	1	5	5	5	435	4.31	0.001185
Q5AL27	Palmitoyltransferase AKR1	91.7	162.53	3.44	1	2	2	2	813	6.24	0.000254
Q59YF0	Protein transport protein SSO2	34.3	162.31	14.58	1	4	4	5	295	5.36	0.001747
Q5AJC0	Extracellular glycosidase UTR2	51.7	160.23	9.15	1	4	4	4	470	4.73	0.000877
P83773	Acetyl-CoA hydrolase	58.0	159.65	16.22	2	7	7	7	524	6.92	0.001377
Q9B8D8	Cytochrome c oxidase subunit 2	29.8	158.78	17.94	12	3	3	4	262	4.68	0.001573
P82612	Phosphoglycerate mutase	27.4	158.70	16.53	1	3	3	4	248	6.16	0.001662
Q5AI21	Translocation protein SEC62	33.3	157.47	14.68	2	4	4	4	293	9.51	0.001407
P87219	Sorbose reductase SOU1	30.0	157.39	13.17	1	2	2	3	281	5.30	0.001100
P40954	Chitinase 3	60.0	156.17	4.23	1	1	1	2	567	4.91	0.000364
P43065	Saccharopine dehydrogenase [NAD(+), L-lysine-forming]	42.4	156.05	18.32	1	6	6	7	382	5.44	0.001888
Q5ACK7	ATP-dependent RNA helicase DRS1	69.3	153.47	8.97	2	4	4	4	613	6.19	0.000672
Q59KI4	Chromatin-remodeling ATPase INO80	158.7	150.94	4.18	1	4	4	4	1387	8.09	0.000297
O74712	Histidine biosynthesis trifunctional protein	91.8	150.71	7.16	4	5	5	5	838	5.38	0.000615
Q5ADT9	37S ribosomal protein S10, mitochondrial	27.4	150.17	18.80	1	3	3	3	234	9.48	0.001321
Q5A4Q1	Adenylate kinase	27.6	148.42	21.69	16	5	5	5	249	8.10	0.002069
P43063	Cyclin-dependent kinase 1	36.6	146.42	17.98	16	5	5	5	317	6.61	0.001625

Q5AP66	Phosphatidylinositol transfer protein SFH5	36.6	144.56	14.69	1	3	3	3	320	5.33	0.000966
P34725	Phospho-2-dehydro-3-deoxyheptonate aldolase, phenylalanine-inhibited	40.7	144.00	17.12	1	4	5	5	368	7.11	0.001400
Q59KG2	Respiratory growth induced protein 1	23.6	142.04	11.94	4	2	2	3	201	5.82	0.001538
Q5AAR0	Transcription factor IWS1	45.8	140.46	14.75	1	5	5	5	400	8.12	0.001288
Q5ACI8	Peptidyl-prolyl cis-trans isomerase D	40.7	139.25	11.92	2	3	4	4	369	6.39	0.001117
C4YDC4	High osmolarity signaling protein SHO1	40.8	139.13	10.39	6	3	3	3	385	6.04	0.000803
P87024	Beta-glucan synthesis-associated protein SKN1	83.7	138.94	7.33	1	2	3	3	737	5.15	0.000419
P13649	Orotidine 5'-phosphate decarboxylase	29.9	137.79	11.48	3	2	2	2	270	5.76	0.000763
G1UB61	Septin CDC11	46.7	136.13	15.67	1	5	5	5	402	5.07	0.001282
Q59SU1	Candidapepsin-9	58.4	133.16	9.38	2	5	5	5	544	5.25	0.000947
Q5A4P9	ATP-dependent RNA helicase DBP9	65.1	132.63	7.67	4	4	4	4	574	9.06	0.000718
O74933	UDP-N-acetylglucosamine pyrophosphorylase	54.6	128.90	10.49	1	4	4	4	486	6.28	0.000848
A0A1D8PC43	Diphosphomevalonate decarboxylase	39.5	127.96	11.88	1	4	4	4	362	6.46	0.001139
Q59KF3	AdoMet-dependent rRNA methyltransferase SPB1	96.8	127.05	3.79	1	3	3	3	845	5.76	0.000366
Q5AFT3	Protein CFT1	161.8	125.82	3.03	1	2	2	3	1420	5.21	0.000218
Q5AED9	Branchpoint-bridging protein	50.0	125.76	5.93	1	2	2	3	455	9.42	0.000679
C4YS65	Protein SEY1	90.2	125.71	5.95	5	3	3	3	790	5.15	0.000391
O94150	37S ribosomal protein S9, mitochondrial	38.6	125.47	9.52	1	3	3	3	336	10.26	0.000920
C4YJI1	Altered inheritance of mitochondria protein 36, mitochondrial	34.7	123.26	11.64	3	3	3	4	292	8.34	0.001412
Q59W33	Glycerol-3-phosphate dehydrogenase [NAD(+)] 2	40.8	122.47	12.40	1	4	4	4	371	5.31	0.001111
Q5AD78	Mannan endo-1,6-alpha-mannosidase DCW1	50.3	122.25	9.51	1	3	3	3	452	4.73	0.000684
O42617	Poly(A) polymerase PAPalpha	63.2	118.98	5.38	3	2	2	2	558	8.07	0.000369
Q874I4	Dihydroorotate dehydrogenase (quinone), mitochondrial	48.4	118.67	11.94	3	4	4	4	444	9.20	0.000928
P22274	ADP-ribosylation factor	20.2	117.49	27.93	1	1	3	3	179	5.40	0.001727
Q5ABP8	Protein ROT1	29.9	116.08	12.31	2	3	3	4	260	7.80	0.001585
Q5ANP2	Nascent polypeptide-associated complex subunit alpha	19.5	115.77	21.35	3	3	3	3	178	4.82	0.001737
C4YPS3	Regulator of rDNA transcription 14	24.9	114.73	14.61	3	2	2	2	219	9.64	0.000941
Q5AK62	Virulence protein SSD1	141.2	114.00	2.20	1	3	3	4	1274	7.36	0.000324
Q5APF0	Ribosome biogenesis protein YTM1	51.7	113.47	10.30	1	3	3	4	466	6.00	0.000885
Q59X23	Dolichyl-phosphate-mannose--protein mannosyltransferase 4	86.6	112.77	2.78	1	2	2	2	755	8.87	0.000273
P0CU36	Ribosome biogenesis protein C3_06160C_A	29.6	112.27	12.26	7	3	3	4	261	10.18	0.001579
Q9P8E3	Protein transport protein SEC61 subunit alpha	52.5	111.85	8.14	2	3	3	5	479	8.95	0.001076

Q59VX8	Septation protein 7	75.7	111.37	4.78	1	3	3	4	670	6.02	0.000615
O74226	Cell wall synthesis protein KRE9	29.1	111.17	10.33	1	2	2	2	271	8.18	0.000761
Q59NP1	Copper transport protein CTR1	27.8	110.88	12.35	1	2	2	3	251	6.93	0.001232
Q9P844	40S ribosomal protein S21	9.6	109.37	28.74	2	2	2	2	87	8.15	0.002369
Q5AM84	U1 small nuclear ribonucleoprotein component SNU71	70.5	106.73	3.42	1	1	1	1	614	5.20	0.000168
Q5A2J7	Calcium channel YVC1	77.3	105.36	5.93	1	2	2	2	675	5.08	0.000305
Q59MV9	Flavohemoprotein	45.8	104.51	13.32	1	3	3	4	398	5.83	0.001036
Q59VR3	FK506-binding protein 3	47.6	104.22	11.27	1	4	4	4	426	4.46	0.000968
P23286	Calmodulin	16.5	103.67	14.77	1	1	1	1	149	4.41	0.000692
Q59WH0	Transcriptional adapter 2	51.1	102.68	5.39	1	2	2	2	445	7.46	0.000463
Q9HFQ6	60S acidic ribosomal protein P1-B	10.7	102.61	14.81	1	1	1	1	108	3.93	0.000954
Q59TU0	Nascent polypeptide-associated complex subunit beta	17.0	99.69	20.38	1	2	2	2	157	5.71	0.001313
Q59X67	Enhanced filamentous growth protein 1	59.6	98.12	6.18	4	2	2	2	550	9.36	0.000375
Q59P03	NADH-cytochrome b5 reductase 1	32.5	97.86	13.95	1	4	4	5	294	8.10	0.001753
Q59KM8	Cell cycle protein kinase DBF2	82.3	95.53	4.79	1	3	3	3	710	9.13	0.000435
Q9HGY5	Negative regulator of the PHO system	37.3	95.44	11.35	2	3	3	3	326	6.86	0.000948
O42816	Signal recognition particle 54 kDa protein homolog	60.7	95.17	6.29	2	3	3	3	556	9.13	0.000556
Q5AEF2	Protein transport protein SEC13	33.0	94.92	8.05	1	2	2	2	298	5.62	0.000692
Q5ACM9	Eukaryotic translation initiation factor 3 subunit J	32.0	91.50	9.47	1	2	2	2	285	4.98	0.000723
Q59WG0	Adenosine 5'-monophosphoramidase HNT1	17.0	90.66	23.68	1	2	2	2	152	6.86	0.001356
Q5ABD9	Vacuolar protein sorting-associated protein 27	94.9	90.60	2.14	1	1	1	1	841	5.12	0.000123
A0A1D8PNZ7	Glycerophosphocholine phosphodiesterase GDE1	130.8	90.55	2.41	1	2	2	2	1162	6.30	0.000177
Q5AG77	Amino-acid permease GAP1	63.9	89.74	2.75	1	1	1	1	582	9.17	0.000177
P43068	Mitogen-activated protein kinase MKC1	58.2	88.86	6.19	2	2	2	2	501	5.02	0.000411
P46586	ATP phosphoribosyltransferase	32.6	85.89	5.03	1	1	1	1	298	5.34	0.000346
Q92210	Phosphoribosylaminoimidazole carboxylase	62.4	85.63	4.75	2	3	3	3	568	6.49	0.000544
Q59KJ7	Alpha-1,2-mannosyltransferase MNN21	76.9	83.02	2.12	1	1	1	1	660	7.11	0.000156
Q5AKU5	Secreted beta-glucosidase SIM1	39.4	81.17	5.38	1	1	1	1	372	4.87	0.000277
Q5A9Z6	ATP-dependent RNA helicase FAL1	45.5	80.55	3.26	1	1	1	1	399	8.02	0.000258
Q5ACM4	Pre-rRNA-processing protein PNO1	30.6	79.73	6.88	3	2	2	3	276	8.90	0.001120
C4YHS3	RNA polymerase II degradation factor 1	54.1	78.79	3.46	2	1	1	1	492	4.97	0.000209
C4YH95	tRNA (guanine(37)-N1)-methyltransferase	51.8	76.59	6.68	2	2	2	2	449	8.02	0.000459

Q5AJX2	Sphingolipid delta(4)-desaturase	43.4	76.42	4.86	1	1	1	1	370	8.44	0.000279
O13427	Low-specificity L-threonine aldolase	41.8	75.76	4.01	1	1	1	1	374	6.42	0.000276
Q59W44	Mitochondrial import inner membrane translocase subunit TIM50	54.2	75.48	5.12	1	2	2	2	469	6.65	0.000439
Q5ALV2	Actin cytoskeleton-regulatory complex protein SLA1	138.8	73.79	1.83	1	2	2	3	1257	6.05	0.000246
Q9UWF6	Lysophospholipase 1	66.4	73.01	2.15	1	1	1	1	605	4.87	0.000170
Q59MF9	Conserved oligomeric Golgi complex subunit 6	88.4	72.80	5.97	1	3	3	3	771	5.03	0.000401
P87078	DNA topoisomerase 2	165.3	72.41	2.81	1	3	3	3	1461	6.40	0.000212
Q59XM1	Exocyst complex component EXO84	88.5	72.37	1.90	1	1	1	1	791	8.98	0.000130
Q12572	L-2-aminoadipate reductase large subunit	154.6	72.29	1.80	1	1	1	1	1391	6.87	0.000074
Q9HFQ7	60S acidic ribosomal protein P1-A	11.0	72.00	20.75	1	2	2	2	106	4.02	0.001944
A0A1D8PEL1	Mevalonate kinase	47.0	71.19	5.80	1	2	2	2	431	5.68	0.000478
Q5A940	Multiprotein-bridging factor 1	16.3	70.01	7.28	1	1	1	1	151	10.07	0.000682
Q5A310	ISWI chromatin-remodeling complex ATPase ISW2	123.0	69.35	3.03	1	3	3	3	1056	6.73	0.000293
Q5AF95	ATP-dependent RNA helicase DBP4	86.7	69.33	3.27	1	2	2	2	765	8.15	0.000269
Q5A246	DNA-directed RNA polymerase III subunit RPC3	68.3	68.90	5.04	1	2	2	2	595	8.85	0.000346
P31225	Corticosteroid-binding protein	55.5	68.89	3.89	1	2	2	2	489	5.29	0.000421
Q92209	Homoserine kinase	39.1	68.21	7.56	1	2	2	2	357	5.67	0.000577
Q59PR3	ATP-dependent RNA helicase DBP8	48.8	67.36	4.32	13	1	2	2	440	8.91	0.000468
P0CB54	ATPase GET3	39.6	66.90	9.14	43	3	3	3	350	5.02	0.000883
Q5A1Q5	Probable kinetochore protein NUF2	56.6	66.42	4.74	1	2	2	2	485	5.55	0.000425
Q5AEM8	Presequence translocated-associated motor subunit PAM17, mitochondrial	21.0	64.76	10.81	1	1	1	1	185	10.14	0.000557
A0A1D8PL26	2-(3-amino-3-carboxypropyl)histidine synthase subunit 2-2	59.1	64.49	2.65	2	1	1	1	529	5.73	0.000195
Q5A1L6	Major glycerophosphoinositol permease GIT3	59.4	64.34	7.10	1	2	2	2	535	8.06	0.000385
O93831	Rab proteins geranylgeranyltransferase component A	72.2	64.22	4.22	1	3	3	3	640	4.81	0.000483
P53697	Mannan polymerase complex subunit MNN9	42.8	63.56	2.72	1	1	1	2	368	9.14	0.000560
Q59S72	GDP-Man:Man(3)GlcNAc(2)-PP-Dol alpha-1,2-mannosyltransferase	70.9	63.46	2.30	1	1	1	1	609	8.53	0.000169
Q5AHG6	Serine/threonine-protein kinase SCH9	88.7	63.16	2.80	1	2	2	2	787	6.20	0.000262
Q00312	Transcription factor RBF1	59.4	61.93	1.71	2	1	1	2	527	8.66	0.000391
Q9UVL1	Non-histone chromosomal protein 6	10.5	61.76	28.26	2	2	2	2	92	9.70	0.002240
Q59ST8	mRNA cleavage and polyadenylation factor CLP1	54.7	60.10	3.48	1	1	1	1	489	5.35	0.000211
Q5ANL6	13 kDa ribonucleoprotein-associated protein	13.6	60.08	26.98	4	2	2	2	126	7.97	0.001636
Q5AJS6	Multiple RNA-binding domain-containing protein 1	94.9	59.66	1.55	1	1	1	1	841	5.62	0.000123

Q5A216	Probable kinetochore protein NDC80	92.4	58.95	1.78	1	1	1	1	788	5.45	0.000131
Q5A4W8	Bromodomain-containing factor 1	82.5	58.85	3.55	1	2	2	2	732	5.02	0.000282
Q5AAJ7	SWR1-complex protein 4	74.0	58.74	1.42	1	1	1	1	635	8.19	0.000162
Q5A1B0	Sterol-4-alpha-carboxylate 3-dehydrogenase ERG26, decarboxylating	39.2	58.44	6.57	2	3	3	3	350	6.73	0.000883
Q5A119	Chromatin modification-related protein EAF1	79.5	58.44	1.90	1	1	1	1	686	9.76	0.000150
P84149	mRNA export factor MEX67	68.2	58.19	5.19	1	2	2	2	617	8.82	0.000334
Q8J0Q0	Mannosyl-oligosaccharide 1,2-alpha-mannosidase	64.6	58.15	4.78	1	3	3	3	565	5.11	0.000547
Q5ABG1	Histone-lysine N-methyltransferase, H3 lysine-4 specific	119.1	57.88	1.44	1	1	1	1	1040	7.75	0.000099
A0A1D8PCB9	C-8 sterol isomerase ERG2	24.5	57.62	5.53	1	1	1	2	217	5.48	0.000950
Q5AL52	Formin BNI1	196.7	56.59	0.81	1	1	1	1	1732	6.15	0.000060
Q5A3P6	Serine/threonine-protein kinase PKH2	106.5	56.21	1.80	1	1	1	1	947	8.28	0.000109
Q5APM7	ATP-dependent RNA helicase MSS116, mitochondrial	76.7	55.89	2.69	4	2	2	2	668	9.48	0.000309
P53705	Bud site selection protein BUD4	192.8	55.80	0.64	1	1	1	1	1709	6.04	0.000060
Q5A2A2	Mitochondrial homologous recombination protein 1	28.3	54.48	4.60	1	1	1	1	239	8.50	0.000431
O13359	Kexin	105.1	54.38	1.60	1	1	1	1	938	5.03	0.000110
Q9HFQ4	60S acidic ribosomal protein P2-B	11.2	53.82	10.81	1	1	1	1	111	4.07	0.000928
Q5ALL8	FACT complex subunit POB3	60.9	53.79	5.20	1	2	2	2	538	4.83	0.000383
Q5AP65	Protein FMP52, mitochondrial	24.3	53.07	8.30	1	2	2	2	229	8.98	0.000900
Q59VP2	Histone H2A.2	13.8	52.83	25.19	15	1	2	2	131	10.24	0.001573
Q5AI97	Mitochondrial genome maintenance protein MGM101	30.7	52.32	5.45	2	1	1	1	275	9.10	0.000375
P39827	Cell division control protein 10	40.7	51.93	5.32	1	1	1	1	357	7.11	0.000289
Q96WL3	Protein URE2	39.1	51.70	3.49	1	1	1	1	344	5.94	0.000300
O14415	Signal recognition particle SEC65 subunit	31.1	51.59	6.91	1	2	2	2	275	7.53	0.000750
Q59Y41	DNA mismatch repair protein MSH3	118.8	51.56	0.96	1	1	1	1	1037	7.52	0.000099
Q59S59	ATP-dependent RNA helicase MRH4, mitochondrial	62.5	51.45	2.88	1	1	1	1	555	9.70	0.000186
Q5AHJ5	Transcription factor TBF1	100.8	51.45	1.13	1	1	1	1	886	4.67	0.000116
Q5AHB1	Actin cytoskeleton-regulatory complex protein PAN1	152.3	51.34	1.22	1	1	1	1	1396	6.23	0.000074
A0A1D8PJ25	Delta(24(24(1)))-sterol reductase	54.8	51.19	4.26	1	2	2	2	469	7.06	0.000439
P52499	Protein RCC1	51.3	51.06	4.74	1	2	2	2	464	5.43	0.000444
P46588	DNA polymerase delta catalytic subunit	118.8	50.91	0.96	1	1	1	1	1038	8.21	0.000099
P0C8K9	Cytochrome c oxidase subunit 1	58.7	50.75	3.01	1	1	1	1	531	6.37	0.000194
Q5A368	Lysophospholipase NTE1	155.6	49.96	0.87	1	1	1	1	1386	8.38	0.000074

Q5AD77	Sorting nexin-4	71.8	49.93	2.06	1	1	1	1	630	5.63	0.000164
Q5A744	Protein SDS23	67.5	49.31	4.94	1	2	2	2	628	8.44	0.000328
Q9P836	60S ribosomal protein L37 (Fragment)	9.9	48.94	18.18	1	2	2	2	88	11.63	0.002342
Q92207	Mitogen-activated protein kinase HOG1	42.9	48.87	4.24	3	2	2	2	377	5.19	0.000547
O93875	Delta(7)-sterol 5(6)-desaturase	45.4	48.86	4.15	3	2	2	2	386	6.80	0.000534
Q5AQ57	37S ribosomal protein S25, mitochondrial	34.2	48.50	4.41	1	1	1	1	295	8.03	0.000349
O93803	mRNA-capping enzyme subunit beta	58.8	47.79	2.12	1	1	1	1	520	7.87	0.000198
Q5AA50	Nuclear protein localization protein 4	67.3	47.49	2.34	1	1	1	1	598	6.29	0.000172
Q5ABT8	Beta-mannosyltransferase 4	90.9	46.63	2.18	1	1	1	1	781	5.06	0.000132
Q5AKU3	CAP1-binding-protein	73.9	45.05	1.87	1	1	1	1	643	5.92	0.000160
Q5ALL3	tRNA-dihydrouridine(47) synthase [NAD(P)(+)]	69.6	45.00	2.28	1	1	1	1	613	7.69	0.000168
Q59LF9	Methionine aminopeptidase 2	50.3	44.89	2.47	2	1	1	2	446	5.54	0.000462
Q5APQ8	Putative alpha-1,3-mannosyltransferase MNN12	97.5	44.87	1.57	1	1	1	1	828	8.16	0.000124
Q5AIA1	Glucan 1,3-beta-glucosidase 2	54.5	43.67	5.85	1	2	2	2	479	5.53	0.000430
Q5A599	Histidine protein kinase NIK1	118.9	43.50	1.30	1	1	1	1	1081	5.80	0.000095
Q5A888	3-keto-steroid reductase ERG27	39.0	42.87	3.18	2	1	1	1	346	7.91	0.000298
P53698	Cytochrome c	12.2	42.79	19.09	4	2	2	3	110	9.66	0.002811
Q5AD51	Ergosterol biosynthetic protein 28	16.0	42.78	7.97	1	1	1	1	138	9.31	0.000747
Q5APC0	Golgi apparatus membrane protein TVP18	18.8	42.59	13.87	1	1	1	1	173	7.74	0.000596
Q59N80	Inosine triphosphate pyrophosphatase	22.0	42.42	4.46	1	1	1	1	202	5.66	0.000510
Q5A309	Histone-lysine N-methyltransferase, H3 lysine-79 specific	155.6	42.32	0.89	1	1	1	1	1343	6.38	0.000077
A0A1D8PN88	Amino-acid permease GAP3	65.5	42.20	1.84	1	1	1	1	599	8.43	0.000172
Q9HF78	Glutamate--cysteine ligase	79.1	41.74	1.59	1	1	1	1	690	5.34	0.000149
Q5A360	Nucleotide exchange factor SIL1	47.0	41.12	3.62	1	1	1	1	414	5.07	0.000249
P52498	Ras-related protein RSR1	27.6	41.10	7.66	2	2	2	2	248	5.21	0.000831
C4YTG0	Pheromone-processing carboxypeptidase KEX1	78.7	40.50	1.28	2	1	1	1	702	5.12	0.000147
P78600	Proline--tRNA ligase, cytoplasmic	66.2	40.16	2.43	1	1	1	1	575	5.96	0.000179
Q9UVX1	Lysophospholipase 3	81.4	39.68	1.06	1	1	1	1	754	4.84	0.000137
C4YS59	Vacuolar membrane protease	93.7	38.76	1.79	2	1	1	2	837	5.29	0.000246
Q59RP7	54S ribosomal protein L4, mitochondrial	36.3	38.32	2.59	1	1	1	1	309	5.96	0.000334
Q5A0E2	Exportin-T	118.4	37.81	1.76	1	1	1	1	1025	5.06	0.000101
C4YKP5	Increased recombination centers protein 22-1	25.9	36.73	10.46	4	2	2	2	239	5.68	0.000862

Q59RH5	Histone acetyltransferase type B subunit 2	42.8	35.95	2.36	1	1	1	1	382	5.05	0.000270
Q5AP53	Serine/threonine-protein kinase CBK1	84.4	35.88	3.01	1	2	2	2	732	6.40	0.000282
A0A1D8PH52	Acetyl-CoA acetyltransferase	41.9	33.83	4.73	1	2	2	2	402	6.90	0.000513
Q5A3V6	3,4-dihydroxy-2-butanone 4-phosphate synthase	22.6	33.82	3.43	1	1	1	1	204	5.44	0.000505
Q5AD56	General negative regulator of transcription subunit 3	85.7	33.58	5.19	1	2	2	2	752	5.57	0.000274
P43066	D-arabinitol 2-dehydrogenase [ribulose-forming]	30.6	33.56	2.85	2	1	1	1	281	5.86	0.000367
P87163	Translation initiation factor eIF-2B subunit epsilon	81.9	33.22	1.64	1	1	1	1	732	4.65	0.000141
Q5AAT0	Decapping nuclease RAI1	45.7	32.73	2.30	1	1	1	1	391	8.10	0.000264
Q59V93	Very-long-chain 3-oxoacyl-CoA reductase	38.3	32.20	2.29	1	1	1	1	349	9.57	0.000295
O14427	Serine/threonine-protein kinase CLA4	106.8	31.25	2.88	2	3	3	3	971	9.35	0.000318
Q5ADQ9	Beta-mannosyltransferase 1	79.6	30.94	1.46	1	1	1	1	684	7.88	0.000151
Q59YL9	Pre-rRNA-processing protein ESF2	36.8	30.19	3.75	1	1	1	1	320	8.40	0.000322
Q5ACL9	Protein BFR2	58.4	30.08	1.95	1	1	1	1	512	4.74	0.000201
Q5AM44	mRNA 3'-end-processing protein RNA14	91.6	29.81	1.26	1	1	1	1	791	5.21	0.000130
A0A1D8PNP3	Amino-acid permease GAP6	61.8	29.45	1.58	1	1	1	1	568	8.72	0.000181
Q5AG40	Vacuolar protein sorting-associated protein 4	48.4	29.37	2.73	1	1	1	1	439	5.64	0.000235
Q5A1M3	Potential protein lysine methyltransferase SET5	53.8	29.19	1.27	2	1	1	1	473	7.30	0.000218
Q5ABD0	Vacuolar-sorting protein SNF7	25.9	28.51	5.31	1	1	1	1	226	4.88	0.000456
Q5A895	Class E vacuolar protein-sorting machinery protein HSE1	56.6	28.29	1.81	1	1	1	1	498	6.76	0.000207
Q59Q38	Peptide-N(4)-(N-acetyl-beta-glucosaminyl)asparagine amidase	45.8	27.68	2.28	1	1	1	1	395	7.18	0.000261
A0A1D8PD39	3-hydroxy-3-methylglutaryl-coenzyme A reductase 1	116.4	27.66	1.49	3	2	2	2	1073	7.33	0.000192
P43073	N-(5'-phosphoribosyl)anthranilate isomerase	24.8	27.29	5.75	1	1	1	1	226	5.33	0.000456
O74254	Glucoamylase 1	105.7	25.69	0.74	2	1	1	1	946	4.92	0.000109
Q5AP90	Alpha-1,2-mannosyltransferase MNN23	69.6	25.66	1.32	1	1	1	1	606	5.40	0.000170
Q59U67	Transcription initiation factor TFIID subunit 4	39.6	25.38	2.75	1	1	1	1	363	9.13	0.000284
Q5A0W6	GPN-loop GTPase 3	30.8	23.79	4.76	1	1	1	1	273	4.50	0.000377
Q59PE7	Protein BCP1	37.4	22.93	3.12	1	1	1	1	321	4.77	0.000321
P30574	Carboxypeptidase Y	61.0	22.91	1.48	1	1	1	1	542	5.47	0.000190
G1UB37	Major facilitator superfamily multidrug transporter FLU1	67.6	22.45	1.64	1	1	1	1	610	6.65	0.000169
Q92410	Alpha,alpha-trehalose-phosphate synthase [UDP-forming]	54.4	19.23	3.35	1	2	2	2	478	6.23	0.000431
Q5AGM0	Protein HIR2	114.4	18.68	1.18	1	1	1	1	1017	8.12	0.000101
Q5A761	CCR4-Not complex 3'-5'-exoribonuclease subunit Ccr4	90.3	17.82	1.52	1	1	1	1	787	7.20	0.000131

Table S2. Shared or unique proteins identified as cargo of *Candida albicans* EVs. The presence of the identified protein is marked with "+" and absence with "-".

Accession number	Description	EV_{CON}	EV_{AMB}	EV_{FLU}	EV_{CASP}
Q96UX5	Heat shock protein 78, mitochondrial	+	+	+	+
P82610	5-methyltetrahydropteroyltriglutamate--homocysteine methyltransferase	+	+	+	+
Q5AKA5	Cys-Gly metallodipeptidase DUG1	+	+	+	+
Q5AME2	Pentafunctional AROM polypeptide	+	+	+	+
P83783	Adenosylhomocysteinase	+	+	+	+
P79023	Phospho-2-dehydro-3-deoxyheptonate aldolase, tyrosine-inhibited	+	+	+	+
P0CH96	Adenylosuccinate synthetase	+	+	+	+
O13425	Serine hydroxymethyltransferase, mitochondrial	+	+	+	+
P43065	Saccharopine dehydrogenase [NAD(+), L-lysine-forming]	+	+	+	+
O74712	Histidine biosynthesis trifunctional protein	+	+	+	+
P34725	Phospho-2-dehydro-3-deoxyheptonate aldolase, phenylalanine-inhibited	+	+	+	+
O13427	Low-specificity L-threonine aldolase	+	+	+	+
Q92211	Glyceraldehyde-3-phosphate dehydrogenase	+	+	+	+
P83779	Pyruvate decarboxylase	+	+	+	+
P43067	Alcohol dehydrogenase 1	+	+	+	+
Q9URB4	Fructose-bisphosphate aldolase	+	+	+	+
P83776	Hexokinase-2	+	+	+	+
P30575	Enolase 1	+	+	+	+
O94039	Transketolase 1	+	+	+	+
P46273	Phosphoglycerate kinase	+	+	+	+
Q8NJN3	Acetyl-coenzyme A synthetase 2	+	+	+	+
P82611	Aconitate hydratase, mitochondrial	+	+	+	+
Q59KI0	UTP--glucose-1-phosphate uridylyltransferase	+	+	+	+
P83778	Malate dehydrogenase, cytoplasmic	+	+	+	+
P30573	Chitin synthase 3	+	+	+	+
O94200	ATP-dependent 6-phosphofructokinase subunit beta	+	+	+	+
Q5AIR7	Endo-1,3(4)-beta-glucanase 1	+	+	+	+
O93827	Mannose-1-phosphate guanyltransferase	+	+	+	+
O94201	ATP-dependent 6-phosphofructokinase subunit alpha	+	+	+	+
P53704	Glutamine--fructose-6-phosphate aminotransferase [isomerizing]	+	+	+	+
O13287	6-phosphogluconate dehydrogenase, decarboxylating	+	+	+	+

O94038	Alcohol dehydrogenase 2	+	+	+	+
O13434	Phosphoenolpyruvate carboxykinase [ATP]	+	+	+	+
P29717	Glucan 1,3-beta-glucosidase	+	+	+	+
Q9P940	Triosephosphate isomerase	+	+	+	+
P31353	Phosphomannomutase	+	+	+	+
Q5AF03	Glyoxalase 3	+	+	+	+
Q59M70	NADH-cytochrome b5 reductase 2	+	+	+	+
P34948	Mannose-6-phosphate isomerase	+	+	+	+
P83780	Glucose-6-phosphate isomerase	+	+	+	+
Q00314	Vanadate resistance protein	+	+	+	+
P30572	Chitin synthase 2	+	+	+	+
P43070	Glucan 1,3-beta-glucosidase	+	+	+	+
P87023	Beta-glucan synthesis-associated protein KRE6	+	+	+	+
O93852	D-arabinono-1,4-lactone oxidase	+	+	+	+
P83773	Acetyl-CoA hydrolase	+	+	+	+
P82612	Phosphoglycerate mutase	+	+	+	+
P87219	Sorbose reductase SOU1	+	+	+	+
P40954	Chitinase 3	+	+	+	+
Q59P03	NADH-cytochrome b5 reductase 1	+	+	+	+
Q8J0Q0	Mannosyl-oligosaccharide 1,2-alpha-mannosidase	+	+	+	+
Q92410	Alpha,alpha-trehalose-phosphate synthase [UDP-forming]	+	+	+	+
P53705	Bud site selection protein BUD4	+	+	+	+
Q9Y872	Sulfate adenylyltransferase	+	+	+	+
Q59PT0	V-type proton ATPase subunit B	+	+	+	+
P83782	Cytochrome b-c1 complex subunit 2, mitochondrial	+	+	+	+
P83777	Inorganic pyrophosphatase	+	+	+	+
Q5AJB1	V-type proton ATPase catalytic subunit A	+	+	+	+
P87220	V-type proton ATPase subunit D	+	+	+	+
Q9P8Q7	Isocitrate lyase	+	+	+	+
Q9B8D8	Cytochrome c oxidase subunit 2	+	+	+	+
Q59KG2	Respiratory growth induced protein 1	+	+	+	+
P0C8K9	Cytochrome c oxidase subunit 1	+	+	+	+
P53698	Cytochrome c	+	+	+	+
P46587	Heat shock protein SSA2	+	+	+	+
P41797	Heat shock protein SSA1	+	+	+	+

P87222	Heat shock protein SSB1	+	+	+	+
Q96VB9	Heat shock protein homolog SSE1	+	+	+	+
P83784	Heat shock protein SSC1, mitochondrial	+	+	+	+
P46598	Heat shock protein 90 homolog	+	+	+	+
O74261	Heat shock protein 60, mitochondrial	+	+	+	+
P47828	T-complex protein 1 subunit theta	+	+	+	+
Q59S78	Small COPII coat GTPase SAR1	+	+	+	+
Q5AAU3	Protein transport protein SEC31	+	+	+	+
Q5AAW3	ATP-dependent RNA helicase DHH1	+	+	+	+
P22011	Peptidyl-prolyl cis-trans isomerase	+	+	+	+
Q5A455	Protein transport protein SEC23	+	+	+	+
P10977	Vacuolar aspartic protease	+	+	+	+
Q5AQ76	Protein transport protein SEC24	+	+	+	+
Q59YF0	Protein transport protein SSO2	+	+	+	+
Q5AI21	Translocation protein SEC62	+	+	+	+
Q5ABP8	Protein ROT1	+	+	+	+
Q9P8E3	Protein transport protein SEC61 subunit alpha	+	+	+	+
O14415	Signal recognition particle SEC65 subunit	+	+	+	+
C4YTG0	Pheromone-processing carboxypeptidase KEX1	+	+	+	+
C4YS59	Vacuolar membrane protease	+	+	+	+
Q5AD56	General negative regulator of transcription subunit 3	+	+	+	+
Q5A761	CCR4-Not complex 3'-5'-exoribonuclease subunit Ccr4	+	+	+	+
Q00310	Glycolipid 2-alpha-mannosyltransferase 1	+	+	+	+
Q59XX2	Cell surface mannoprotein MP65	+	+	+	+
Q5ADM9	Dolichyl-phosphate-mannose--protein mannosyltransferase 2	+	+	+	+
O74189	Dolichyl-phosphate-mannose--protein mannosyltransferase 1	+	+	+	+
Q5AFA2	Extracellular glycosidase CRH11	+	+	+	+
Q59WF4	Alpha-1,2-mannosyltransferase MNN2	+	+	+	+
P46592	Glycolipid 2-alpha-mannosyltransferase 2	+	+	+	+
Q5AJC0	Extracellular glycosidase UTR2	+	+	+	+
P87024	Beta-glucan synthesis-associated protein SKN1	+	+	+	+
O74226	Cell wall synthesis protein KRE9	+	+	+	+
Q59KJ7	Alpha-1,2-mannosyltransferase MNN21	+	+	+	+
P53697	Mannan polymerase complex subunit MNN9	+	+	+	+
Q59S72	GDP-Man:Man(3)GlcNAc(2)-PP-Dol alpha-1,2-mannosyltransferase	+	+	+	+

P34731	Fatty acid synthase subunit beta	+	+	+	+
P43098	Fatty acid synthase subunit alpha	+	+	+	+
P10613	Lanosterol 14-alpha demethylase	+	+	+	+
Q5APD4	Sphingolipid C9-methyltransferase	+	+	+	+
Q92206	Squalene monooxygenase	+	+	+	+
G1UB11	C-22 sterol desaturase ERG5	+	+	+	+
A0A1D8PI71	Squalene synthase ERG9	+	+	+	+
Q59W33	Glycerol-3-phosphate dehydrogenase [NAD(+)] 2	+	+	+	+
A0A1D8PNZ7	Glycerophosphocholine phosphodiesterase GDE1	+	+	+	+
Q9UWF6	Lysophospholipase 1	+	+	+	+
Q5A1B0	Sterol-4-alpha-carboxylate 3-dehydrogenase ERG26, decarboxylating	+	+	+	+
A0A1D8PJ25	Delta(24(24(1)))-sterol reductase	+	+	+	+
O93875	Delta(7)-sterol 5(6)-desaturase	+	+	+	+
Q9UVX1	Lysophospholipase 3	+	+	+	+
Q59V93	Very-long-chain 3-oxoacyl-CoA reductase	+	+	+	+
O74198	Sterol 24-C-methyltransferase	+	+	+	+
P31225	Corticosteroid-binding protein	+	+	+	+
Q5A3V6	3,4-dihydroxy-2-butanone 4-phosphate synthase	+	+	+	+
A0A1D8PTW6	Hydroxymethylglutaryl-CoA synthase	+	+	+	+
A0A1D8PH78	Farnesyl pyrophosphate synthase	+	+	+	+
A0A1D8PLI2	Isopentenyl-diphosphate delta-isomerase	+	+	+	+
A0A1D8PC43	Diphosphomevalonate decarboxylase	+	+	+	+
A0A1D8PEL1	Mevalonate kinase	+	+	+	+
A0A1D8PD39	3-hydroxy-3-methylglutaryl-coenzyme A reductase 1	+	+	+	+
Q5APF2	GMP synthase [glutamine-hydrolyzing]	+	+	+	+
Q5ALX8	Adenine phosphoribosyltransferase	+	+	+	+
Q59Q46	Inosine-5'-monophosphate dehydrogenase	+	+	+	+
P43060	Phosphoribosylaminoimidazole-succinocarboxamide synthase	+	+	+	+
P46585	Ribose-phosphate pyrophosphokinase 1	+	+	+	+
Q5A4Q1	Adenylate kinase	+	+	+	+
Q874I4	Dihydroorotate dehydrogenase (quinone), mitochondrial	+	+	+	+
Q92210	Phosphoribosylaminoimidazole carboxylase	+	+	+	+
O42617	Poly(A) polymerase PAPalpha	+	+	+	+
O42816	Signal recognition particle 54 kDa protein homolog	+	+	+	+
P87078	DNA topoisomerase 2	+	+	+	+

P25997	Elongation factor 3	+	+	+	+
P83774	Guanine nucleotide-binding protein subunit beta-like protein	+	+	+	+
P40910	40S ribosomal protein S1	+	+	+	+
P87206	ATP-dependent RNA helicase eIF4A	+	+	+	+
Q5AML1	Eukaryotic translation initiation factor 3 subunit C	+	+	+	+
P52495	Ubiquitin-activating enzyme E1 1	+	+	+	+
O94049	Acetyl-coenzyme A synthetase 1	+	+	+	+
Q5AGV4	Eukaryotic translation initiation factor 3 subunit B	+	+	+	+
Q5A4E2	ATP-dependent RNA helicase DED1	+	+	+	+
O94083	Eukaryotic translation initiation factor 5A	+	+	+	+
Q59PR9	Transcriptional regulator HMO1	+	+	+	+
Q5A0W7	RuvB-like helicase 1	+	+	+	+
Q5AI37	Probable metalloprotease ARX1	+	+	+	+
Q59MA9	Clustered mitochondria protein homolog	+	+	+	+
P43057	Protein kinase C-like 1	+	+	+	+
Q5ALX3	Transcription elongation factor SPT5	+	+	+	+
Q59MN0	Vacuolar protein 8	+	+	+	+
P39826	Cell division control protein 3	+	+	+	+
Q5AGZ9	RuvB-like helicase 2	+	+	+	+
Q5AI86	Eukaryotic translation initiation factor 3 subunit I	+	+	+	+
Q5ACU6	ATP-dependent rRNA helicase RRP3	+	+	+	+
Q5A302	Endoplasmic reticulum vesicle protein 25	+	+	+	+
Q5APT8	ATP-dependent RNA helicase DBP3	+	+	+	+
Q5A1D5	FACT complex subunit SPT16	+	+	+	+
Q5AK59	ATP-dependent RNA helicase HAS1	+	+	+	+
Q9P975	Eukaryotic translation initiation factor 4E	+	+	+	+
Q5ANB2	ATP-dependent RNA helicase DBP10	+	+	+	+
Q5ABV6	SWI5-dependent HO expression protein 3	+	+	+	+
Q5A4X0	E3 ubiquitin-protein ligase BRE1	+	+	+	+
P0CY33	Cell division control protein 42 homolog	+	+	+	+
Q59NX9	Diphthine methyl ester synthase 1	+	+	+	+
Q59VP7	Ribosome biogenesis protein ERB1	+	+	+	+
Q9UW25	Oxysterol-binding protein-like protein OBPa	+	+	+	+
Q5AFE4	Regulator of cytoskeleton and endocytosis RVS161	+	+	+	+
Q59X38	Pescadillo homolog	+	+	+	+

Q9P8P7	Ribosomal RNA small subunit methyltransferase NEP1	+	+	+	+
Q59ZH9	ATP-dependent RNA helicase MAK5	+	+	+	+
Q59ZV5	Eukaryotic translation initiation factor 3 subunit G	+	+	+	+
Q5AC48	Actin-related protein 4	+	+	+	+
Q5AAI8	Nucleosome assembly protein 1	+	+	+	+
Q5AL27	Palmitoyltransferase AKR1	+	+	+	+
Q5ACK7	ATP-dependent RNA helicase DRS1	+	+	+	+
Q59KI4	Chromatin-remodeling ATPase INO80	+	+	+	+
Q5AP66	Phosphatidylinositol transfer protein SFH5	+	+	+	+
Q5AAR0	Transcription factor IWS1	+	+	+	+
Q5ACI8	Peptidyl-prolyl cis-trans isomerase D	+	+	+	+
O74933	UDP-N-acetylglucosamine pyrophosphorylase	+	+	+	+
Q59KF3	AdoMet-dependent rRNA methyltransferase SPB1	+	+	+	+
Q5AED9	Branchpoint-bridging protein	+	+	+	+
O94150	37S ribosomal protein S9, mitochondrial	+	+	+	+
Q5AK62	Virulence protein SSD1	+	+	+	+
P0CU36	Ribosome biogenesis protein C3_06160C_A	+	+	+	+
Q59WH0	Transcriptional adapter 2	+	+	+	+
Q5ACM9	Eukaryotic translation initiation factor 3 subunit J	+	+	+	+
P43068	Mitogen-activated protein kinase MKC1	+	+	+	+
Q59W44	Mitochondrial import inner membrane translocase subunit TIM50	+	+	+	+
Q59XM1	Exocyst complex component EXO84	+	+	+	+
Q5A310	ISWI chromatin-remodeling complex ATPase ISW2	+	+	+	+
Q5AF95	ATP-dependent RNA helicase DBP4	+	+	+	+
Q59PR3	ATP-dependent RNA helicase DBP8	+	+	+	+
Q5A1Q5	Probable kinetochore protein NUF2	+	+	+	+
Q5AEM8	Presequence translocated-associated motor subunit PAM17, mitochondrial	+	+	+	+
Q00312	Transcription factor RBF1	+	+	+	+
Q5AJS6	Multiple RNA-binding domain-containing protein 1	+	+	+	+
Q5ALL8	FACT complex subunit POB3	+	+	+	+
Q59VP2	Histone H2A.2	+	+	+	+
Q5AHJ5	Transcription factor TBF1	+	+	+	+
P52499	Protein RCC1	+	+	+	+
Q5AQ57	37S ribosomal protein S25, mitochondrial	+	+	+	+
O93803	mRNA-capping enzyme subunit beta	+	+	+	+

Q59RP7	54S ribosomal protein L4, mitochondrial	+	+	+	+
P43076	pH-responsive protein 1	+	+	+	+
Q59KZ1	Aminopeptidase 2	+	+	+	+
Q5AJY5	1,3-beta-glucanosyltransferase PGA4	+	+	+	+
O13318	pH-responsive protein 2	+	+	+	+
Q59MQ0	Myosin-5	+	+	+	+
Q5A599	Histidine protein kinase NIK1	+	+	+	+
P43084	Probable NADPH dehydrogenase	+	+	+	+
P10875	Tubulin beta chain	+	+	+	+
P83781	Mitochondrial outer membrane protein porin	+	+	+	+
O42766	14-3-3 protein homolog	+	+	+	+
P53707	37 kDa cell surface protein	+	+	+	+
O13426	Serine hydroxymethyltransferase, cytosolic	+	+	+	+
P46596	Opaque-phase-specific protein OP4	+	+	+	+
Q59Y31	Yeast-form wall Protein 1	+	+	+	+
P87066	Tubulin alpha chain	+	+	+	+
Q5ACZ2	Mannan endo-1,6-alpha-mannosidase DFG5	+	+	+	+
Q59WB3	S-adenosylmethionine permease GAP4	+	+	+	+
Q9Y7F0	Peroxiredoxin TSA1	+	+	+	+
C4YLH0	MICOS complex subunit MIC60	+	+	+	+
Q9HEW1	cAMP-dependent protein kinase regulatory subunit	+	+	+	+
G1UB61	Septin CDC11	+	+	+	+
C4YS65	Protein SEY1	+	+	+	+
C4YJI1	Altered inheritance of mitochondria protein 36, mitochondrial	+	+	+	+
Q5AD78	Mannan endo-1,6-alpha-mannosidase DCW1	+	+	+	+
Q59VX8	Septation protein 7	+	+	+	+
Q59NP1	Copper transport protein CTR1	+	+	+	+
P23286	Calmodulin	+	+	+	+
Q9HGY5	Negative regulator of the PHO system	+	+	+	+
Q5AG77	Amino-acid permease GAP1	+	+	+	+
Q5AL52	Formin BNI1	+	+	+	+
Q5A3P6	Serine/threonine-protein kinase PKH2	+	+	+	+
P39827	Cell division control protein 10	+	+	+	+
Q92207	Mitogen-activated protein kinase HOG1	+	+	+	+
A0A1D8PN88	Amino-acid permease GAP3	+	+	+	+

O14427	Serine/threonine-protein kinase CLA4	+	+	+	+
A0A1D8PNP3	Amino-acid permease GAP6	+	+	+	+
C4YKT4	Ras-like protein 1	+	+	+	+
O42825	GTP-binding protein RHO1	+	+	+	+
P43063	Cyclin-dependent kinase 1	+	+	+	+
Q59KM8	Cell cycle protein kinase DBF2	+	+	+	+
P52498	Ras-related protein RSR1	+	+	+	+
P43071	Multidrug resistance protein CDR1	+	+	+	+
Q5AEN1	Cytochrome c peroxidase, mitochondrial	+	+	+	+
Q5AHH4	Small heat shock protein 21	+	+	+	+
O13289	Peroxisomal catalase	+	+	+	+
Q59LU0	ATP-dependent RNA helicase DBP2	+	+	+	+
Q5A9Z6	ATP-dependent RNA helicase FAL1	+	+	+	+
Q9UVL1	Non-histone chromosomal protein 6	+	+	+	+
P0CY35	Elongation factor 1-alpha 1	+	+	+	+
C4YJQ8	Elongation factor 2	+	+	+	+
Q5A8K2	Alanine--tRNA ligase	+	+	+	+
Q9HGT6	Serine--tRNA ligase, cytoplasmic	+	+	+	+
O42817	40S ribosomal protein S0	+	+	+	+
P47837	40S ribosomal protein S4	+	+	+	+
Q59PL9	Eukaryotic translation initiation factor 3 subunit A	+	+	+	+
Q9P843	60S ribosomal protein L27	+	+	+	+
Q5AJD0	ATP-dependent RNA helicase DBP5	+	+	+	+
Q96W54	40S ribosomal protein S22	+	+	+	+
Q9UVJ4	60S ribosomal protein L10a	+	+	+	+
O94008	60S ribosomal protein L32	+	+	+	+
O13354	Eukaryotic peptide chain release factor GTP-binding subunit	+	+	+	+
O13432	Phenylalanine--tRNA ligase beta subunit	+	+	+	+
Q96W53	40S ribosomal protein S14	+	+	+	+
Q5A860	Translationally-controlled tumor protein homolog	+	+	+	+
Q5AI15	Polyadenylate-binding protein, cytoplasmic and nuclear	+	+	+	+
Q59ZX6	U3 small nucleolar RNA-associated protein 10	+	+	+	+
O94017	40S ribosomal protein S16	+	+	+	+
O59931	60S ribosomal protein L13	+	+	+	+
P47834	60S ribosomal protein L36	+	+	+	+

O43101	Centromere/microtubule-binding protein CBF5	+	+	+	+
Q59S06	Nucleolar protein 58	+	+	+	+
Q59L13	Eukaryotic translation initiation factor 6	+	+	+	+
P47831	60S ribosomal protein L28 (Fragment)	+	+	+	+
P78590	Elongation factor 1-beta	+	+	+	+
Q5ADT9	37S ribosomal protein S10, mitochondrial	+	+	+	+
Q9P844	40S ribosomal protein S21	+	+	+	+
Q9HFQ6	60S acidic ribosomal protein P1-B	+	+	+	+
Q5AEF2	Protein transport protein SEC13	+	+	+	+
C4YH95	tRNA (guanine(37)-N1)-methyltransferase	+	+	+	+
Q9HFQ7	60S acidic ribosomal protein P1-A	+	+	+	+
Q9P836	60S ribosomal protein L37 (Fragment)	+	+	+	+
P28877	Plasma membrane ATPase 1	+	+	+	+
P14235	Actin	+	+	+	+
O74676	ABC transporter CDR4	+	+	+	+
P34732	Vesicular-fusion protein SEC18	+	+	+	+
C4YG73	Vacuolar protein sorting/targeting protein 10	+	+	+	+
Q9P4E9	GTP-binding nuclear protein GSP1/Ran	+	+	+	+
P0CY31	Ras-related protein SEC4	+	+	+	+
Q59LF3	Regulator of cytoskeleton and endocytosis RVS167	+	+	+	+
A0A1D8PCL1	High-affinity glucose transporter 1	+	+	+	+
Q5A762	Multiple drug resistance-associated protein-like transporter 1	+	+	+	+
Q5ANE3	Non-classical export protein 102	+	+	+	+
Q5A5S7	Autophagy-related protein 27	+	+	+	+
P46250	SEC14 cytosolic factor	+	+	+	+
O94072	V-type proton ATPase subunit E	+	+	+	+
P22274	ADP-ribosylation factor	+	+	+	+
Q5ANP2	Nascent polypeptide-associated complex subunit alpha	+	+	+	+
Q59TU0	Nascent polypeptide-associated complex subunit beta	+	+	+	+
O93831	Rab proteins geranylgeranyltransferase component A	+	+	+	+
Q5ABD0	Vacuolar-sorting protein SNF7	+	+	+	+
Q8TGH6	Guanosine-diphosphatase	+	+	+	+
Q5A4M8	Protein SUR7	+	+	+	+
Q59R28	Alpha-1,2-mannosyltransferase MNN26	+	+	+	+
A0A1D8PN12	Glycerophosphodiester transporter GIT2	+	+	+	+

Q59L72	GPI-anchored protein 52	+	+	+	+
P30418	Glycylpeptide N-tetradecanoyltransferase	+	+	+	+
Q5A2J7	Calcium channel YVC1	+	+	+	+
Q59MV9	Flavohemoprotein	+	+	+	+
Q5A1L6	Major glycerophosphoinositol permease GIT3	+	+	+	+
Q5A2A2	Mitochondrial homologous recombination protein 1	+	+	+	+
Q5AP65	Protein FMP52, mitochondrial	+	+	+	+
Q5AI97	Mitochondrial genome maintenance protein MGM101	+	+	+	+
P46588	DNA polymerase delta catalytic subunit	+	+	+	+
Q5A744	Protein SDS23	+	+	+	+
C4YKP5	Increased recombination centers protein 22-1	+	+	+	+
Q5AP53	Serine/threonine-protein kinase CBK1	+	+	+	+
Q59U67	Transcription initiation factor TFIID subunit 4	+	+	+	+
C4YF50	NAD(P)H-hydrate epimerase	+	+	+	+
O13359	Kexin	+	+	+	+
P56091	Galactokinase	+	+	+	-
Q59PD6	Spindle assembly checkpoint component MAD1	+	+	+	-
Q59LF2	Alpha-1,3/1,6-mannosyltransferase ALG2	+	+	+	-
P87185	Cysteine desulfurase, mitochondrial	+	+	+	-
O74270	Origin recognition complex subunit 1	+	+	+	-
Q9Y7C4	ATP-dependent RNA helicase CHR1	+	+	+	-
Q3MNT0	Transcription elongation factor SPT6	+	+	+	-
Q00313	DNA topoisomerase 1	+	+	+	-
P53716	Uncharacterized protein CAWG_04269, mitochondrial	+	+	+	-
Q5ABG1	Histone-lysine N-methyltransferase, H3 lysine-4 specific	+	+	-	+
Q96WL3	Protein URE2	+	+	-	+
Q5A309	Histone-lysine N-methyltransferase, H3 lysine-79 specific	+	+	-	+
Q9P4V2	Phosphoacetylglucosamine mutase	+	+	-	+
Q5AA50	Nuclear protein localization protein 4	+	+	-	+
Q5A360	Nucleotide exchange factor SIL1	+	+	-	+
P13649	Orotidine 5'-phosphate decarboxylase	+	+	-	+
Q5AG40	Vacuolar protein sorting-associated protein 4]	+	+	-	+
Q5A4P9	ATP-dependent RNA helicase DBP9	+	+	-	+
A0A1D8PL26	2-(3-amino-3-carboxypropyl)histidine synthase subunit 2-2	+	+	-	+
Q59S59	ATP-dependent RNA helicase MRH4, mitochondrial	+	+	-	+

Q5AHB1	Actin cytoskeleton-regulatory complex protein PAN1	+	+	-	+
P87163	Translation initiation factor eIF-2B subunit epsilon	+	+	-	+
Q5AGM0	Protein HIR2	+	+	-	+
Q59WG0	Adenosine 5'-monophosphoramidase HNT1	+	+	-	+
Q5AFT3	Protein CFT1	+	+	-	+
P42800	Inositol-3-phosphate synthase	+	+	-	-
P53699	Cell division control protein 4	+	+	-	-
Q5A3M6	GPI ethanolamine phosphate transferase 1	+	+	-	-
Q5AH60	tRNA (guanine-N(7)-)-methyltransferase non-catalytic subunit TRM82	+	+	-	-
Q59U81	Helicase SWR1	+	+	-	-
Q5A8X7	Protein FYV4, mitochondrial	+	+	-	-
Q59Y46	Morphogenetic regulator of filamentous growth protein 1	+	+	-	-
O42779	Candidapepsin-9	+	+	-	-
Q12572	L-2-aminoadipate reductase large subunit	+	-	+	+
Q59X67	Enhanced filamentous growth protein 1	+	-	+	+
Q59X23	Dolichyl-phosphate-mannose--protein mannosyltransferase 4	+	-	+	+
Q5A368	Lysophospholipase NTE1	+	-	+	+
Q5ADW3	Cullin-associated NEDD8-dissociated protein 1	+	-	+	+
Q5APM7	ATP-dependent RNA helicase MSS116, mitochondrial	+	-	+	+
Q5APC0	Golgi apparatus membrane protein TVP18	+	-	+	+
Q5AHG6	Serine/threonine-protein kinase SCH9	+	-	+	+
Q5AD77	Sorting nexin-4	+	-	+	+
Q5AKU3	CAP1-binding-protein	+	-	+	+
Q59NY7	Coupling of ubiquitin conjugation to ER degradation protein 1	+	-	+	-
P43102	Ubiquitin-conjugating enzyme E2 4	+	-	+	-
Q59PP6	Mediator of RNA polymerase II transcription subunit 16	+	-	+	-
Q5A7S7	Fork-head transcriptional regulator 2	+	-	+	-
Q59S50	ATP-dependent RNA helicase DBP7	+	-	+	-
Q5AJC1	Vacuolar protein-sorting protein BRO1	+	-	+	-
Q5AB93	Protein PNS1	+	-	+	-
Q5AEK8	Delta 8-(E)-sphingolipid desaturase	+	-	+	-
P46586	ATP phosphoribosyltransferase	+	-	-	+
Q92209	Homoserine kinase	+	-	-	+
Q59XQ1	3'(2'),5'-bisphosphate nucleotidase 2	+	-	-	+
Q59Q38	Peptide-N(4)-(N-acetyl-beta-glucosaminyl)asparagine amidase	+	-	-	+

Q59VR3	FK506-binding protein 3	+	-	-	+
C4YHS3	RNA polymerase II degradation factor 1	+	-	-	+
P0CB54	ATPase GET3	+	-	-	+
Q5A4W8	Bromodomain-containing factor 1	+	-	-	+
Q5A119	Chromatin modification-related protein EAF1	+	-	-	+
Q59Y41	DNA mismatch repair protein MSH3	+	-	-	+
Q5ACL9	Protein BFR2	+	-	-	+
Q5A0W6	GPN-loop GTPase 3	+	-	-	+
Q59PE7	Protein BCP1	+	-	-	+
Q5A0E2	Exportin-T	+	-	-	+
Q5AM44	mRNA 3'-end-processing protein RNA14	+	-	-	+
C4YJ78	Methylthioribose-1-phosphate isomerase	+	-	-	-
C4YTL7	Cytochrome c oxidase assembly factor 3, mitochondrial	+	-	-	-
Q5A519	EKC/KEOPS complex subunit CGI121	+	-	-	-
Q5ADL9	Defective in cullin neddylation protein 1	+	-	-	-
Q5A6Q4	tRNA (adenine(58)-N(1))-methyltransferase non-catalytic subunit TRM6	+	-	-	-
Q59JU3	Pre-rRNA-processing protein IPI1-1	+	-	-	-
Q5A416	tRNA (adenine(58)-N(1))-methyltransferase catalytic subunit TRM61	+	-	-	-
Q59PP0	DNA mismatch repair protein HSM3	+	-	-	-
Q9UW14	pH-response transcription factor pacC/RIM101	+	-	-	-
Q5A6P6	MFS antiporter QDR1	+	-	-	-
Q59Z14	Deoxyhypusine hydroxylase	+	-	-	-
Q59Y20	Protein DSE1	+	-	-	-
Q5A2K0	Stress response protein NST1	+	-	-	-
Q5A287	Transcription factor SFL1	+	-	-	-
Q5A4E3	Initiation-specific alpha-1,6-mannosyltransferase	-	+	-	-
Q5ACU3	Dolichyl-phosphate-mannose--protein mannosyltransferase 5	-	+	-	-
A0A1D8PLH0	Phosphomevalonate kinase	-	+	-	-
O74161	Chitin biosynthesis protein CHS5	-	+	-	-
Q5A2V2	Protein RMD9, mitochondrial	-	+	-	-
Q5AH25	Exocyst complex protein EXO70	-	+	-	-
Q5AHZ7	Mediator of RNA polymerase II transcription subunit 17	-	+	-	-
Q59N29	ATP-dependent rRNA helicase SPB4	-	+	-	-
Q5AAG1	Enhancer of polycomb-like protein 1	-	+	-	-
Q5AAV3	Ribosome-releasing factor 2, mitochondrial	-	+	-	-

O13401	Superoxide dismutase [Mn], mitochondrial	-	+	-	-
C4YFX2	Transcriptional repressor TUP1	-	+	-	-
Q5A7S5	Pre-mRNA-splicing factor RSE1	-	+	-	-
P43094	Candidapepsin-5	-	+	-	-
P83775	Putative NADPH-dependent methylglyoxal reductase GRP2	-	-	+	-
P52497	Carbon catabolite-derepressing protein kinase	-	-	+	-
O93807	Tubulin gamma chain	-	-	+	-
Q59NG5	Crossover junction endonuclease MUS81	-	-	+	-
Q9B8D6	NADH-ubiquinone oxidoreductase chain 1	-	-	+	-
Q5AGA0	Putative alpha-1,3-mannosyltransferase MNN1	-	-	+	-
Q59LV5	Phosphatidylethanolamine N-methyltransferase	-	-	+	-
Q5AK66	Phosphatidylserine decarboxylase proenzyme 2	-	-	+	-
P84285	Sterol O-acyltransferase 2	-	-	+	-
Q5ABC5	Phosphatidylserine decarboxylase proenzyme 1, mitochondrial	-	-	+	-
Q59SI2	Mitochondrial import inner membrane translocase subunit TIM14	-	-	+	-
P48990	Mitochondrial import inner membrane translocase subunit TIM54	-	-	+	-
Q5APA2	Golgi apparatus membrane protein TVP23	-	-	+	-
Q59ZW9	Mitochondrial import inner membrane translocase subunit TIM16	-	-	+	-
Q59WU0	Probable lysine/arginine permease CAN2	-	-	+	-
A0A1D8PK89	General amino-acid permease GAP2	-	-	+	-
Q5AAF4	Formin BNR1	-	-	+	-
P33153	GTP-binding RHO-like protein	-	-	+	-
Q59LP6	Altered inheritance of mitochondria protein 11	-	-	+	-
Q5AHC2	Mitochondrial inner membrane i-AAA protease complex subunit MGR1	-	-	+	-
C4YPQ0	Altered inheritance of mitochondria protein 24, mitochondrial	-	-	+	-
C4YRP9	Respiratory supercomplex factor 1, mitochondrial	-	-	+	-
P43073	N-(5'-phosphoribosyl)anthranilate isomerase	-	-	-	+
O74254	Glucoamylase 1	-	-	-	+
P43066	D-arabinitol 2-dehydrogenase [ribulose-forming]	-	-	-	+
Q5AJX2	Sphingolipid delta(4)-desaturase	-	-	-	+
Q5ABD9	Vacuolar protein sorting-associated protein 27	-	-	-	+
Q59RH5	Histone acetyltransferase type B subunit 2	-	-	-	+
Q5APF0	Ribosome biogenesis protein YTM1	-	-	-	+
Q5AAT0	Decapping nuclease RAI1	-	-	-	+
Q59YL9	Pre-rRNA-processing protein ESF2	-	-	-	+

Q59LF9	Methionine aminopeptidase 2	-	-	-	+
Q5ABT8	Beta-mannosyltransferase 4	-	-	-	+
Q5ADQ9	Beta-mannosyltransferase 1	-	-	-	+
C4YDC4	High osmolarity signaling protein SHO1	-	-	-	+
Q5A940	Multiprotein-bridging factor 1	-	-	-	+
C4YPS3	Regulator of rDNA transcription 14	-	-	-	+
Q59ST8	mRNA cleavage and polyadenylation factor CLP1	-	-	-	+
P84149	mRNA export factor MEX67	-	-	-	+
O93829	Geranylgeranyl transferase type-2 subunit alpha	-	-	-	+
Q5A895	Class E vacuolar protein-sorting machinery protein HSE1	-	-	-	+
A0A1D8PPK1	Probable NADPH dehydrogenase	-	-	-	+
Q5A1M3	Potential protein lysine methyltransferase SET5	-	-	-	+
Q5AKU5	Secreted beta-glucosidase SIM1	-	+	-	+
A0A1D8PH52	Acetyl-CoA acetyltransferase	-	+	-	+
Q9HF78	Glutamate--cysteine ligase	-	+	-	+
Q5A246	DNA-directed RNA polymerase III subunit RPC3	-	+	-	+
P78600	Proline--tRNA ligase, cytoplasmic	-	+	-	+
Q5AM84	U1 small nuclear ribonucleoprotein component SNU71	-	+	-	+
Q5A7M3	Kynurenine 3-monooxygenase	-	+	+	-
P23316	Chitin synthase 1	-	+	+	-
Q9B8C9	NADH-ubiquinone oxidoreductase chain 5	-	+	+	-
Q9B8D1	NADH-ubiquinone oxidoreductase chain 3	-	+	+	-
Q04782	Lanosterol synthase	-	+	+	-
P0CB63	Golgi to ER traffic protein 2	-	+	+	-
Q5A477	GDP-mannose transporter	-	+	+	-
Q9HFQ5	60S acidic ribosomal protein P2-A	-	+	+	-
Q5A061	Mitochondrial escape protein 2	-	+	+	-
Q59QC6	Assembly factor CBP4	-	+	+	-
P30574	Carboxypeptidase Y	-	+	+	+
Q5APQ8	Putative alpha-1,3-mannosyltransferase MNN12	-	+	+	+
Q5AP90	Alpha-1,2-mannosyltransferase MNN23	-	+	+	+
A0A1D8PCB9	C-8 sterol isomerase ERG2	-	+	+	+
Q5A888	3-keto-steroid reductase ERG27	-	+	+	+
Q59N80	Inosine triphosphate pyrophosphatase	-	+	+	+
Q5ACM4	Pre-rRNA-processing protein PNO1	-	+	+	+

Q59MF9	Conserved oligomeric Golgi complex subunit 6	-	+	+	+
Q5A216	Probable kinetochore protein NDC80	-	+	+	+
Q5AAJ7	SWR1-complex protein 4	-	+	+	+
Q5ANL6	13 kDa ribonucleoprotein-associated protein	-	+	+	+
Q9HFQ4	60S acidic ribosomal protein P2-B	-	+	+	+
P46614	Pyruvate kinase	-	+	+	+
Q5AD51	Ergosterol biosynthetic protein 28	-	+	+	+
Q5AIA1	Glucan 1,3-beta-glucosidase 2	-	-	+	+
Q5ALV2	Actin cytoskeleton-regulatory complex protein SLA1	-	-	+	+
Q5ALL3	tRNA-dihydrouridine(47) synthase [NAD(P)(+)]	-	-	+	+
G1UB37	Major facilitator superfamily multidrug transporter FLU1	-	-	+	+
Q59SU1	Candidapepsin-9	-	-	+	+

Table S3. The proteins identified as unique for each type of *Candida albicans* EVs

EV_{CON}	EV_{AMB}	EV_{FLU}	EV_{CASP}
Methylthioribose-1-phosphate isomerase	Initiation-specific alpha-1,6-mannosyltransferase	Putative NADPH-dependent methylglyoxal reductase GRP2	N-(5'-phosphoribosyl)anthranilate isomerase
Cytochrome c oxidase assembly factor 3, mitochondrial	Dolichyl-phosphate-mannose--protein mannosyltransferase 5	Carbon catabolite-derepressing protein kinase	Glucoamylase 1
EKC/KEOPS complex subunit CGI121	Phosphomevalonate kinase	Tubulin gamma chain	D-arabinitol 2-dehydrogenase [ribulose-forming]
Defective in cullin neddylation protein 1	Chitin biosynthesis protein CHS5	Crossover junction endonuclease MUS81	Sphingolipid delta(4)-desaturase
tRNA (adenine(58)-N(1))-methyltransferase non-catalytic subunit TRM6	Protein RMD9, mitochondrial	NADH-ubiquinone oxidoreductase chain 1	Vacuolar protein sorting-associated protein 27
Pre-rRNA-processing protein IPI1-1	Exocyst complex protein EXO70	Putative alpha-1,3-mannosyltransferase MNN1	Histone acetyltransferase type B subunit 2
tRNA (adenine(58)-N(1))-methyltransferase catalytic subunit TRM61	Mediator of RNA polymerase II transcription subunit 17	Phosphatidylethanolamine N-methyltransferase	Ribosome biogenesis protein YTM1
DNA mismatch repair protein HSM3	ATP-dependent rRNA helicase SPB4	Phosphatidylserine decarboxylase proenzyme 2	Decapping nuclease RAI1
pH-response transcription factor pacC/RIM101	Enhancer of polycomb-like protein 1	Sterol O-acyltransferase 2	Pre-rRNA-processing protein ESF2
MFS antiporter QDR1	Ribosome-releasing factor 2, mitochondrial	Phosphatidylserine decarboxylase proenzyme 1, mitochondrial	Methionine aminopeptidase 2
Deoxyhypusine hydroxylase	Superoxide dismutase [Mn], mitochondrial	Mitochondrial import inner membrane translocase subunit TIM14	Beta-mannosyltransferase 4
Protein DSE1	Transcriptional repressor TUP1	Mitochondrial import inner membrane translocase subunit TIM54	Beta-mannosyltransferase 1
Stress response protein NST1	Pre-mRNA-splicing factor RSE1	Golgi apparatus membrane protein TVP23	High osmolarity signaling protein SHO1
Transcription factor SFL1	Candidapepsin-5	Mitochondrial import inner membrane translocase subunit TIM16	Multiprotein-bridging factor 1
		Probable lysine/arginine permease CAN2	Regulator of rDNA transcription 14
		General amino-acid permease GAP2	mRNA cleavage and polyadenylation factor CLP1
		Formin BNR1	mRNA export factor MEX67
		GTP-binding RHO-like protein	Geranylgeranyl transferase type-2 subunit alpha
		Altered inheritance of mitochondria protein 11	Class E vacuolar protein-sorting machinery protein HSE1
		Mitochondrial inner membrane i-AAA protease complex subunit MGR1	Probable NADPH dehydrogenase
		Altered inheritance of mitochondria protein 24, mitochondrial	Potential protein lysine methyltransferase SET5
		Respiratory supercomplex factor 1, mitochondrial	
